# RiboMicrobe: An Integrated Translatome Atlas for Microorganism

**DOI:** 10.1002/advs.202509877

**Published:** 2025-10-13

**Authors:** Yingshun Zhou, Jinjing Luo, Xiaoqiang Lang, Yuli Gan, Guoxian Liu, Yuling Cui, Fazhi Li, Weicong Zhu, Bing Chen, Yuanyuan Dong, Yinglin Wu, Yi Cao, Qi Liu

**Affiliations:** ^1^ Department of Pathogenic Biology School of Basic Medical Sciences Southwest Medical University Luzhou 646000 P. R. China; ^2^ Rice Research Institute Guangdong Academy of Agricultural Sciences Guangzhou 510640 P. R. China; ^3^ Key Laboratory of Genetics and Breeding of High Quality Rice in Southern China (Co‐construction by Ministry and Province) Ministry of Agriculture and Rural Affairs Guangzhou 510640 P. R. China; ^4^ Guangdong Key Laboratory of Rice Science and Technology Guangzhou 510640 P. R. China; ^5^ Guangdong Rice Engineering Laboratory Guangzhou 510640 P. R. China; ^6^ Microbiology and intelligent biomanufacturing Key Laboratory of Sichuan Province College of Life Science Sichuan University Chengdu Sichuan 610041 P. R. China; ^7^ College of Life Science Hebei University Baoding 071002 P. R. China; ^8^ College of Life Sciences Jilin Agricultural University Changchun Jilin 130118 P. R. China; ^9^ School of Life Science and Technology Lingnan Normal University Zhanjiang Guangdong 524048 P. R. China

**Keywords:** prediction, prokaryotes, RiboMicrobe, ribosome profiling, translatomics, sORF

## Abstract

Ribosome profiling (Ribo‐seq) represents a significant advance in translatomics research. This technique enables the precise measurement of global and in vivo translation dynamics, the quantification of translation, and the identification of active translated small open reading frames (sORFs). While several databases have been developed to focus on the translatome, comprehensive databases dedicated specifically to analyses of translation and sORFs in prokaryotes remain scarce. Therefore, RiboMicrobe (https://rnainformatics.org.cn/RiboMicrobe/ and https://rnainformatics.cn/RiboMicrobe/) develops a comprehensive database tailored for Ribo‐seq data from prokaryotic microorganisms, Accompanying this database, it also introduces two novel sORF prediction models based on transformer‐based deep learning architecture, sORFPredRibo and sORFPred, to support translatomics analyses and sORF annotation. Currently, RiboMicrobe encompasses 891 Ribo‐seq, 369 matched RNA‐seq, and 62 proteome datasets from 36 prokaryotes and 2 viruses, and provides users with intuitive web interfaces to easily access and explore information of interest. In addition, a suite of bioinformatics tools encompassing three functional categories: visualization tools (Browse, JBrowse, and mRNABrowse) is developed for data exploration; predictive algorithms (sORFPred and sORFPredRibo) for sORFs prediction; and comparative analysis utilities (DiffTE, DiffCO, and BLAST) for functional investigations. It is believed that the diverse data and capabilities of RiboMicrobe will advance the field of microbial translational research substantially.

## Graphical abstract

RiboMicrobe is a comprehensive database for Ribo‐seq data from prokaryotes, featuring 891 Ribo‐seq, 369 RNA‐seq, and 62 proteome datasets from 38 species. The database not only provides various data visualization results but also includes two sORF prediction models and a suit of bioinformatics tools for comparative analysis, facilitating easy access and efficient query of relevant data for users.

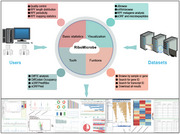

## 1 Introduction

Ribosome profiling (Ribo‐seq), first proposed in 2009,^[^ ^1^ ^]^ is a high‐throughput technique based on deep sequencing for comprehensive genome‐wide translation analyses.^[^ ^2^ ^]^ Ribo‐seq identifies and quantifies translation by detecting ribosome‐protected fragments (RPFs) and provides insights into internal translational dynamics,^[^ ^3^ ^]^ including codon usage bias,^[^ ^4^ ^]^ translational pausing,^[^ ^5^ ^]^ protein folding,^[^ ^6^ ^]^ and small open reading frames (sORFs),^[^ ^7^ ^]^ in both eukaryotic and prokaryotic organisms. The rapid advancement of Ribo‐seq has led to the discovery of numerous non‐conventional ORFs,^[^ ^8^ ^]^ such as those involved in non‐canonical translation initiation or termination in non‐coding regions.^[^ ^9^, ^10^, ^11^, ^12^ ^]^

sORFs, which are less than 100 codons long, encode small proteins or polypeptides (sORF‐encoded polypeptides, SEPs). These SEPs are involved in diverse cellular and organismal functions and regulate complex biological systems.^[^ ^13^, ^14^, ^15^ ^]^ sORFs play a regulatory role in protein complexes and function as allosteric regulators of enzymes and essential transmembrane components.^[^ ^16^, ^17^, ^18^, ^19^, ^20^ ^]^ Additionally, they regulate crucial biological processes, such as stress responses and quorum sensing in bacteria.^[^ ^21^, ^22^, ^23^, ^24^, ^25^ ^]^ Established databases based on Ribo‐seq data include Ribo‐uORF,^[^ ^26^ ^]^ sORFs.org,^[^ ^13^ ^]^ TranslatomeDB,^[^ ^27^ ^]^ SmProt,^[^ ^28^, ^29^ ^]^ OpenProt,^[^ ^30^ ^]^ PsORF,^[^ ^31^ ^]^ riboseq.org,^[^ ^32^ ^]^ nORFs.org,^[^ ^33^ ^]^ RIBOBASE (http://www.bioinf.uni‐freiburg.de/~ribobase/index.html), etc. Moreover, several databases store sORF information, such as uORFdb,^[^ ^34^ ^]^ MetamORF,^[^ ^35^ ^]^ and ARA‐PEPs.^[^ ^36^ ^]^ However, these databases predominantly focus on eukaryotic organisms, including animals and plants, with very limited information on bacteria (e.g., *Escherichia coli* and *Bacillus subtilis*) and pathogens.^[^ ^37^ ^]^

Here, we established the RiboMicrobe database (https://rnainformatics.org.cn/RiboMicrobe/ and https://rnainformatics.cn/RiboMicrobe) along with novel sORF prediction models, to facilitate translation analysis in prokaryotic microorganisms based on Ribo‐seq datasets. To the best of our knowledge, RiboMicrobe is the first comprehensive translatome database for prokaryotic taxa; it contains 891 Ribo‐seq, 369 matched RNA‐seq, and 62 proteome datasets for 38 species. We curated additional relevant datasets from previous research and related resources, such as various types of RNA modifications (m^1^A, m^5^C, m^6^A, m^7^G, and pseudouridine) and riboswitches influencing microbial RNA translation. Furthermore, RiboMicrobe offers a variety of visualization tools and useful functionalities, such as mRNAbrowse for displaying transcriptional profiles of potential sORFs, JBrowse for showing all RNA‐seq samples and other publicly available genome annotations, PepViewer for obtaining mass spectrometry information of sORFs, sORFPred and sORFPredRibo for predicting sORFs, DiffTE for differential translation analyses, DiffCO for analyzing differential codon usage, and Blast for sORF homology analyses. RiboMicrobe addresses the current lack of prokaryotic‐specific translation databases and serves as a foundational platform that enables new discoveries in small ORF function, translational regulation, and microbial genome annotation. In summary, we expect RiboMicrobe to promote research in microbial translatomics by providing comprehensive resources and tools.

## 2 Results

### 2.1 Overview of the RiboMicrobe Database

**Figure 1:**
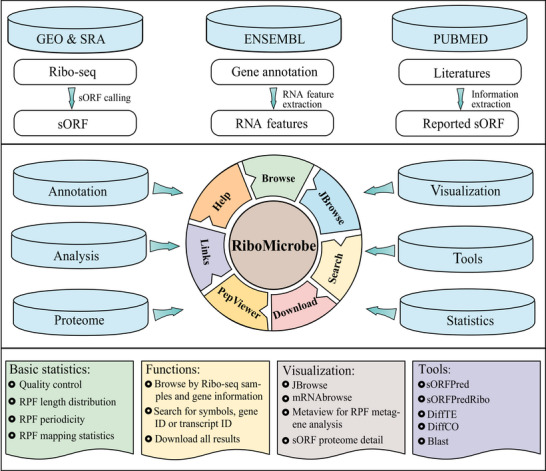
Overall workflow of RiboMicrobe. RiboMicrobe focuses on extensive and integrative translation analyses and information on sORF for prokaryotic taxa. The database includes 891 Ribo‐seq, 369 matched RNA‐seq, and 62 proteome datasets and sORF‐encoded micropeptides for various microorganisms. RiboMicrobe includes related annotation datasets such as RNA modifications and riboswitches. All results generated by RiboMicrobe were deposited in a MySQL database and are displayed in several convenient modules on the website. Furthermore, RiboMicrobe provides several convenient tools for visualizing and analyzing data.

RiboMicrobe serves as a comprehensive database of prokaryotic microbial translation information, with 891 Ribo‐seq and 369 matched RNA‐seq datasets downloaded from GEO and SRA databases for 38 species, including *Acetobacterium woodii*, *Bacillus subtilis*, *Bacteroides thetaiotaomicron* VPI5482, *Caulobacter vibrioides*, *Clostridium aceticum*, *Clostridium drakei*, *Escherichia coli, etc*. (Table S1, Supporting Information). In addition, RiboMicrobe integrates data for riboswitches and other RNA modification datasets, such as m^1^A, m^6^A, m^7^G, m^5^C, and pseudouridine. RiboMicrobe also includes validated sORFs reported in literatures (Table S2, Supporting Information). All data have been subjected to standardized processing and are presented in intuitive web modules for users (**Figure** **1**).

### 2.2 Web Interface Modules Developed in RiboMicrobe

The “Browse” module contains three functions: i) Browse of gene information, ii) Browse of Ribo‐seq data, and iii) Browse of reported micropeptides. “Browse of gene information” shows the detail information of gene, including hyperlinks to individual gene profiles, Metaview for visualizing RPF metagene distribution, and mRNAbrowse for candidate ORF visualization. “Browse of Ribo‐seq data” provides detailed information on each Ribo‐seq dataset, with links to the reference publication and detailed Ribo‐seq information (basic statistics, periodicity and metagene distribution, translation efficiency, and ORF list). The “Browse of reported micropeptides” page displays detail information on experimentally reported micropeptides from the literature.

The “Search” module allows users to obtain detailed information by submitting a transcript ID and gene symbol. Users can also search through the quick‐search field on the RiboMicrobe homepage.

**Figure 2:**
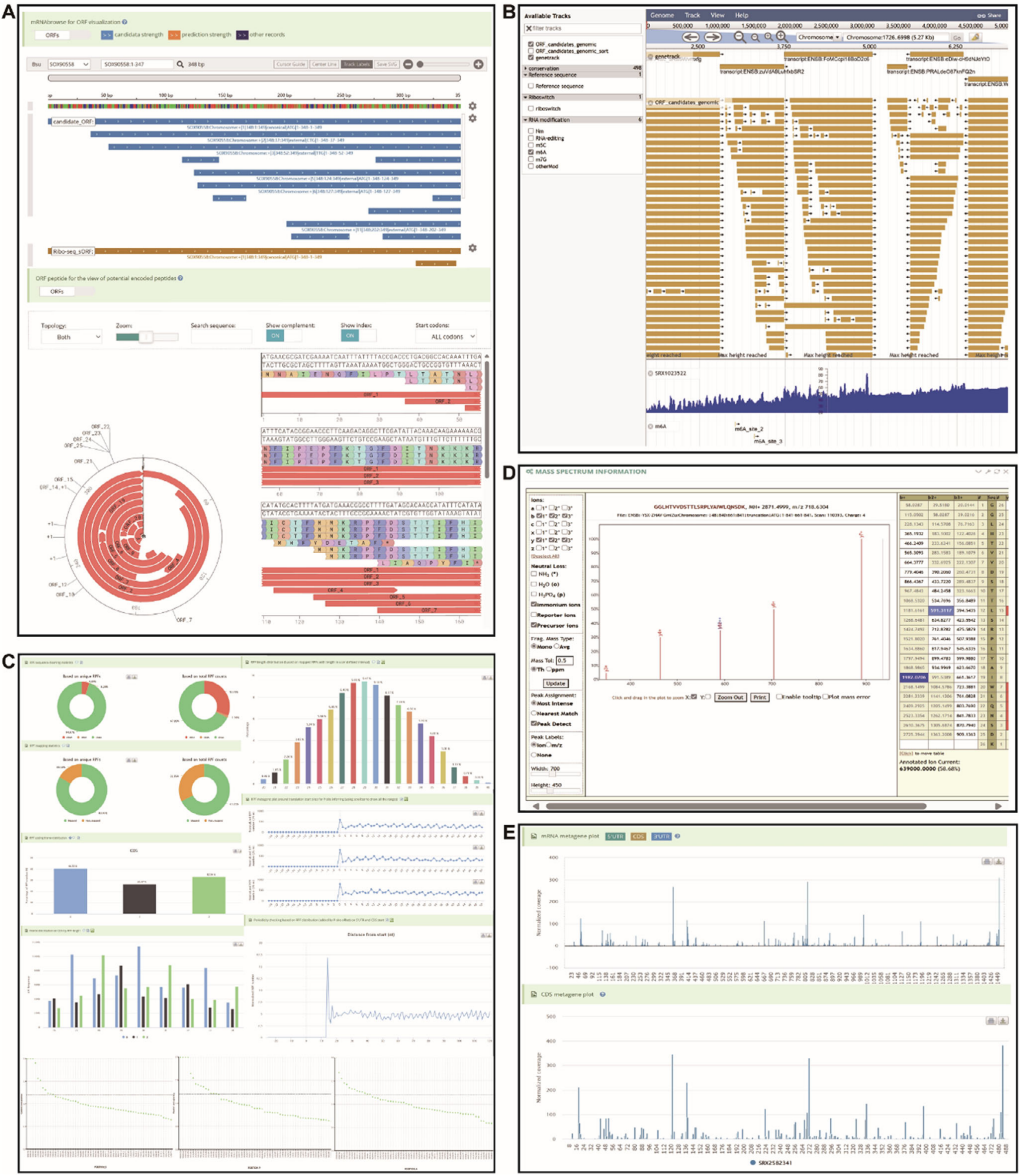
Visualization of the RiboMicrobe database. A) Visualization of mRNAbrowse showing tracks of candidate ORFs and potential ORFs within an individual gene. B) Visualization of JBrowse data displaying tracks of all candidate ORFs across the entire genome, coverage of Ribo‐seq sample reads, various RNA modification sites and riboswitches at the genome‐wide scale. C) PRF analysis results for each Ribo‐seq sample using the Ribotoolkit. D) Mass spectrometry results for sequence peptides, viewed using the Lorikeet MS/MS viewer. E) RPF metagene profile for the whole transcript and CDS region of a specific gene.

The “Visualization” module includes four parts. i) mRNAbrowse offers an intuitive interface to show the distribution of candidate and potential ORFs at the transcript level. ii) JBrowse is used for the detailed visualization of all candidate ORFs across the genome, depicting the coverage of Ribo‐seq sample reads, various RNA modification sites, and riboswitches. iii) Basic statistics provides the translation efficiency and ORF results for Ribo‐seq samples. iv) Mass spectrometry data are provided for micropeptides (**Figure** **2**).

Other modules include i) the “Statistics” module, which contains sample‐specific statistics and comprehensive statistics for sORF. ii) The “Download” module provides access to files for results of diverse translatome analyses of Ribo‐seq samples, sORF lists, and annotation datasets. iii) The “Links” module displays software and database links associated with RiboMicrobe, facilitating user navigation and inquiries.

### 2.3 PepViewer Interface Developed in RiboMicrobe

The PepViewer module is designed to help users evaluate theoretically possible sORFs and consists of two subpages: RibORF‐identified proteins and sORFPredRibo‐identified proteins. RibORF‐identified proteins primarily displays the MS support for the sORFs predicted by RibORF2.0.^[^ ^38^ ^]^ By intersecting the peptide fragments obtained from MaxQuant and FragPipe mass spectrometry analysis with the predicted sORFs, a detailed table is generated. The table provides information including species, ID, gene, length, MaxQuant support, FragPipe support, ORFScore count, and sequence. MaxQuant support can be linked to the Lorikeet MS/MS viewer for MS results, while FragPipe support is also clickable for further details. sORFPredRibo‐identified page displays the intersection of sORFs predicted by sORFPredRibo and FragPipe peptides, with clickable sequence linking to detailed peptide information. Combining predicted sORFs with MS data enhances the reliability and confidence of the identified sORFs, providing valuable reference for users (Figure S1, Supporting Information).

### 2.4 Web‐Based Analytical Tools Developed in RiboMicrobe

Five convenient tools (sORFPred, sORFPredRibo, DiffTE, DiffCO, Blast, and Retrieve results) were developed for users to perform various analyses in RiboMicrobe.

**Figure 3:**
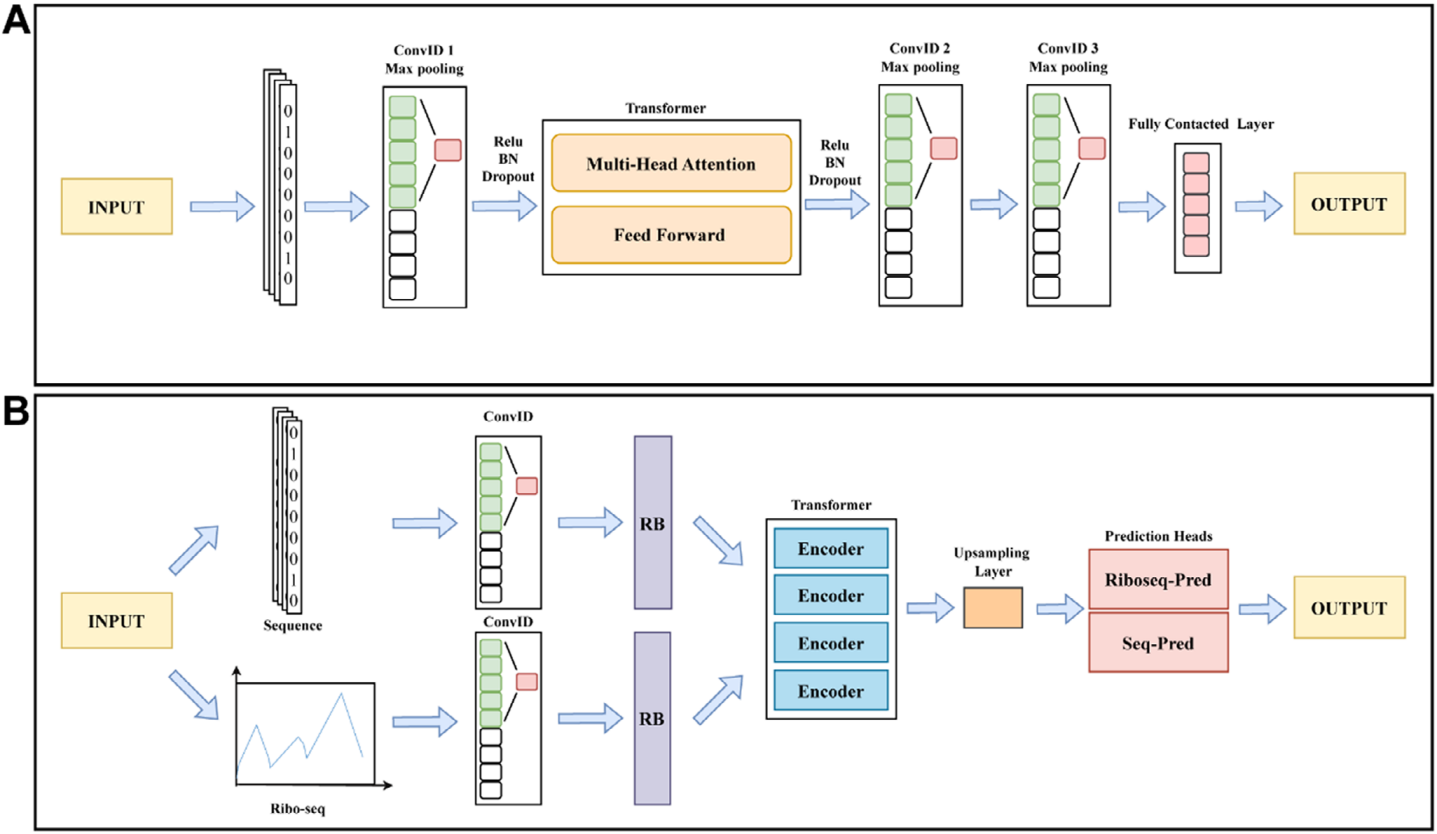
The model architectures of sORFPred and sORFPredRibo. A) The architecture of sORFPred is primarily based on CNN and Transformer. B) sORFPredRibo is a multimodal deep learning model that includes components such as CNN, Residual Blocks, Transformer encoder, upsampling layers, and prediction heads.

sORFPred uses convolutional neural networks (CNN) and Transformer models to learn the feature patterns of initiation sequence fragments from 36 bacterial species, predicting all potential TIS fragments and thereby identifying potential sORFs. sORFPred relies solely on sequence features, and its model architecture is shown in **Figure** **3**A. Users only need to upload the sequence to make predictions on the website, and the result file primarily includes the predicted TIS and sORF results (Figure S2, Supporting Information).

sORFPredRibo is a multimodal deep learning model that includes components such as CNN, Residual Blocks, Transformer encoder, upsampling layers, and prediction heads, combining sequence features and Ribo‐seq signal features for prediction. Its model architecture is shown in Figure 3B. Users need to upload a sequence file and select the BAM file of one of the 24 species we provide for prediction. Web‐based predictions may take some time, and users can also download the script to perform predictions using their own BAM file (https://github.com/rnainformatics/RiboMicrobe). The result files include all prediction results and sORF predictions for sequences shorter than 300 nucleotides (Figure S3, Supporting Information).

DiffTE is designed to analyze differences in translation efficiency between two conditions. Upon selecting a species, a table displaying sample information for all Ribo‐RNA pairs in the species are shown and users can assign samples from the same project into two groups. Volcano plots, heatmaps, and boxplots are generated to compare translation efficiencies (Figure S4, Supporting Information). Genes with significant differences in translation efficiency are shown in a table.

DiffCO enables comparisons of codon occupancy between two conditions. Users can assign Ribo‐seq samples from the same project into two groups. Differences in codon occupancy are shown for the three ribosome‐binding sites, E (exit site), P (peptidyl transferase center), and A (aminoacyl site), along with positions A+1, A+2, and A+3 downstream of site A. The DiffCO output includes visual representations, such as volcano plots, heatmaps, and boxplots (Figure S5, Supporting Information).

Blast is used for homology analyses of sORF. Users can upload nucleotide sequences or amino acid sequences in FASTA format to search against candidate ORFs. Results are displayed in tabular format, including query ID, subject ID, gene location, sequence identity, alignment length, query start/end positions, subject start/end positions, and E‐values. Users can download detailed result files for further analyses (Figure S6, Supporting Information).

Additionally, the“Retrieve results”interface allows users to access processed analytical outputs by job ID, especially for tools like DiffTE and sORFPredRibo, which may have longer waiting times.

### 2.5 Comparative Analysis of Prediction Models

We systematically predicted and evaluated potential sORFs in *Bacillus subtilis* using both DeepRibo and sORFPredRibo. DeepRibo is one of the most widely used tools for sORF prediction.^[^ ^39^ ^]^ It integrates Ribo‐seq data and TIS information within a CNN‐RNN architecture to identify ORFs. Specifically, the CNN module analyzes the promoter region and sequences flanking the start codon, while the RNN component models the Ribo‐seq coverage profiles surrounding the translation initiation site. In contrast, sORFPredRibo adopts a CNN‐Transformer architecture, which enables the model to capture local sequence features and handle long‐range dependencies effectively. sORFPredRibo is initially pre‐trained on datasets from 19 species and subsequently fine‐tuned on the target species, enabling it to learn conserved sequence features and translational signals across diverse taxa.

**Figure 4:**
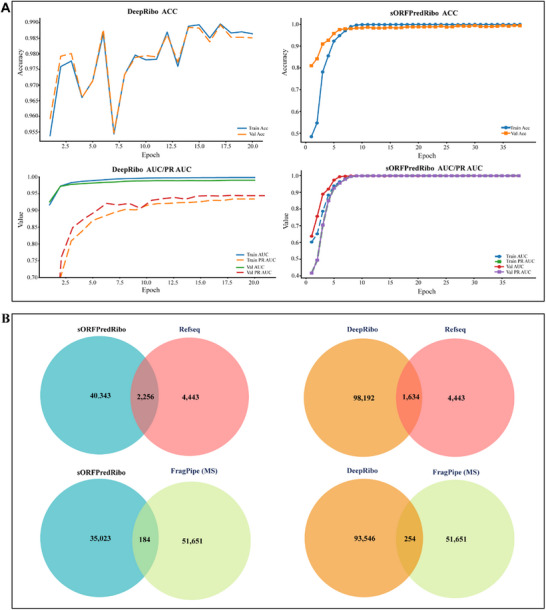
Comparison between sORFPredRibo and DeepRibo. A) The performance evaluation of sORFPredRibo and DeepRibo includes a comparison of ACC, AUC, and PR AUC. B) Venn diagrams illustrate the distribution of sORFPredRibo and DeepRibo predictions in relation to RefSeq annotations and MS data.

In terms of model performance, DeepRibo achieved an accuracy (ACC) of 0.983 on the validation set, with an AUC of 0.990 and a PR AUC of 0.894. In comparison, the best fine‐tuned version of sORFPredRibo outperformed DeepRibo, achieving an ACC of 0.992, with both AUC and PR AUC reaching 0.999 (**Figure** **4**A). With respect to prediction results, DeepRibo identified a total of 98,192 valid ORFs, of which 1,634 (1.66%) overlapped with RefSeq annotations. Among them, 93,546 were sORFs shorter than 300 nt, and 254 (0.27%) were supported by FragPipe‐based mass spectrometry evidence. In contrast, sORFPredRibo predicted 40,343 ORFs, including 2,265 (6.46%) overlapping with RefSeq annotations. Among the 35,023 sORFs shorter than 300 nt, 184 (0.53%) were supported by FragPipe. Notably, among 10,283 sORFs predicted with a probability greater than 0.99, 156 (1.53%) matched peptides detected by FragPipe (Figure 4B).

We further evaluated the performance of both models using the Escherichia coli SRX7101497 dataset. The results demonstrated that sORFPredRibo exhibited more stable prediction performance. On the validation set, DeepRibo achieved an ACC of 0.984, an AUC of 0.990, and a PR AUC of 0.894, whereas sORFPredRibo achieved an ACC of 0.9901, an AUC of 0.996, and a PR AUC of 0.994 (Figures S7A and S8B, Supporting Information). In terms of ORF identification, sORFPredRibo predicted 54,428 ORF fragments, among which 3,256 (5.96%) overlapped with annotated ORFs. Of the 45,675 predicted sORF fragments, 116 (0.25%) were supported by FragPipe evidence; the same 116 were retained when filtering for predictions with probability scores greater than 0.99, representing 0.32%. In comparison, DeepRibo predicted a total of 115,254 ORF fragments, of which only 1,824 (1.58%) were annotated. Among the 108,371 sORFs shorter than 300 nt, only 156 (0.14%) had supporting evidence from FragPipe. (Figure S7B, Supporting Information). Collectively, these results suggest that sORFPredRibo outperforms DeepRibo in both predictive accuracy and the ability to identify potentially functional sORFs.

sORFPred was a lightweight prediction model that demonstrated relatively stable overall predictive performance. We further evaluated its predictions by comparing them to RefSeq annotations and mass spectrometry data. In Bacillus subtilis, sORFPred predicted a total of 192,275 ORF fragments, of which 3,231 (1.68%) overlapped with annotated ORFs. Among the 165,955 predicted sORF fragments, 108 (0.065%) were supported by FragPipe evidence. Following deduplication, the number of predicted ORFs was reduced to 57,662, with 2,475 (4.29%) overlapping annotated ORFs. The number of unique predicted sORFs decreased to 85,572, with 108 (0.12%) still supported by FragPipe. In Escherichia coli, sORFPred predicted 214,675 ORF fragments, 3,650 (1.70%) of which overlapped annotated ORFs. Of the 181,021 predicted sORF fragments, 60 (0.033%) were supported by FragPipe. After deduplication, the total number of ORFs decreased to 60,646, with 2,993 (4.94%) overlapping annotated ORFs. The number of sORFs was reduced to 94,494, with 60 (0.063%) supported by FragPipe evidence (Figure S9, Supporting Information).

sORFPred was originally designed as a sequence‐based preliminary screening tool for species or samples lacking Ribo‐seq data. Therefore, when Ribo‐seq data are available, we recommend using the sORFPredRibo model to achieve more accurate predictions with stronger biological support.

Furthermore, we observed that a subset of micropeptides previously reported in the literature were also recovered in the prediction results of both sORFPredRibo and sORFPred, providing an additional, independent validation of the accuracy and robustness of our models (Table S3, Supporting Information).

### 2.6 DiffTE Analysis of Translation Efficiency under Different Experimental Conditions

**Figure 5:**
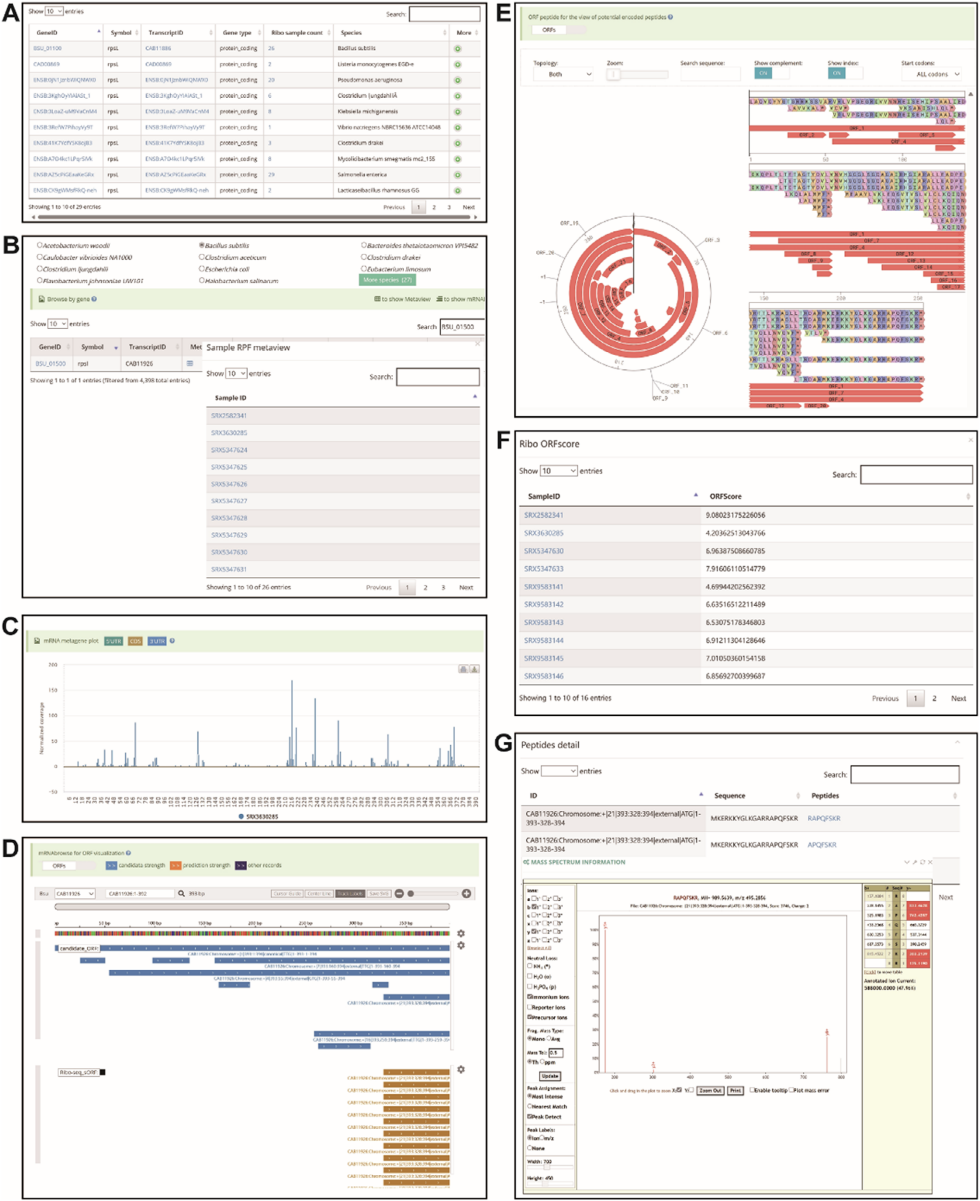
Comprehensive analysis of the *rpsI* gene in RiboMicrobe. A) Outputs of the “Search” module in RiboMicrobe revealed that *rpsI* can be queried in 27 species. B) All 26 Ribo‐seq samples of *Bacillus subtilis* for *rpsI*. C) RPF metagene profile. D) Visualization of mRNAbrowse showing tracks of candidate ORFs and potential ORFs within *rpsI*. E) Circular sequence viewer and linear sequence viewer for candidate ORFs. F) ORFscore of the micropeptide. G) Mass spectrometry results for the micropeptide, visualized using the Lorikeet MS/MS viewer.

The *rpsI* gene encodes the 30S ribosomal subunit S9 protein, which plays an important role in protein synthesis.^[^ ^40^ ^]^ The RiboMicrobe database includes *rpsI* data for 27 species (**Figure** **5**A). All 26 Ribo‐seq samples from *Bacillus subtilis* confirmed the translation of *rpsI* (CAB11926) (Figure 5B,C). Detailed information can be accessed via hyperlinks in the gene ID column. Additionally, mRNAbrowse for this gene showed distributions of candidate ORFs and active translated sORF (Figure 5D). The circular and linear sequence viewers highlight the distribution of candidate sORFs (Figure 5E). Furthermore, the ORFscore confirmed a potential sORF within *rpsI*. The PepViewer page offers micropeptide information through the transcript ID (CAB11926), including the ORFscore (Figure 5F) and MS results with the Lorikeet MS/MS viewer (Figure 5G).

**Figure 6:**
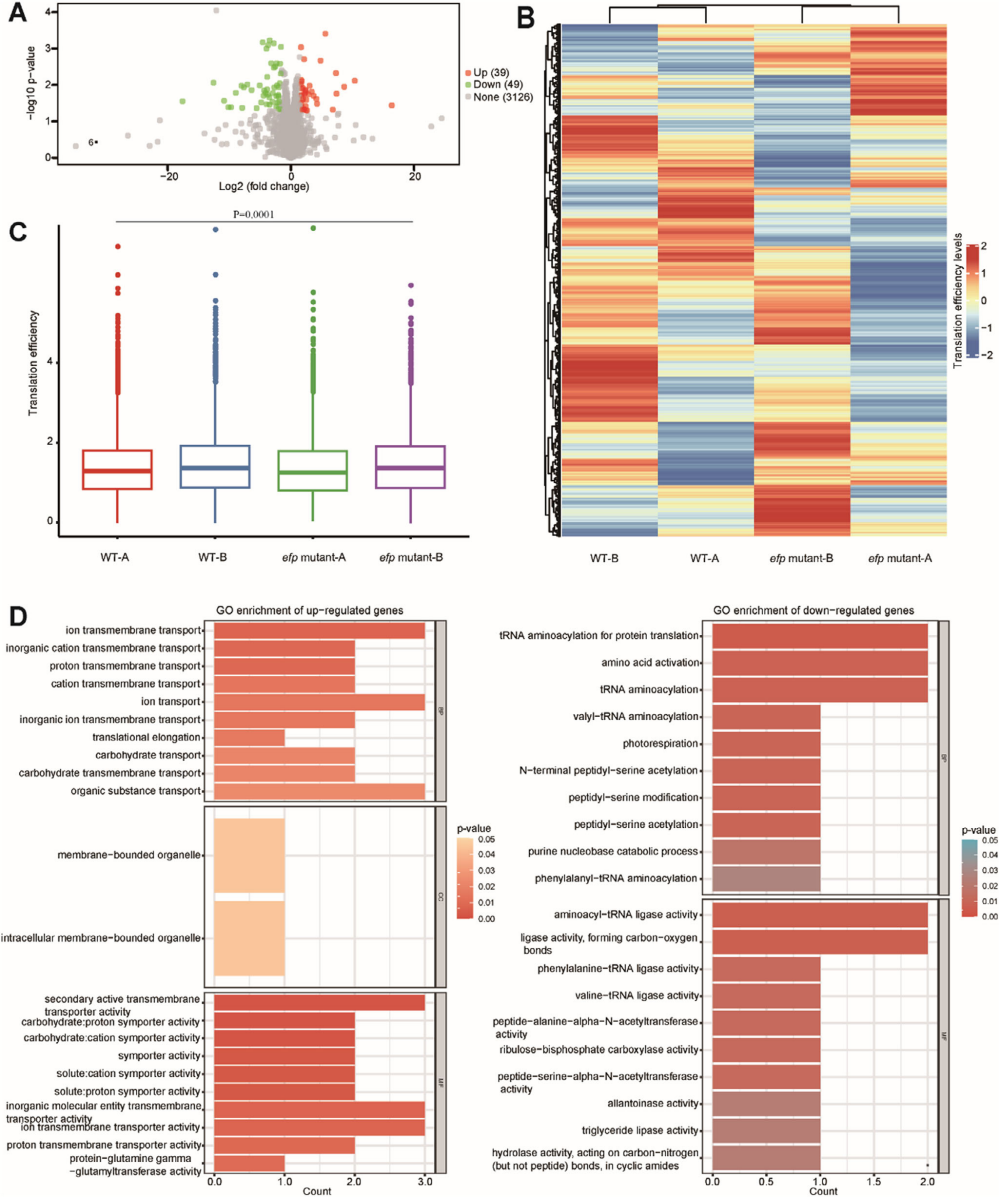
Application of the DiffTE tool in RiboMicrobe to study differential translation efficiency across different conditions: wild type (SRX5347624 and SRX5347625) and *efp* mutant (SRX5347627 and SRX5347628). A) Volcano plots. B) Heatmap. C) Boxplot. D) GO enrichment analysis of up‐regulated and down‐regulated genes.

We compared the translation efficiency of 26 Ribo‐seq samples containing the *rpsI* gene in *Bacillus subtilis* using DiffTE in RiboMicrobe. A comparison between wild type (DK1042) and *efp* mutant (DK2050) revealed 39 up‐regulated genes and 49 down‐regulated genes using 2 logarithmic fold change (log2 fold change, logFC) threshold of 1.5 (**Figure** **6**A–C; Table S4, Supporting Information). A Gene Ontology (GO) analysis showed that the up‐ and down‐regulated genes were involved in various biological processes and molecular functions. In the cellular components category, up‐regulated genes were enriched in membrane‐bounded organelle and intracellular membrane‐bounded organelle, while the down‐regulated genes did not show functional enrichment (Figure 6D).

### 2.7 DiffCO Analysis of Ribosome Pausing and Codon Preference in *Haloferax volcanii*

**Figure 7:**
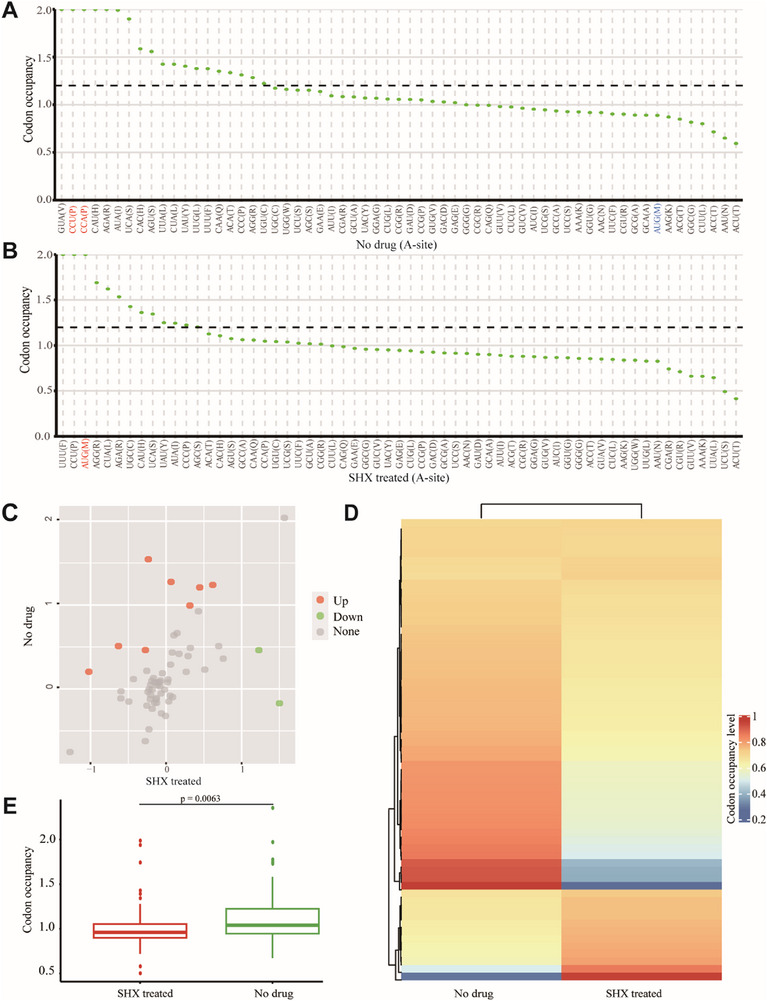
Application of the DiffCO tool in RiboMicrobe to study differential codon occupancy between SHX‐treated (SRX7007426) and untreated (SRX7007423) samples. A) Codon preference at the A‐site under untreated conditions. B) Codon preference at the A‐site under SHX‐treated conditions. C) Volcano plots of significant differences in other codons. D) Heatmap of results for other codons. E) Boxplot.

Gelsinger et al. manipulated ribosome activity using translational inhibitors in *Haloferax volcanii* and found that the ribosome pauses at the A and P‐sites in untreated samples at Proline (Pro), whereas methionine (Met) arrest was specific to the A‐site of the ribosome after treatment with serine hydroxamate (SHX).^[^ ^41^ ^]^ We examined their samples using DiffCO by comparing the SHX‐treated and untreated conditions, focusing on the codon at the A site. The codon preference for Pro (P) was evident in the untreated samples, while AUG‐encoded Met (M) showed a strong preference in SHX‐treated samples (**Figure** **7**A). Furthermore, other codons, such as GUA (Val), UCU (Ser), and UUA (Leu), exhibited significant differences between the two samples (Figure 7B).

## 3 Conclusion

Several databases focus on translatome data. For example, uORFdb is a comprehensive literature database on eukaryotic uORFs biology from PubMed.^[^ ^34^ ^]^ Ribo‐uORF, a comprehensive functional resource for uORF analyses based on Ribo‐seq data, contains data for six eukaryotes.^[^ ^26^ ^]^ sORFs.org is a public repository that identifies sORFs by Ribo‐seq analyses.^[^ ^13^ ^]^ SmProt integrates various small proteins from UTRs and ncRNA.^[^ ^28^ ^]^ ARA‐PEPs serves as a plant‐specific sORF database primarily for *Arabidopsis thaliana*.^[^ ^36^ ^]^ OpenProt supports a multi‐exon model for eukaryotic genes across 10 species.^[^ ^30^ ^]^ PsORF is a database for plant multi‐omics analysis, identifying sORFs across 35 plant species.^[^ ^31^ ^]^ RiboSeq.Org offers a suite of integrated tools for processing and analyzing Ribo‐seq data, including RiboGalaxy,^[^ ^32^ ^]^ GWIPS‐viz,^[^ ^42^ ^]^ and Trips‐Viz.^[^ ^43^, ^44^ ^]^ TranslatomeDB is a comprehensive platform for translational and proteomic studies, which provides collection and integrated analysis of published and user‐generated translatome sequencing data.^[^ ^27^ ^]^ RPFdb provides comprehensive genomic information on translated mRNAs based on ribosome profiling.^[^ ^45^ ^]^ Compared with these databases, RiboMicrobe is a prokaryotic microbial database that specifically integrates translation information (such as Ribo‐seq quality, RPF periodicity, RPF metagene, translation efficiency, codon occupancy) and sORF analysis based on Ribo‐seq dataset. Moreover, RiboMicrobe provides diverse information on Ribo‐seq data and integrates several convenient tools, such as sORFPred, sORFPredRibo, DiffTE, DiffCO, and Blast (for data analysis) as well as JBrowse, mRNAbrowse, and Lorikeet MS/MS viewer (for visualization) (**Table** **1**).

**Table 1:** Comparison with other related databases.

Name	RiboMicrobe	RPFdb	TranslatomeDB	RiboSeq.Org	uORFdb	Ribo‐uORF	sORFs.org	SmProt	ARA‐PEPs	OpenProt	PsORF
Species type	Microorganism	Eukaryote and Prokaryote	Eukaryote and Prokaryote	Eukaryote and Prokaryote	Eukaryote	Eukaryote	Eukaryote	Eukaryote and Prokaryote	Plant	Eukaryote	Plant
Data resources											
Number of Species (Prokaryote^a)^)	38 (36)	34 (13)	25 (7)	96 (27)	13 (0)	6 (0)	6 (0)	8 (1)	1 (0)	10 (0)	35 (0)
Ribo‐seq (Prokaryote)	891 (880)	5018 (533)	3288 (251)	11922 (1039)	0 (0)	1495 (0)	78 (0)	419 (Un)	0 (0)	87 (0)	103(0)
RNA‐seq (Prokaryote)	369 (366)	2343 (Un^b)^)	2415 (214)	685 (56)	0 (0)	0 (0)	0 (0)	0 (0)	48 (0)	Un (0)	Un (0)
Proteome datasets	75	0	0	Un	0	0	Un	638958	Un	114	93
Correlated RNA modification	Yes	No	No	No	No	Yes	Yes	No	No	No	No
Tools											
Comparative analysis	Yes	No	Yes	Yes	Yes	Yes	Yes	Yes	Yes	Yes	Yes
Supporting user download	Yes	Yes	Yes	Yes	Yes	Yes	Yes	Yes	Yes	Yes	No
Supporting user search	Yes	Yes	Yes	Yes	Yes	Yes	Yes	Yes	Yes	Yes	Yes
Translation efficiency	Yes	Yes	Yes	No	No	No	No	No	No	No	No
Codon occupancy	Yes	Yes	No	No	No	No	No	No	No	No	No
Visualizations											
mRNAbrowse	Yes	No	No	Yes	No	Yes	No	No	No	No	No
Gene coordinates	Yes	Yes	No	Yes	Yes	Yes	Yes	Yes	Yes	Yes	Yes
Peptide Visualizations	Yes	No	No	No	No	Yes	Yes	Yes	Yes	Yes	Yes
Ribo‐seq metagene	Yes	No	No	Yes	No	Yes	No	No	No	No	No
URL	https://rnainformatics.org.cn/RiboMicrobe	http://sysbio.sysu.edu.cn/rpfdb	http://www.translatomedb.net	https://riboseq.org/	https://www.bioinformatics.uni‐muenster.de/tools/uorfdb	http://rnainformatics.org.cn/RiboUORF	http://www.sorfs.org	http://bigdata.ibp.ac.cn/SmProt/index.html	https://github.com/rashmihazarika/ARA‐PEPs.git	https://www.openprot.org/	http://psorf.whu.edu.cn/

^a)^ The number of Prokaryote in the sample;

^b)^ Un means no specific number found.

Ribosome profiling, also known as Ribo‐seq, involves deep sequencing of RPFs and serves as a powerful technique for comprehensively monitoring protein translation in microorganisms.^[^ ^1^ ^]^ This classic molecular method utilizes ribosome footprints to measure global and in vivo translation quantitatively. Beyond capturing translational changes, Ribo‐seq also enables the discovery of novel sORFs, particularly those involved in non‐canonical translation.^[^ ^46^ ^]^ Despite increasing interest in translatomics research and sORF identification, there remains a notable absence of comprehensive databases focused on prokaryotes. To address this gap, we developed RiboMicrobe to provide in‐depth prokaryotic translation and sORF‐related data, integrating findings from previous researches and other resources. RiboMicrobe offers a comprehensive suite of tools for efficient data querying and visualization, along with two advanced sORF prediction models: sORFPredRibo and sORFPred. sORFPredRibo integrates both sequence features and translational signals, achieving superior performance over DeepRibo across multiple evaluation metrics, with higher annotation concordance and stronger mass spectrometry support. These results highlight its robustness and generalizability across diverse bacterial species. In scenarios where Ribo‐seq data are unavailable, we provide a lightweight model, sORFPred, which enables coarse‐grained sORF discovery based solely on sequence information, offering researchers an alternative for data‐limited conditions. The combination of these two models provides an effective solution for flexible and accurate sORF discovery in both data‐rich and data‐limited contexts. With its scalable tools and diverse datasets, RiboMicrobe lays the groundwork for future research in microbial gene regulation, evolutionary studies, and functional annotation across diverse prokaryotic species. We are committed to ongoing updates of RiboMicrobe to support a broader array of prokaryotic species. We expect RiboMicrobe to be an important resource in microbial translatome research and to serve as a valuable reference for other translatomics‐related databases.

## 4 Experimental Section

### Ribo‐seq Datasets and Reference Data

All Ribo‐seq data and matched RNA‐seq data were collected from the Sequence Read Archive (SRA) of NCBI (https://www.ncbi.nlm.nih.gov/sra) and Genome Sequence Archive (GSA) of the CNCB‐NGDC database. Reference genomes and gene annotation files were downloaded from the RefSeq database^[^ ^47^ ^]^ and Ensembl Bacteria database^[^ ^48^ ^]^ (version 112) (Table S1, Supporting Information). mRNA, rRNA, and tRNA sequences were retrieved from non‐coding RNA annotations in the Ensembl database (version 112).^[^ ^48^ ^]^

### Data Preprocessing

Ribo‐seq and matched RNA‐seq datasets downloaded from NCBI were converted from SRA format to FASTQ format using SRA‐Toolkit v0.0.13 (https://github.com/ncbi/sra‐tools). Cutadapt v1.18 (parameters: ‐j 10 ‐m 18 ‐M 40 ‐a adapters ‐e 0.18 –max‐n 1)^[^ ^49^ ^]^ and FASTX‐Toolkit v0.0.13 (parameters: ‐Q 33 ‐q 30 ‐p 98 ‐z) (http://hannonlab.cshl.edu/fastx_toolkit/) were employed for quality control. Clean reads of Ribo‐seq were then collapsed into FASTA format using an in‐house script. To eliminate tRNA and rRNA contamination, the clean reads were mapped against tRNA and rRNA sequences using Bowtie v1.3.1^[^ ^50^ ^]^ with default parameters.

### Data Analysis

The preprocessed Ribo‐seq data in FASTA format and RNA‐seq data in FASTQ format were used as inputs for translatome analysis using RiboToolkit.^[^ ^51^ ^]^ The analysis pipeline included Ribo‐seq quality control, RPF periodicity evaluation, RPF metagene analysis, determination of translation efficiency, codon occupancy calculation, metagene analysis and sORF prediction (Figure S10, Supporting Information). The analytical workflow integrates a diverse set of tools, including in‐house developed models (sORFPred, sORFPredRibo, and DiffCO), as well as tools adapted and optimized from existing software packages, such as RibORF^[^ ^38^ ^]^ and DiffTE. In addition, publicly available tools developed by other research groups, including BLAST and DeepRibo, were incorporated to enhance functionality and cross‐validation (Table S5, Supporting Information).

### sORF Prediction

We employed RibORF^[^ ^38^ ^]^ along with our custom‐developed models sORFPredRibo and sORFPred, to perform sORF prediction. RibORF was originally implemented using a logistic regression framework, where a linear classifier evaluated the translational potential of predicted ORFs. As recommended by the authors, pred.value ≥ 0.7 was adopted as the threshold to identify actively translated ORFs. Given that this study focuses on prokaryotic organisms, the original RibORF script was modified to better suit our data, incorporating an additional scoring metric, ORFscore, as a complementary indicator. ORFscore^[^ ^7^ ^]^ is a statistical measure designed to quantify the strength of the trinucleotide periodicity of RPFs within a given ORF. Higher ORFscore values reflect a stronger enrichment of Ribo‐seq reads in a dominant reading frame, typically the first frame, thereby providing additional evidence of active translation.

The model, sORFPredRibo, integrates CNN, residual blocks, a Transformer encoder, and upsampling layers. This architecture enables the model to effectively capture both local sequence features and global patterns within Ribo‐seq signals, thereby improving the prediction of translated sORFs. Additionally, sORFPred was developed to facilitate sORF prediction for users who only had sequence data and did not have access to Ribo‐seq datasets. sORFPred adopts a CNN‐Transformer architecture and is designed to identify potentially translatable sORFs based solely on sequence features. Comprehensive model details and configuration parameters can be accessed on GitHub (https://github.com/rnainformatics/RiboMicrobe).

### Model Architecture and Training

sORFPred performs a rough prediction of translation initiation site (TIS) and sORFs based solely on sequence features, while sORFPredRibo incorporates both sequence features and Ribo‐seq signal coverage to predict sORFs.

The training dataset for sORFPred consists of initiation codon fragments from 36 bacterial species. Sequence fragments within a 50nt upstream and 20nt downstream range of the start codons (ATG, GTG, TTG) are extracted as the positive set, while sequences with the CTG start codon of the same length are used as the negative set. The data is shuffled and then divided into training, validation, and test sets. After one‐hot encoding, the data is processed using CNN and Transformer models for learning and prediction. Specifically, in the Transformer component, the self‐attention mechanism is defined as:

(1) $$Attention\;\left(Q,K,V\right)=softmax\left(\frac{QK^{T}}{\sqrt{d_{k}}}\right)V$$

where Q, K, and V are the query, key, and value matrices, and *d_k_* is the dimension of the key vectors. This mechanism enables the model to capture contextual dependencies across the input sequence, which is crucial for identifying conserved patterns around initiation sites.

The positive training samples for sORFPredRibo are derived from the CDS regions, adhering to the valid ORF criteria. Negative samples are generated through reverse complement sequences and frame‐shift mutations, with their quantity adjusted according to a specified ratio (default 2). The average coverage for each gene is calculated based on reads in the BAM file, and the samples are labeled as either positive or negative (1 and 0) depending on the presence or absence of coverage. sORFPredRibo is pre‐trained on datasets from 19 species to obtain optimized weights, which are then fine‐tuned on individual datasets. If a candidate ORF satisfies both the sequence and Ribo‐seq signal criteria, it is classified as positive, forming a binary classification task. This primarily fine‐tuned the model using data from *Escherichia coli*, *Staphylococcus aureus*, *Bacillus subtilis*, and *Salmonella enterica*, with all results derived from these optimized, fine‐tuned models.

### Model Performance Evaluation

During training, several evaluation metrics for sORFPred include training loss, accuracy, precision, ROC‐AUC, recall, and F1 score. For sORFPredRibo, the evaluation metrics of the pre‐trained model cover both classification and regression tasks. The model performance is evaluated through distinct metric frameworks corresponding to different prediction tasks: classification metrics (accuracy, recall, F1‐score) for discrete categorical predictions, while regression metrics like mean squared error [MSE], mean absolute error [MAE) and Pearson correlation coefficient are used to evaluate the Ribo‐seq signal prediction. These quantitative measures are formally defined as follows:

(2) $$MSE=\frac{1}{N}\;\sum_{i=1}^{N}{\left(y_{true}-y_{pred}\right)}^{2}$$

(3) $$MAE=\frac{1}{N}\;\sum_{i=1}^{N}\left|y_{true}-y_{pred}\right|$$

where *N* represents number of samples, *y_true_* represents the true value of the i‐th sample and *y_pred_* represents the predicted value the i‐th sample.

Additionally, for the fine‐tuned model, performance is mainly evaluated using ROC‐AUC and PR AUC:

(4) $$\begin{array}{c} TPR=\frac{TP}{TP+FN}\\ FPR=\frac{FP}{FP+TN}\\ AUC=\int_{0}^{1}TPR\left(T\right)\frac{\partial FPR\left(t\right)}{\partial T}dt \end{array}$$

(5) $$PR\;AUC\int_{0}^{1}Precision\left(Recall\right)dRecall$$

### MS Analysis of Short Peptides

The sequences of candidate ORFs were translated into amino acid sequences. Mass spectrometry (MS) was then used to identify translated amino acid sequences using MaxQuant^[^ ^52^ ^]^ and FragPipe.^[^ ^53^ ^]^ The MS datasets were derived from Q‐Exactive instruments and obtained from the ProteomeXchange database.^[^ ^54^ ^]^ MaxQuant is a robust and efficient proteomics software package for analyzing large‐scale MS datasets utilizing the Andromeda^[^ ^55^ ^]^ search algorithm. FragPipe is an ultra‐fast proteomics analysis pipeline powered by MSFragger. It supports direct (“library‐free”) peptide identification through workflows such as MSFragger‐DIA,^[^ ^56^ ^]^ DIA‐Umpire/MSFragger,^[^ ^57^ ^]^ and diaTracer/MSFragger,^[^ ^58^ ^]^ and facilitates quantitative data extraction using DIA‐NN and Skyline. The results of the MS analysis enhanced the confidence in the predicted sORFs. The parameters used for MaxQuant and FragPipe are provided in Table S6 (Supporting Information).

Based on the length criteria for sORFs, the modified RibORF prediction results were filtered to identify candidate sORFs shorter than 180 nt with ORF scores greater than 0. The amino acid sequences of these candidates were then subjected to MS analysis using MaxQuant and FragPipe. The resulting peptide identifications were cross‐referenced with the predicted sORFs to validate their translational evidence. Additionally, sORFs predicted by sORFPredRibo were also cross‐validated against peptides identified by FragPipe to further identify high‐confidence sORFs supported by MS evidence.

### DiffTE Analysis

To assess differences in translational efficiency (TE) across conditions, the he trimmed mean of M values (TMM) normalization method was applied and implemented in edgeR^[^ ^59^ ^]^ to independently normalize the raw count data from Ribo‐seq and RNA‐seq experiments. Following normalization, the corresponding Reads Per Kilobase of transcript per Million mapped reads (RPKM) was calculated. TE was defined as the ratio of Ribo‐seq RPKM to RNA‐seq RPKM for each gene. Then log_2_‐transformed the TE values and performed differential analysis using the limma^[^ ^60^ ^]^ package, which enables linear modeling and empirical Bayes statistical testing, to identify differentially translated genes. For comparison, the same dataset using DESeq2,^[^ ^61^ ^]^ a method was also analyzed that models count data using a negative binomial distribution and serves as the core algorithm in DeltaTE. Under the same thresholds |log2FC| ≥ 1.5 and unadjusted p‐value < 0.05), DESeq2 identified fewer differentially translated genes than limma (Figure S11, Supporting Information). This discrepancy may be attributed to two main factors: 1) in prokaryotic organisms, transcription and translation are highly coupled and lack complex splicing events, making RPKM‐based TE calculation both reasonable and commonly used in prokaryotic translation regulation studies; and 2) the relatively small number of biological replicates in our study (n = 2–3) may limit statistical power. Under such low‐replicate conditions, DESeq2 tends to be more conservative due to its negative binomial modeling framework, potentially reducing sensitivity. In contrast, limma, with its empirical Bayes method, offers greater stability and power for small‐sample data. Based on these considerations, limma was selected as the primary method for differential TE analysis in this study.

### DiffCO Analysis

DiffCO was designed to investigate differences in codon preference across experimental conditions. Custom scripts in R was developed to perform codon occupancy analysis on Ribo‐seq data, aiming to assess ribosome residence frequencies on individual codons during translation. First, triplet‐phase filtering was applied to the Ribo‐seq reads mapped to the CDS region and removed the first and last 15 codons to avoid edge effects. It then extracted ribosome footprint coverage at the A, P, and E sites, as well as at the A‐site codon located downstream (+1 to +3), and normalized these signals by the average footprint coverage of the downstream region to control for differences in gene expression levels. Triplet sequences were subsequently translated into amino acids, and normalized occupancy values were aggregated for each codon. Codon‐specific ribosome enrichment patterns under different conditions were visualized using the ggplot2 package. For differential analysis, a linear model using the limma package and computed statistical significance (*p*‐values) was constructed via its empirical Bayes framework. Codons showing significant differential occupancy were identified based on a threshold of |log_2_ fold change| ≥ 1.5 and *p* < 0.05.

### Reported sORF and Related Annotation Data Sets

The 834 reported were collected sORFs manually from the peer‐reviewed literature, predominantly for bacteria, such as Bacillus subtilis, Escherichia coli, Salmonella enterica, Listeria monocytogenes, Yersinia pestis, and Lacticaseibacillus rhamnosus GG. RNA modifications including m^1^A, m^6^A, m^7^G, m^5^C, and pseudouridine were integrated from DirectRMDB^[^ ^62^ ^]^ and RMbase v3.0.^[^ ^63^ ^]^ Riboswitch datasets were obtained from the Ribocentre‐switch database.^[^ ^64^ ^]^

### Construction of the Database and Web Interface

The website was developed based on hypertext preprocessor (PHP) and hypertext markup language (HTML) using the Bootstrap framework. JQuery, DataTable, and Highchart JavaScript libraries were used for dynamic and interactive visualization of the data stored in the MySQL database. The data analysis tools in the database were developed using Python/Perl and R scripts. The whole database is hosted on a Linux system equipped with 1024 GB of RAM and 16 octa‐core AMD processors (2.6 GHz each). Additionally, JBrowse, a web‐based genome visualization browser, was integrated for visualizing genes, modification sites, and riboswitches^[^ ^65^ ^]^ and mRNAbrowse was designed to visualize candidate ORFs and translated ORFs based on igv.js (https://github.com/igvteam/igv.js).^[^ ^66^ ^]^

## Conflict of Interest

The authors declare no conflict of interest.

## Author Contributions

Y.Z., J.L., and X.L. contributed equally to this work and are co‐first authors. L.Q., C.Y., L.J., and Z.Y. performed Conceptualization; L.Q., L.J., and L.X. performed methodology; L.J., L.X., and L.G. performed formal analysis; G.Y., L.F., C.Y., D.Y., C.B., W.Y., and Z.W. performed investigation; L.J., L.X., and L.G. performed visualization and database; L.J., L.Q., and L.X. wrote–original draft; L.J., L.Q., and L.X. wrote–review and edited; Z.Y., C.Y., and L.Q. performed supervision.

## Supporting information



Supplemental Figures S1–S11

Supplemental Table S1–S6

## Data Availability

The data that support the findings of this study are available in the supplementary material of this article.
