# Supplementary material for: RiboMicrobe: An Integrated Translatome Atlas for Microorganism
Source: Adv Sci (Weinh). 2025 Oct 13;12(48):e09877. doi: 10.1002/advs.202509877 (PMC12752654; doi:10.1002/advs.202509877)
Supplement: Supplementary file 1 — Supplemental Figures S1–S11 [file ADVS-12-e09877-s001.zip › re_Figure S11.pdf]

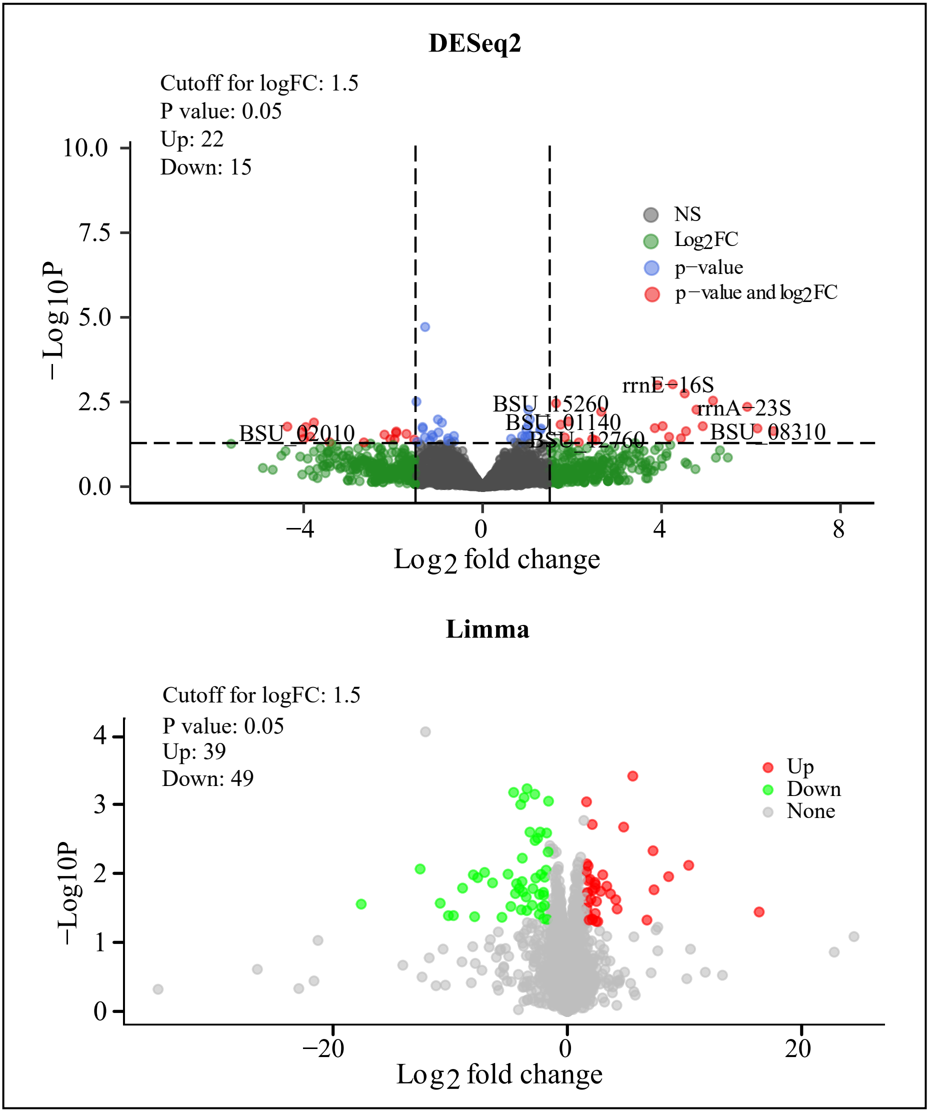

Figure S11. Comparison of differential analysis results between DESeq2 and limma under consistent threshold criteria.
