# Supplementary material for: RiboMicrobe: An Integrated Translatome Atlas for Microorganism
Source: Adv Sci (Weinh). 2025 Oct 13;12(48):e09877. doi: 10.1002/advs.202509877 (PMC12752654; doi:10.1002/advs.202509877)
Supplement: Supplementary file 1 — Supplemental Figures S1–S11 [file ADVS-12-e09877-s001.zip › Figure S3.pdf]

A

B

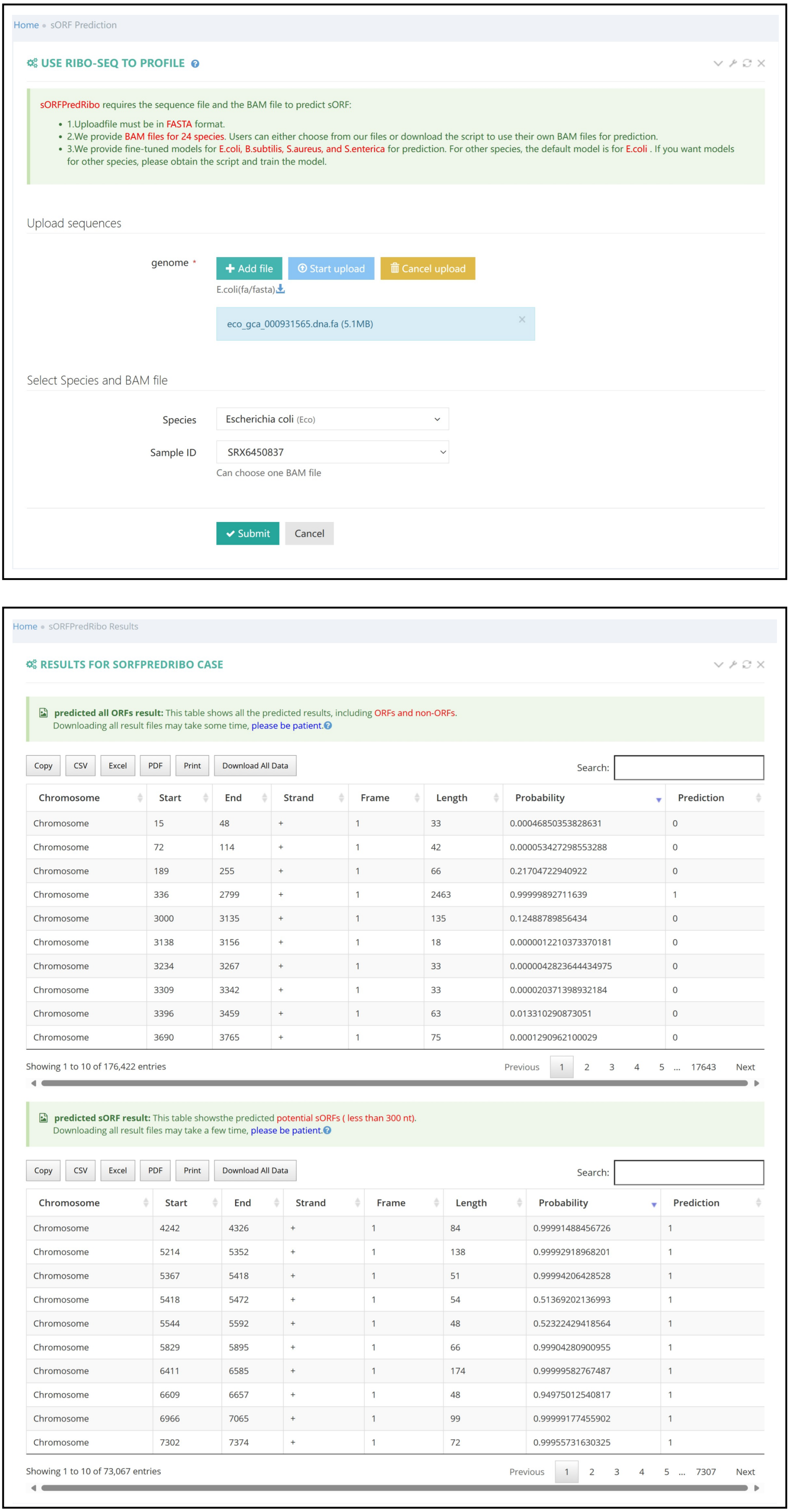

Home • sORF Prediction

USE RIBO-SEQ TO PROFILE

sORFPredRibo requires the sequence file and the BAM file to predict sORF:

- 1.Uploadfile must be in FASTA format.
- 2.We provide BAM files for 24 species. Users can either choose from our files or download the script to use their own BAM files for prediction.
- 3.We provide fine-tuned models for E.coli, B.subtilis, S.aureus, and S.enterica for prediction. For other species, the default model is for E.coli . If you want models for other species, please obtain the script and train the model.

Upload sequences

genome * Add file Start upload Cancel upload

E.coli(fa/fasta)

eco_gca_000931565.dna.fa (5.1MB)

Select Species and BAM file

Species: Escherichia coli (Eco)

Sample ID: SRX6450837

Can choose one BAM file

Submit Cancel

Home • sORFPredRibo Results

RESULTS FOR SORFPREDRIBO CASE

predicted all ORFs result: This table shows all the predicted results, including ORFs and non-ORFs.
Downloading all result files may take some time, please be patient.

Copy CSV Excel PDF Print Download All Data Search:

Chromosome	Start	End	Strand	Frame	Length	Probability	Prediction
Chromosome	15	48	+	1	33	0.00046850353828631	0
Chromosome	72	114	+	1	42	0.000053427298553288	0
Chromosome	189	255	+	1	66	0.21704722940922	0
Chromosome	336	2799	+	1	2463	0.99999892711639	1
Chromosome	3000	3135	+	1	135	0.12488789856434	0
Chromosome	3138	3156	+	1	18	0.0000012210373370181	0
Chromosome	3234	3267	+	1	33	0.0000042823644434975	0
Chromosome	3309	3342	+	1	33	0.000020371398932184	0
Chromosome	3396	3459	+	1	63	0.013310290873051	0
Chromosome	3690	3765	+	1	75	0.0001290962100029	0

Showing 1 to 10 of 176,422 entries Previous 1 2 3 4 5 … 17643 Next

predicted sORF result: This table showsthe predicted potential sORFs (less than 300 nt).
Downloading all result files may take a few time, please be patient.

Copy CSV Excel PDF Print Download All Data Search:

Chromosome	Start	End	Strand	Frame	Length	Probability	Prediction
Chromosome	4242	4326	+	1	84	0.99991488456726	1
Chromosome	5214	5352	+	1	138	0.99992918968201	1
Chromosome	5367	5418	+	1	51	0.99994206428528	1
Chromosome	5418	5472	+	1	54	0.51369202136993	1
Chromosome	5544	5592	+	1	48	0.52322429418564	1
Chromosome	5829	5895	+	1	66	0.99904280900955	1
Chromosome	6411	6585	+	1	174	0.99999582767487	1
Chromosome	6609	6657	+	1	48	0.94975012540817	1
Chromosome	6966	7065	+	1	99	0.99999177455902	1
Chromosome	7302	7374	+	1	72	0.99955731630325	1

Showing 1 to 10 of 73,067 entries Previous 1 2 3 4 5 … 7307 Next

Figure S3. Visualization of sORFPredRibo in RiboMicrobe. (A) Parameter selection. (B) The prediction results.
