# Supplementary material for: RiboMicrobe: An Integrated Translatome Atlas for Microorganism
Source: Adv Sci (Weinh). 2025 Oct 13;12(48):e09877. doi: 10.1002/advs.202509877 (PMC12752654; doi:10.1002/advs.202509877)
Supplement: Supplementary file 1 — Supplemental Figures S1–S11 [file ADVS-12-e09877-s001.zip › Figure S4.pdf]

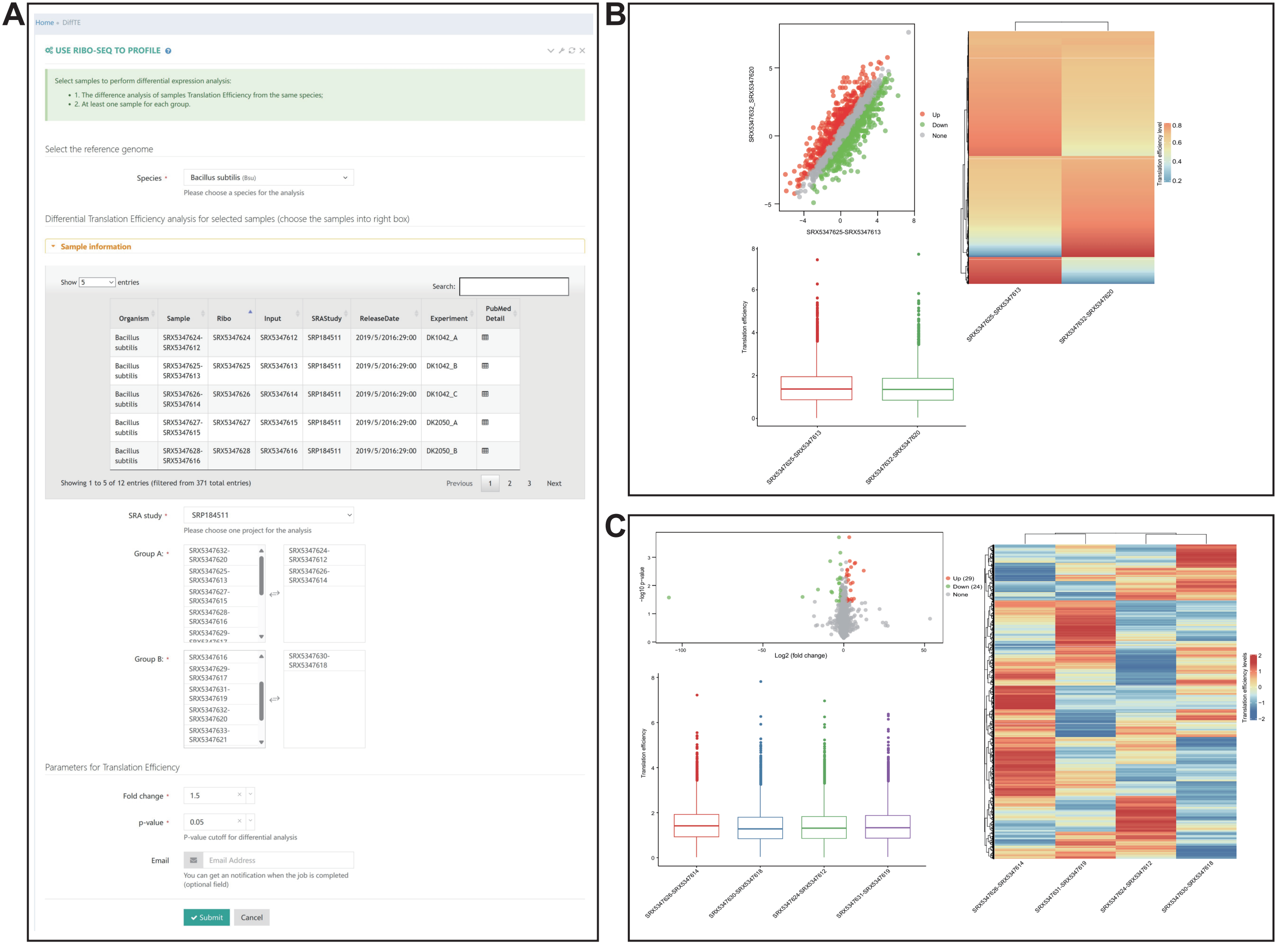

Figure S4. Visualization of DiffTE in RiboMicrobe. (A)Parameter selection. (B) Two groups of samples for a comparative analysis; each group contains a pair of corresponding samples. Results are shown as a scatter plot, heatmap, and boxplot. (C) Each group contains multiple pairs of corresponding samples. Results are shown as a volcano plot, heatmap, and boxplot.
