# Supplementary material for: RiboMicrobe: An Integrated Translatome Atlas for Microorganism
Source: Adv Sci (Weinh). 2025 Oct 13;12(48):e09877. doi: 10.1002/advs.202509877 (PMC12752654; doi:10.1002/advs.202509877)
Supplement: Supplementary file 1 — Supplemental Figures S1–S11 [file ADVS-12-e09877-s001.zip › Figure S5.pdf]

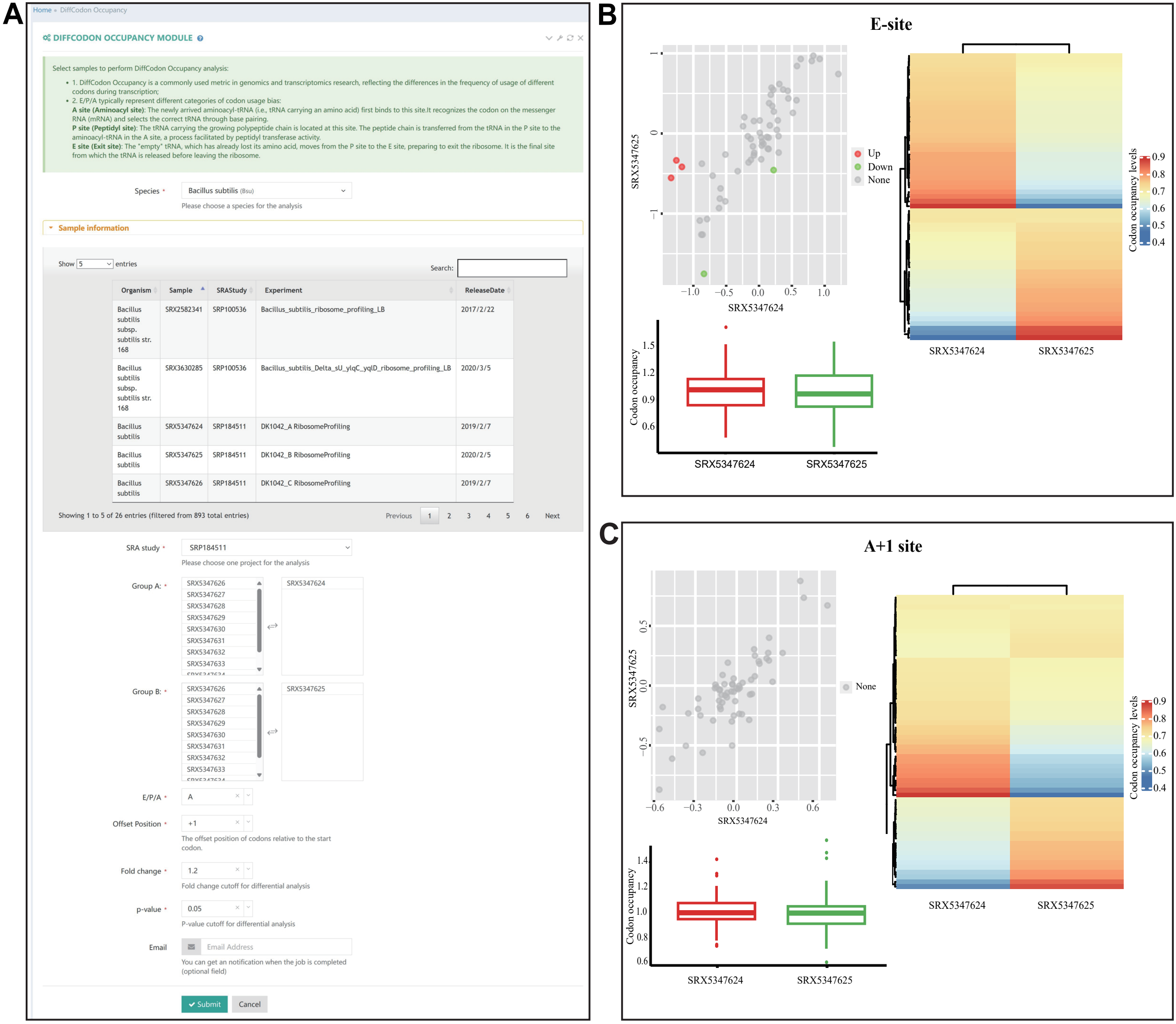

Figure S5. Visualization of the result for DiffCO between the sample SRX5347624 and SRX5347625. （A） Parameter selection. (B–C) Scatter plot, Heatmap and Boxplot of the E-site and A+1 site.
