# Supplementary figures and images for: RiboMicrobe: An Integrated Translatome Atlas for Microorganism
Source: Adv Sci (Weinh). 2025 Oct 13;12(48):e09877. doi: 10.1002/advs.202509877 (PMC12752654; doi:10.1002/advs.202509877)

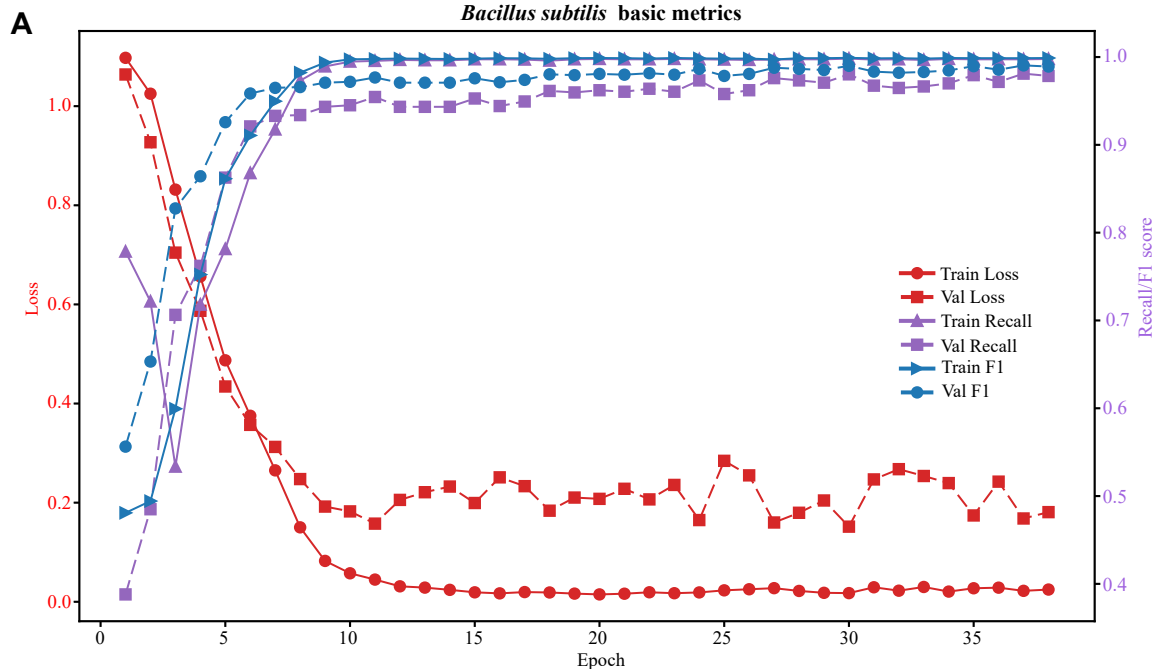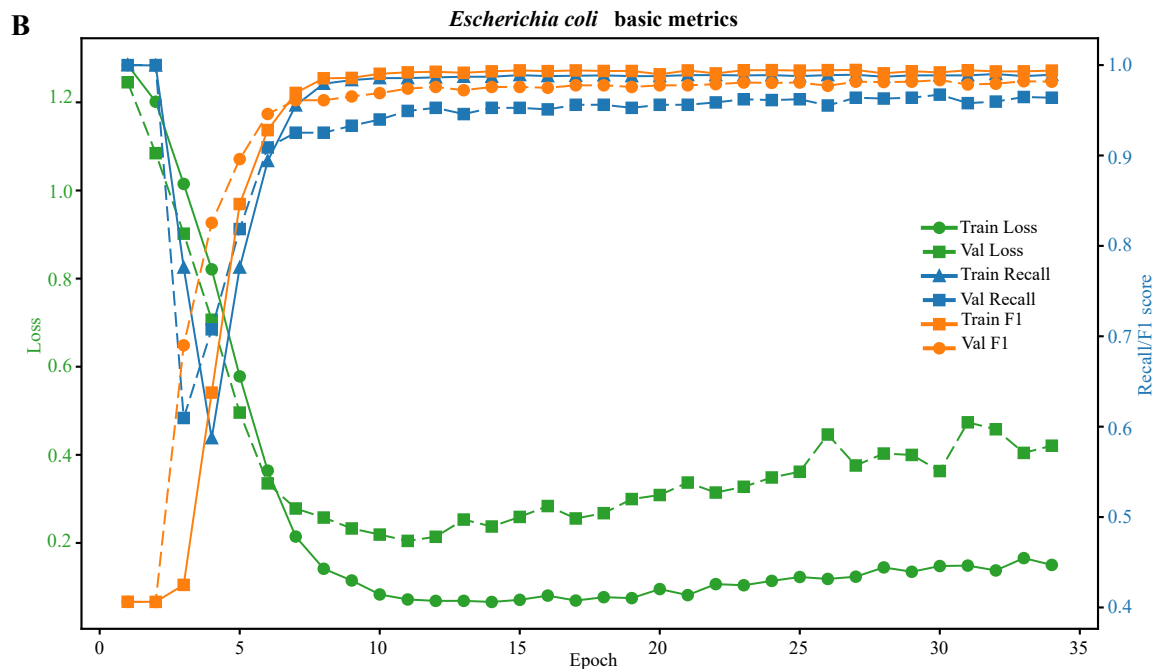

**Figure S8.** Basic performance evaluation of sORFPredRibo.

Supplement: Supplementary file 1 — Supplemental Figures S1–S11 [file ADVS-12-e09877-s001.zip › re_Figure S8.pdf]

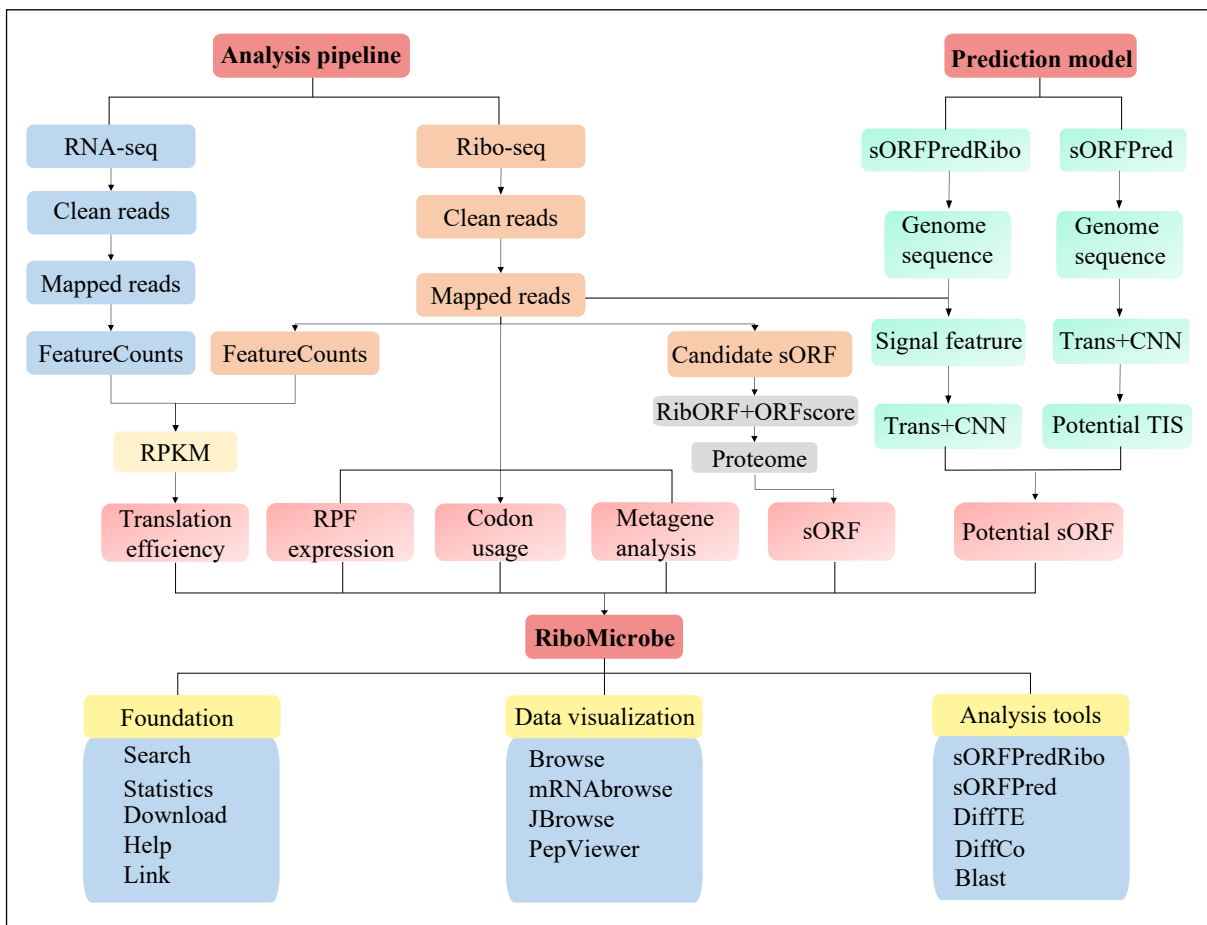

**Figure S10.** Analysis pipeline of RiboMicrobe

Supplement: Supplementary file 1 — Supplemental Figures S1–S11 [file ADVS-12-e09877-s001.zip › re_Figure S10.pdf]
