# Supplementary material for: RiboMicrobe: An Integrated Translatome Atlas for Microorganism
Source: Adv Sci (Weinh). 2025 Oct 13;12(48):e09877. doi: 10.1002/advs.202509877 (PMC12752654; doi:10.1002/advs.202509877)
Supplement: Supplementary file 2 — Supplemental Table S1–S6 [file ADVS-12-e09877-s002.zip › re_Table S5.docx]

**Table S5. Classification and Functions of Tools.**

| Classification | Name | Function | |
| --- | --- | --- | --- |
| In-house Tools | sORFPred | Prediction of TIS and sORFs based solely on sequence features. | |
|  | sORFPredRibo | Prediction of sORFs integrating both sequence features and Ribo-seq signal features. | |
|  | DiffCO | Comparative analysis of codon usage preferences across samples under varying experimental conditions | |
| Third-party Tools | BLAST | Sequence alignment for the identification of potential sORFs | |
|  | DeepRibo | ORF prediction utilizing a hybrid CNN--RNN framework. | |
| Adapted Tools | RibORF2.0 | Evaluate the translational potential of ORFs using a logistic regression model | |
|  | DiffTE | | Comparative assessment of translational efficiency across samples subjected to different conditions. |
