# Supplementary material for: RiboMicrobe: An Integrated Translatome Atlas for Microorganism
Source: Adv Sci (Weinh). 2025 Oct 13;12(48):e09877. doi: 10.1002/advs.202509877 (PMC12752654; doi:10.1002/advs.202509877)
Supplement: Supplementary file 2 — Supplemental Table S1–S6 [file ADVS-12-e09877-s002.zip › re_Table S6.docx]

**Table S6. Parameters used for mass spectrometry analysis in MaxQuant and FragPipe**

MaxQuant (version 2.0.3.0) was used for mass spectrometry data analysis with the following key parameters:

| parameters | value |
| --- | --- |
| Enzyme specificity | Trypsin/P, allowing up to two missed cleavages |
| Fixed modification | Carbamidomethylation of cysteine |
| Variable modifications | Oxidation of methionine and acetylation at the protein N-terminus |
| Quantification | Label-Free Quantification enabled |
| Minimum peptide length | 7 amino acids |
| Minimum number of peptides required for protein identification | at least 1 razor peptide |
| Match between runs | disabled |
| Second peptide search | enabled |
| False Discovery Rate (FDR) | controlled at 1% for peptides, proteins, and modification sites |
| Mass tolerance | Orbitrap default settings |

FragPipe (version 17.1) with MSFragger was also used for spectral matching analysis with the following key parameters:

| parameters | value |
| --- | --- |
| Enzyme specificity | strict trypsin, allowing up to two missed cleavages |
| Precursor mass tolerance | ±20 ppm |
| Fragment mass tolerance | ±20 ppm |
| Data acquisition type | Data-Dependent Acquisition (DDA) |
| Fixed modification | Carbamidomethylation of cysteine (+57.02146 Da) |
| Variable modifications | Oxidation of methionine (+15.9949 Da) and N-terminal acetylation of proteins (+42.0106 Da) |
| Minimum peptide length | 7 amino acids |
| Maximum number of variable modifications per peptide | 3 |
| Maximum number of modification combinations | 5000 |
| Output formats | pepXML and pin files |
