# Supplementary material for: RiboMicrobe: An Integrated Translatome Atlas for Microorganism
Source: Adv Sci (Weinh). 2025 Oct 13;12(48):e09877. doi: 10.1002/advs.202509877 (PMC12752654; doi:10.1002/advs.202509877)
Supplement: Supplementary file 2 — Supplemental Table S1–S6 [file ADVS-12-e09877-s002.zip › Table S1.docx]

### Table S1. The information of species and datasets in RiboMicrobe

| **Species** | **Reference genomes scourse** | **Reference genomes version** | **Ribo-seq data** | **Matched RNA-seq data** | **Proteome dataset** | **RNA- Modification sites** | **Riboswith** |
| --- | --- | --- | --- | --- | --- | --- | --- |
| *Acetobacterium woodii* | EnsemblBacteria | ASM24760v1 | 4 | 4 | 0 | 0 | 26 |
| *Bacillus subtilis* | EnsemblBacteria | ASM904v1 | 26 | 12 | 10 | 129 | 21 |
| *Bacteroides thetaiotaomicron* | EnsemblBacteria | ASM1106v1 | 2 | 2 | 3 | 0 | 0 |
| *Caulobacter vibrioides* | EnsemblBacteria | ASM2200v1 | 11 | 8 | 2 | 0 | 0 |
| *Clostridium aceticum* | EnsemblBacteria | ASM104271v1 | 8 | 4 | 0 | 0 | 40 |
| *Clostridium drakei* | EnsemblBacteria | ASM309617v1 | 4 | 0 | 0 | 0 | 0 |
| *Clostridium ljungdahlii* | EnsemblBacteria | ASM14368v1 | 6 | 4 | 0 | 0 | 0 |
| *Escherichia coli* | EnsemblBacteria | ASM93156v1 | 498 | 159 | 9 | 798 | 5 |
| *Eubacterium limosum* | EnsemblBacteria | ASM148172v1 | 4 | 4 | 4 | 0 | 0 |
| *Flavobacterium johnsoniae* | EnsemblBacteria | ASM1664v1 | 3 | 3 | 2 | 0 | 3 |
| *Halobacterium salinarum* | EnsemblBacteria | ASM680v1 | 11 | 0 | 2 | 72 | 0 |
| *Haloferax volcanii* | EnsemblBacteria | ASM2568v1 | 11 | 3 | 3 | 355 | 0 |
| *Human betaherpesvirus* | RefSeq | ViralProj14559 | 3 | 0 | 2 | 0 | 0 |
| *Klebsiella michiganensis* | EnsemblBacteria | ASM96357v1 | 8 | 7 | 0 | 0 | 0 |
| *Lacticaseibacillus rhamnosus* | EnsemblBacteria | ASM228794v1 | 3 | 3 | 1 | 0 | 0 |
| *Listeria innocua* | EnsemblBacteria | ASM19579v1 | 2 | 0 | 0 | 0 | 0 |
| *Listeria monocytogenes* | EnsemblBacteria | ASM19603v1 | 2 | 0 | 3 | 0 | 20 |
| *Mycobacterium tuberculosis* | EnsemblBacteria | ASM1614v1 | 20 | 0 | 4 | 0 | 6 |
| *Mycobacteroides abscessus* | EnsemblBacteria | ASM6918v1 | 2 | 2 | 1 | 0 | 0 |
| *Mycolicibacterium smegmatis* | EnsemblBacteria | ASM1500v1 | 9 | 2 | 3 | 4 | 0 |
| *Pseudomonas aeruginosa* | EnsemblBacteria | ASM296875v1 | 20 | 20 | 4 | 904 | 2 |
| *Pseudomonas fluorescens* | EnsemblBacteria | ASM130715v1 | 4 | 0 | 2 | 0 | 4 |
| *Salmonella enterica* | EnsemblBacteria | ASM332505v1 | 29 | 18 | 2 | 0 | 0 |
| *Sinorhizobium meliloti* | EnsemblBacteria | ASM219712v1 | 2 | 2 | 1 | 0 | 0 |
| *Staphylococcus aureus* | EnsemblBacteria | ASM101865v2 | 24 | 14 | 2 | 0 | 13 |
| *Streptococcus pneumoniae* | EnsemblBacteria | ASM1896v1 | 4 | 0 | 0 | 0 | 18 |
| *Streptomyces avermitilis* | EnsemblBacteria | ASM976v2 | 31 | 23 | 0 | 0 | 18 |
| *Streptomyces clavuligerus* | RefSeq | ASM551946v1 | 28 | 24 | 0 | 0 | 15 |
| *Streptomyces coelicolor* | RefSeq | ASM20383v1 | 4 | 0 | 1 | 2 | 13 |
| *Streptomyces griseus* | EnsemblBacteria | ASM1060v1 | 8 | 0 | 0 | 0 | 15 |
| *Streptomyces lividans* | RefSeq | ASM73910v1 | 8 | 8 | 3 | 0 | 0 |
| *Streptomyces tsukubensis* | RefSeq | ASM393271v1 | 24 | 24 | 0 | 0 | 0 |
| *Streptomyces venezuelae* | EnsemblBacteria | ASM25323v1 | 16 | 0 | 1 | 0 | 16 |
| *Synechocystis* | EnsemblBacteria | ASM47882v2 | 22 | 16 | 4 | 0 | 0 |
| *Vaccinia virus* | RefSeq | GCA900236015 | 8 | 0 | 2 | 0 | 0 |
| *Vibrio natriegens* | EnsemblBacteria | ASM976v2 | 1 | 0 | 1 | 0 | 0 |
| *Vibrio vulnificus* | EnsemblBacteria | ASM3976v1 | 3 | 3 | 1 | 0 | 0 |
| *Zymomonas mobilis* | EnsemblBacteria | ASM710v1 | 18 | 0 | 2 | 0 | 0 |
