# Supplementary material for: RiboMicrobe: An Integrated Translatome Atlas for Microorganism
Source: Adv Sci (Weinh). 2025 Oct 13;12(48):e09877. doi: 10.1002/advs.202509877 (PMC12752654; doi:10.1002/advs.202509877)
Supplement: Supplementary file 2 — Supplemental Table S1–S6 [file ADVS-12-e09877-s002.zip › Table S2.docx]

**Table S2. Experimentally reported micropeptides in RiboMicrobe**

| **Species** | **ID** | **Chromosome** | **Sequence** | **Length** | **Location** | **PMID** |
| --- | --- | --- | --- | --- | --- | --- |
| *Acetobacterium woodii* | Awo_c34260 | CP002987.1 | MPPDPARGTDVHNGLGLWFAAQVVKAHAGQLELQNRRRFYPLLTNTEPF | 49 | CP002987.1:3917255-3917404 | 22479398 |
| *Acetobacterium woodii* | WP_083837879 | - | MDIKGLRYFLSAAENLSFTKAAIEQKVTQTAISLSIGKMEDELGFKLFF | 49 | - | 9309218 |
| *Acetobacterium woodii* | Awo_c34470 | CP002987.1 | MTYSYCKTVIKNGRYGTKEAMMVKLDVFLLNDRITQEEYTELVELLNAAA | 50 | CP002987.1:3977739-3977891 | 22479398 |
| *Acetobacterium woodii* | Awo_c33820 | CP002987.1 | MADSSMNGSPIRYKIVCENCTQSNPCSMFFNFAIAYDTDKSKHVLI | 46 | CP002987.1:3873475-3873615 | 22479398 |
| *Bacillus subtilis* | BSU_22036 | OZ024942.3 | MTMLFLLGAVCTYSVLIGFVLKGISNKSV | 29 | OZ024942.3:2316286-2316375 | 11918817 |
| *Bacillus subtilis* | BSUB_00496 | CP008698.1 | MTPQSAEIRKKQLIHLLIQNGIYKKSKRHLYELSLTELESEYKHVRRAPEHAEA | 54 | CP008698.1:490001-490165 | 24994804 |
| *Bacillus subtilis* | BSUB_00497 | OZ024942.3 | MKVHRMPKGVVLVGKAWEIRAKLKEYGRTFQYVKDWISKP | 40 | OZ024942.3:2459141-2459263 | 29280348 |
| *Bacillus subtilis* | WP_352230668 | - | MKRLSDKDLIEAYMSAKKLLDIDPLFIKQLETELKRRSISIENKQTNNR | 49 | - | 15023339 |
| *Bacillus subtilis* | BSU06839 | - | MPIKKKVMMCLAVTLVFGSMSFPTLTNSGGFKESTDRNTTYIDHSPYKLSDQKKALS | 57 | - | 9384377 |
| *Bacillus subtilis* | AAC_36809 | L12244.1 | MKFYTIKLPKFLGGIVRAMLGSFRKD | 26 | L12244.1:144-224 | 8231808 |
| *Bacillus subtilis* | BSU_21546 | - | MTVYESLMIMINFGGLILNTVLLIFNIMMIVTSSQKKK | 38 | - | [20525796](https://www.ncbi.nlm.nih.gov/pubmed/20525796) |
| *Bacillus subtilis* | BSU_03500 | OZ024942.3 | MNRSGKHLISSIILYPRPSGECISSISLDKQTQATTSPLYFCWREK | 46 | OZ024942.3:390880-391020 | [9384377](https://www.ncbi.nlm.nih.gov/pubmed/9384377) |
| *Bacillus subtilis* | BSU_03780 | OZ024942.3 | MKLKSKLFVICLAAAAIFTAAGVSANAEALDFHVTERGMT | 40 | OZ024942.3:429963-430085 | 9384377 |
| *Bacillus subtilis* | BSU_03788 | OZ024942.3 | MSGYGTSFALIVVLFILLIIVGTAFVGGY | 29 | OZ024942.3:430185-430274 | 9384377 |
| *Bacillus subtilis* | BSU_03789 | OZ024942.3 | MSGYSNGGGYGGISSFALIVVLFILLIIVGTAFVGGF | 37 | OZ024942.3:430356-430469 | 9384377 |
| *Bacillus subtilis* | BSU_04785 | OZ024942.3 | MPNWLKKQMQKAFLEKDNYQIKLLNQCWYFYRKKHCS | 37 | OZ024942.3:527912-528025 | 9384377 |
| *Bacillus subtilis* | BSU_05020 | OZ024942.3 | MKISRILLAAVILSSVFSITYLQSDHNTEIKVAADRVGA | 39 | OZ024942.3:548438-548557 | 9384377 |
| *Bacillus subtilis* | BSU_08550 | OZ024942.3 | MVRNKEKGFPYENENKFQGEPRAKDDYASKRADGSINQHPQERMRASGKR | 50 | OZ024942.3:928112-928264 | 10806362 |
| *Bacillus subtilis* | BSU_09958 | OZ024942.3 | MSGGYSNGFALLVVLFILLIIVGAAYIY | 28 | OZ024942.3:1071402-1071488 | 20156992 |
| *Bacillus subtilis* | BSU_09959 | OZ024942.3 | MGEVFAGGFALLVVLFILLIIIGASWLY | 28 | OZ024942.3:1071613-1071699 | 21670523 |
| *Bacillus subtilis* | BSU_08500 | OZ024942.3 | MEKKREKHQQGANLKKMQEVLYSGEFKKAEKAAKRK | 36 | OZ024942.3:924468-924578 | 15699190 |
| *Bacillus subtilis* | BSU_11809 | OZ024942.3 | MGFGYGFGGGYGGGCYGGYAGGYGGGYGSTFVLLVVLFILLIIVGASFF | 49 | OZ024942.3:1253103-1253252 | 30793535 |
| *Bacillus subtilis* | BSU_12440 | OZ024942.3 | MKSKWMSGLLLVAVGFSFTQVMVHAGETANTEGKTFHIAARNQT | 44 | OZ024942.3:1316995-1317129 | 1378051 |
| *Bacillus subtilis* | BSU_12672 | OZ024942.3 | MYYAMHELHYSPSQLLELYEAPKHFKALLFGLIGYKLDLLEKESRRGGN | 49 | OZ024942.3:1334815-1334964 | 15469818 |
| *Bacillus subtilis* | BSU_12815 | OZ024942.3 | MSGDTVKKLVRRVKFVDYGRFGLSGYSLRVRERSAGIIKKLKKKK | 45 | OZ024942.3:1348219-1348356 | 11371520 |
| *Bacillus subtilis* | BSU_13290 | OZ024942.3 | MEKKEEQYINQAEYVPHPTKEGEYALFLHETYHLLSEDDETQTTE | 45 | OZ024942.3:1395371-1395508 | 10629212 |
| *Bacillus subtilis* | BSU_14568 | OZ024942.3 | MPTYKGSGVIKRGGYVICMDMVTAEAAAVMVVTDMVAADMATAAPLR | 47 | OZ024942.3:1526924-1527067 | 9384377 |
| *Bacillus subtilis* | BSU_14629 | OZ024942.3 | MGTIVCQDCNEAIHYFEDEKVTTLYGTCCGQCQCPVDEE | 39 | OZ024942.3:1534120-1534239 | 16164558 |
| *Bacillus subtilis* | BSU_15810 | OZ024942.3 | MKFYTIKLPKFLGGIVRAMLGSFRKD | 26 | OZ024942.3:1655446-1655526 | 9922240 |
| *Bacillus subtilis* | BSU_18978 | OZ024942.3 | MSTFQALMLMLAIGSFIIALLTYIEKIDLP | 30 | OZ024942.3:2069883-2069975 | 26565032 |
| *Bacillus subtilis* | BSU_19920 | OZ024942.3 | MDSREQIDWTCNECNFSWIGDNSDFSCPSCDEIDIKPKNKILD | 43 | OZ024942.3:2154887-2155018 | 20233928 |
| *Bacillus subtilis* | BSU_21546 | OZ024942.3 | MTVYESLMIMINFGGLILNTVLLIFNIMMIVTSSQKKK | 38 | OZ024942.3:2273594-2273710 | 22229825 |
| *Bacillus subtilis* | BSU_23616 | OZ024942.3 | MKVHRMPKGVVLVGKAWEIRAKLKEYGRTFQYVKDWISKP | 40 | OZ024942.3:2459141-2459263 | 18284588 |
| *Bacillus subtilis* | BSU_25840 | OZ024942.3 | MKSKLFISLSAVLIGLAFFGSMYNGEMKEASRNVTLAPTHEFLV | 44 | OZ024942.3:2660330-2660464 | 10629174 |
| *Bacillus subtilis* | BSU_36430 | OZ024942.3 | MGIFHKLTFKTIQRRSGIMNDSLQNTDLISHFSHPF | 36 | OZ024942.3:3748717-3748827 | 9023218 |
| *Bacillus subtilis* | BSU_37350 | OZ024942.3 | MKKAVIVENKGCATCSIGAACLVDGPIPDFEIAGATGLFGLWG | 43 | OZ024942.3:3836058-3836189 | 10572140 |
| *Escherichia coli* | SPROECO16928 | MG1655 | MRMLT | 5 | MG1655:2725939-2725956 | 8648637 |
| *Escherichia coli* | SPROECO16951 | MG1655 | MRTG-N | 7 | MG1655:4350229-4350252 | 2861788 |
| *Escherichia coli* | SPROECO16482 | NC_000913.3 | MIKSDQET | 8 | NC_000913.3:3737177-3737203 | 30837344 |
| *Escherichia coli* | SPROECO16238 | NC_000913.3 | MAHIASSIT | 9 | NC_000913.3:---:- | 28245801 |
| *Escherichia coli* | SPROECO16393 | NC_000913.3 | MFHDLPGVK | 9 | NC_000913.3:3573679-3573708 | 30837344 |
| *Escherichia coli* | SPROECO16675 | NC_000913.3 | MLHCKGNNL | 9 | NC_000913.3:2483729-2483758 | 30837344 |
| *Escherichia coli* | SPROECO17157 | NC_000913.3 | MVDKFNNSDC | 10 | NC_000913.3:754658-754690 | 30837344 |
| *Escherichia coli* | SPROECO17202 | U00096.3 | - | 10 | U00096.3:263025-263057 | 30904393 |
| *Escherichia coli* | SPROECO17069 | NC_000913.3 | MSVSCWEF-G | 11 | NC_000913.3:2347350-2347385 | 30837344 |
| *Escherichia coli* | SPROECO16507 | NC_000913.3 | MIYRELNRALAIL | 13 | NC_000913.3:3573660-3573701 | 30837344 |
| *Escherichia coli* | SPROECO16803 | NC_000913.3 | MNREEMHCDVVKI | 13 | NC_000913.3:2722702-2722743 | 30837344 |
| *Escherichia coli* | SPROECO17168 | NC_000913.3 | MVQLVDLARCVSF | 13 | NC_000913.3:1567178-1567219 | 29645342 |
| *Escherichia coli* | SPROECO16400 | NC_000913.3 | MFSKLAQSSIKAMF | 14 | NC_000913.3:1212260-1212304 | 29645342 |
| *Escherichia coli* | SPROECO16475 | NC_000913.3 | MIIKTLKMSARKRN | 14 | NC_000913.3:113244-113288 | 29645342 |
| *Escherichia coli* | SPROECO16509 | MG1655 | MKAIFVLKGWWRTS | 14 | MG1655:1323038-1323082 | 16397293 |
| *Escherichia coli* | SPROECO16742 | MG1655 | M-AIFRFFFYFST | 14 | MG1655:1799226-1799270 | 16397293 |
| *Escherichia coli* | SPROECO16967 | MG1655 | MSAVLLRRLISPSK | 14 | MG1655:52444-52488 | 3122846 |
| *Escherichia coli* | SPROECO17000 | NC_000913.3 | MSIFRIHLDGNKKA | 14 | NC_000913.3:1961837-1961881 | 30837344 |
| *Escherichia coli* | SPROECO17196 | U00096.3 | - | 14 | U00096.3:1961881-1961837 | 30904393 |
| *Escherichia coli* | SPROECO17198 | U00096.3 | - | 14 | U00096.3:225728-225772 | 30904393 |
| *Escherichia coli* | SPROECO17208 | U00096.3 | - | 14 | U00096.3:3796949-3796905 | 30904393 |
| *Escherichia coli* | SPROECO17217 | NC_000913.3 | VKRNDKILHYRGLN | 14 | NC_000913.3:85467-85511 | 29645342 |
| *Escherichia coli* | SPROECO16372 | NC_000913.3 | MERASKMPSSYLYDQ | 15 | NC_000913.3:175048-175095 | 30837344 |
| *Escherichia coli* | SPROECO16528 | MG1655 | MKHIPFFFAFFFTFP | 15 | MG1655:2737599-2737646 | 16397293 |
| *Escherichia coli* | SPROECO16704 | NC_000913.3 | MLRNRARRLDYKIMR | 15 | NC_000913.3:1407989-1408036 | 30837344 |
| *Escherichia coli* | SPROECO16793 | MG1655 | MNNSTKFCFSRFRTGN | 16 | MG1655:3948529-3948577 | 19734316 |
| *Escherichia coli* | SPROECO16794 | MG1655 | MNNSTKFCFSRFRTGN | 16 | MG1655:3948530-3948580 | 19121005 |
| *Escherichia coli* | SPROECO17002 | NC_000913.3 | MSIIILATISTTQENK | 16 | NC_000913.3:4486105-4486155 | 30837344 |
| *Escherichia coli* | SPROECO17138 | MG1655 | MTRVQFKHHHHHHHPD | 16 | MG1655:2089996-2090046 | 16397293 |
| *Escherichia coli* | SPROECO17194 | U00096.3 | - | 16 | U00096.3:1641589-1641539 | 30904393 |
| *Escherichia coli* | SPROECO17206 | U00096.3 | - | 16 | U00096.3:3111275-3111325 | 30904393 |
| *Escherichia coli* | SPROECO16707 | NC_000913.3 | MLSKGVLSARLFNLIYG | 17 | NC_000913.3:1298514-1298567 | 30837344 |
| *Escherichia coli* | SPROECO17164 | NC_000913.3 | MVLNVVLAGDFDCVRRP | 17 | NC_000913.3:3640643-3640696 | 30837344 |
| *Escherichia coli* | SPROECO17207 | U00096.3 | - | 17 | U00096.3:3640696-3640643 | 30904393 |
| *Escherichia coli* | SPROECO16334 | NC_000913.3 | MDTPSRYWLTILSSRINS | 18 | NC_000913.3:4001218-4001274 | 30837344 |
| *Escherichia coli* | SPROECO16469 | NC_000913.3 | MIGINVAIFTNVQRRTVS | 18 | NC_000913.3:391592-391648 | 30837344 |
| *Escherichia coli* | SPROECO17195 | U00096.3 | - | 18 | U00096.3:1642488-1642432 | 30904393 |
| *Escherichia coli* | SPROECO16467 | MG1655 | MIERELGNWKDFIEVMLRK | 19 | MG1655:2588831-2588888 | 19734316 |
| *Escherichia coli* | SPROECO16660 | NC_000913.3 | MKYLAFLIKGKTTHFDVGK | 19 | NC_000913.3:837503-837562 | 30837344 |
| *Escherichia coli* | SPROECO16923 | NC_000913.3 | MRLSANRQGNTCAGRSV- | 19 | NC_000913.3:837503-837562 | 30837344 |
| *Escherichia coli* | SPROECO17141 | NC_000913.3 | MTSDYRVSPIKKSRPAMSK | 19 | NC_000913.3:3838642-3838701 | 30837344 |
| *Escherichia coli* | SPROECO17205 | U00096.3 | - | 19 | U00096.3:3051023-3051082 | 30904393 |
| *Escherichia coli* | SPROECO16382 | NC_000913.3 | MEVGKLGKPYPLLNLAYVGV | 20 | NC_000913.3:2725925-2725987 | 30837344 |
| *Escherichia coli* | SPROECO16430 | MG1655 | MGQFFAYATVITVKENDHVA | 20 | MG1655:2085076-2085138 | 19121005 |
| *Escherichia coli* | SPROECO16431 | MG1655 | MGQFFAYATVITVKENDHVA | 20 | MG1655:2085078-2085138 | 19734316 |
| *Escherichia coli* | SPROECO16243 | NC_000913.3 | MAIIIGLEFAQLPMSFGAKYE | 21 | NC_000913.3:176552-176617 | 29645342 |
| *Escherichia coli* | SPROECO16378 | NC_000913.3 | METFCYMKWPVRHHKSRRVSH | 21 | NC_000913.3:850332-850397 | 29645342 |
| *Escherichia coli* | SPROECO16514 | NC_000913.3 | MKAPSGAFLLGVYSMDTHILR | 21 | NC_000913.3:1638188-1638253 | 29645342 |
| *Escherichia coli* | SPROECO16530 | MG1655 | MKHIQIRNSDMDWHIAANNLG | 21 | MG1655:1388892-1388957 | 19121005 |
| *Escherichia coli* | SPROECO16621 | MG1655 | MKRISTTITTTITITTGNGAG | 21 | MG1655:190-225 | 287010 |
| *Escherichia coli* | SPROECO16657 | MG1655 | MKYINCVYNINYKLKPHSHYK | 21 | MG1655:1463189-1463254 | 19121005 |
| *Escherichia coli* | SPROECO17187 | MG1655 | MYIFITHFFTEYVILKYLLPI | 21 | MG1655:1944139-1944204 | 19121005 |
| *Escherichia coli* | SPROECO17188 | MG1655 | MYIFITHFFTEYVILKYLLPI | 21 | MG1655:1944141-1944204 | 19734316 |
| *Escherichia coli* | SPROECO16438 | NC_000913.3 | MGWSLCFWHVSVRMTHDVVCYY | 22 | NC_000913.3:4518372-4518440 | 30837344 |
| *Escherichia coli* | SPROECO16783 | NC_000913.3 | MNLLVKCAGKIPALALTWTCRP | 22 | NC_000913.3:1491876-1491944 | 30837344 |
| *Escherichia coli* | SPROECO16488 | NC_000913.3 | MINQVSVYRQPPVLSGCRQVKTI | 23 | NC_000913.3:3032939-3033010 | 29645342 |
| *Escherichia coli* | SPROECO16656 | MG1655 | MKYFFMGISFMVIVWAGTFALMI | 23 | MG1655:2494829-2495011 | 19734316 |
| *Escherichia coli* | SPROECO16934 | NC_000913.3 | MR-VSKAGIISRRRLLLFQFAG | 23 | NC_000913.3:3720400-3720471 | 30837344 |
| *Escherichia coli* | SPROECO17201 | U00096.3 | - | 23 | U00096.3:2560142-2560071 | 30904393 |
| *Escherichia coli* | SPROECO17218 | MG1655 | VKYFFMGISFMVIVWAGTFALMI | 23 | MG1655:2494943-2495014 | 19121005 |
| *Escherichia coli* | SPROECO16581 | MG1655 | MKKTTIIMMGVAIIVVLGTELGWW | 24 | MG1655:1876898-1876972 | 19121005 |
| *Escherichia coli* | SPROECO16582 | MG1655 | MKKTTIIMMGVAIIVVLGTELGWW | 24 | MG1655:1878874-1878948 | 19734316 |
| *Escherichia coli* | SPROECO16719 | NC_000913.3 | MLYWLGILIAYADRRPDKVFTLIR | 24 | NC_000913.3:3640612-3640686 | 30837344 |
| *Escherichia coli* | SPROECO16763 | NC_000913.3 | MNIFKPISYIASLAPREVTLLALV | 24 | NC_000913.3:3797772-3797846 | 30837344 |
| *Escherichia coli* | SPROECO16765 | MG1655 | MNILHICVTSKWFNIDNKIVDHRP | 24 | MG1655:3888435-3888509 | 16397293 |
| *Escherichia coli* | SPROECO16799 | NC_000913.3 | MNQKFEAV-IDRNVTDVADANDR | 24 | NC_000913.3:922740-922814 | 30837344 |
| *Escherichia coli* | SPROECO16814 | NC_000913.3 | MNVSQIYARNGELFSGRICKQKRQ | 24 | NC_000913.3:3853766-3853840 | 30837344 |
| *Escherichia coli* | SPROECO17200 | U00096.3 | - | 24 | U00096.3:237325-237399 | 30904393 |
| *Escherichia coli* | SPROECO17209 | U00096.3 | - | 24 | U00096.3:3797846-3797772 | 30904393 |
| *Escherichia coli* | SPROECO17214 | NC_000913.3 | VCDILLNVLNIVFIGIAIILVIIC | 24 | NC_000913.3:331802-331876 | 29645342 |
| *Escherichia coli* | SPROECO16854 | MG1655 | MPRGGWPRKKPQKIVANDENYALAA | 25 | MG1655:2753658-2753733 | 2482406 |
| *Escherichia coli* | SPROECO16519 | MG1655 | MKDVDQIFDALDCHILREYLILLFYD | 26 | MG1655:2987841-2987921 | 19121005 |
| *Escherichia coli* | SPROECO16520 | MG1655 | MKDVDQIFDALDCHILREYLILLFYD | 26 | MG1655:2987843-2987921 | 19734316 |
| *Escherichia coli* | SPROECO16533 | MG1655 | MKIADQFHDELCRLAAINFEAHVLHG | 26 | MG1655:3305873-3305955 | 19121005 |
| *Escherichia coli* | SPROECO17093 | NC_000913.3 | MTIDKNWLNRSNKDPGRSLRFTHQPV | 26 | NC_000913.3:3884026-3884106 | 29645342 |
| *Escherichia coli* | SPROECO17197 | U00096.3 | - | 26 | U00096.3:2078419-2078499 | 30904393 |
| *Escherichia coli* | SPROECO16441 | NC_000913.3 | MHAISLHSLAYRRFARNVSLSMLLLMR | 27 | NC_000913.3:3109317-3109400 | 30837344 |
| *Escherichia coli* | SPROECO16535 | MG1655 | MKIILWAVLIIFLIGLLVVTGVFKMIF | 27 | MG1655:2227003-2227084 | 19734316 |
| *Escherichia coli* | SPROECO16536 | MG1655 | MKIILWAVLIIFLIGLLVVTGVFKMIF | 27 | MG1655:2227004-2227084 | 19121005 |
| *Escherichia coli* | SPROECO16694 | NC_000913.3 | MLMYQTRRTYQNSNNIAVVHLLKPAWR | 27 | NC_000913.3:3247668-3247751 | 30837344 |
| *Escherichia coli* | SPROECO17012 | NC_000913.3 | MSKNTKSKNNGIRKY-KTEVKLVYFK | 27 | NC_000913.3:3050958-3051041 | 29645342 |
| *Escherichia coli* | SPROECO17134 | NC_000913.3 | MTQRPWSKLQRKTHNIAALKIIARRSE | 27 | NC_000913.3:408947-409030 | 29645342 |
| *Escherichia coli* | SPROECO17159 | MG1655 | MVGRYRFEFILIILILCALITARFYLS | 27 | MG1655:1669801-1669884 | 19121005 |
| *Escherichia coli* | SPROECO16592 | MG1655 | MKLRKILKSMFNNYCKTFKDVPPGNMFR | 28 | MG1655:1985899-1985983 | 19734316 |
| *Escherichia coli* | SPROECO17090 | MG1655 | MTHIVRFIGLLLL-SSLRGRRVSGIQH | 28 | MG1655:83622-83708 | 16397293 |
| *Escherichia coli* | SPROECO17193 | U00096.3 | - | 28 | U00096.3:1295284-1295370 | 30904393 |
| *Escherichia coli* | SPROECO16235 | NC_000913.3 | MAEAFYILIGFLIMAAIIVMAVLYLENHS | 29 | NC_000913.3:643330-643419 | 29645342 |
| *Escherichia coli* | SPROECO16788 | NC_000913.3 | MNNPVCLDDWLIGFKSLCCTLAVIALLII | 29 | NC_000913.3:1642122-1642211 | 29645342 |
| *Escherichia coli* | SPROECO16963 | MG1655 | MSAGVITGVLLVFLLLGYLVYALI-EAF | 29 | MG1655:727957-728488 | ?9858692 |
| *Escherichia coli* | SPROECO16964 | MG1655 | MSAGVITGVLLVFLLLGYLVYALI-EAF | 29 | MG1655:728732-728821 | 16397293 |
| *Escherichia coli* | SPROECO17061 | MG1655 | MSTDLKFSLVTTIIVLGLIVAVGLTAALH | 29 | MG1655:1735480-1735569 | 19121005 |
| *Escherichia coli* | SPROECO17062 | MG1655 | MSTDLKFSLVTTIIVLGLIVAVGLTAALH | 29 | MG1655:1735482-1735569 | 19734316 |
| *Escherichia coli* | SPROECO17184 | MG1655 | MWYLLWFVGILLMCSLSTLVLVWLDPRLKS | 30 | MG1655:1039667-1039757 | 19734316 |
| *Escherichia coli* | SPROECO16673 | MG1655 | MLGNMNVFMAVLGIILFSGFLAAYFSHKWDD | 31 | MG1655:1620669-1620762 | 19734316 |
| *Escherichia coli* | SPROECO16796 | NC_000913.3 | MNNYTYKVNFNSISGVRHARIKCPIYTKNTF | 31 | NC_000913.3:290275-290370 | 30837344 |
| *Escherichia coli* | SPROECO16815 | MG1655 | MNVSSRTVVLINFFAAVGLFTLISMRFGWFI | 31 | MG1655:1515125-1515218 | 19734316 |
| *Escherichia coli* | SPROECO17203 | U00096.3 | - | 31 | U00096.3:290370-290275 | 30904393 |
| *Escherichia coli* | SPROECO16773 | NC_000913.3 | MNKLPAHLSRQNCKIASTNLSEIIPRRAAVLK | 32 | NC_000913.3:4536597-4536695 | 30837344 |
| *Escherichia coli* | SPROECO16909 | MG1655 | MRIGIIFPVVIFITAVVFLAWFFIGGYAAPGA | 32 | MG1655:1876796-1876892 | 19734316 |
| *Escherichia coli* | SPROECO16910 | MG1655 | MRIGIIFPVVIFITAVVFLAWFFIGGYAAPGA | 32 | MG1655:1876797-1876895d | 19121005 |
| *Escherichia coli* | SPROECO17072 | MG1655 | MTALLRVISLVVISVVVIIIPPCGAALGRGKA | 32 | MG1655:3950322-3950420 | 16397293 |
| *Escherichia coli* | SPROECO17145 | MG1655 | MTTSML-KLLPTAPSAAVVVVRVVVVVG-P | 32 | MG1655:3852890-3852988 | 16397293 |
| *Escherichia coli* | SPROECO17147 | NC_000913.3 | MTTSML-KLLPTAPSAAVVVVRVVVVVG-P | 32 | NC_000913.3:3852890-3852988 | 6292893 |
| *Escherichia coli* | SPROECO16327 | NC_000913.3 | MDNLFRTLFSTFTHLRTSSTILLVGEQHWR-L | 33 | NC_000913.3:1673608-1673709 | ?30837344 |
| *Escherichia coli* | SPROECO16523 | MG1655 | MKENKVQQISHKLINIVVFVAIVEYAYLFLHFY | 33 | MG1655:312365-312466 | 19121005 |
| *Escherichia coli* | SPROECO16524 | MG1655 | MKENKVQQISHKLINIVVFVAIVEYAYLFLHFY | 33 | MG1655:312367-312466 | 19734316 |
| *Escherichia coli* | SPROECO16871 | NC_000913.3 | MPVNGIFDVFDMLSIYIIYKLIVSNNTWLIMRK | 33 | NC_000913.3:4556574-4556675 | 29645342 |
| *Escherichia coli* | SPROECO16947 | MG1655 | MRSEQISGSSLNPSCRFSSAYSPVTRQRKDMSR | 33 | MG1655:4452223-4452324 | 16397293 |
| *Escherichia coli* | SPROECO16759 | NC_000913.3 | MNGDNPSPNRPLVTVVYKGPDFYDGEKKPPVNRR | 34 | NC_000913.3:1622741-1622845 | 30837344 |
| *Escherichia coli* | SPROECO16862 | MG1655 | MPTKRFDKKHWKMVVVLLAICGAMLLLRWAAMIWG | 35 | MG1655:1803188-1803293 | 19734316 |
| *Escherichia coli* | SPROECO16904 | MG1655 | MRIAKIGVIALFLFMALGGIGGVMLAGYTFILRAG | 35 | MG1655:2213678-2213783 | 19734316 |
| *Escherichia coli* | SPROECO16921 | NC_000913.3 | MRKSYEVGISPKINLCNSVEVLTNSFGTVISGRQV | 35 | NC_000913.3:754674-754781 | 29645342 |
| *Escherichia coli* | SPROECO17088 | MG1655 | MTFAELGMAFWHDLAAPVIAGILASMIVNWLNKRK | 35 | MG1655:3699980-3700087 | 16397293 |
| *Escherichia coli* | SPROECO17107 | MG1655 | MTLAQFAMIFWHDLAAPILAGIITAAIVSWWRNRK | 35 | MG1655:1269168-1269275 | 16397293 |
| *Escherichia coli* | SPROECO17108 | MG1655 | MTLAQFAMIFWHDLAAPILAGIITAAIVSWWRNRK | 35 | MG1655:1270238-1270345 | 16397293 |
| *Escherichia coli* | SPROECO17110 | MG1655 | MTLAQFAMTFWHDLAAPILAGIITAAIVGWWRNRK | 35 | MG1655:1269703-1269810 | 16397293 |
| *Escherichia coli* | SPROECO16343 | NC_000913.3 | MEIKVQRLSLWMINTVFLLSPINNHQTNTINLIFEM | 36 | NC_000913.3:1212540-1212650 | 29645342 |
| *Escherichia coli* | SPROECO16473 | NC_000913.3 | MIIGMLRAHMITSLSPIPTMPSVNINKPKARFLYAI | 36 | NC_000913.3:3798039-3798149 | 30837344 |
| *Escherichia coli* | SPROECO16671 | MG1655 | MLESIINLVSSGAVDSHTPQTAVAAVLCAAMIGLFS | 36 | MG1655:4051670-4051780 | 19121005 |
| *Escherichia coli* | SPROECO17096 | NC_000913.3 | MTIEKHERSTKDLVKAAVSGWLGTALEFMDFKSHAC | 36 | NC_000913.3:1652728-1652838 | 29645342 |
| *Escherichia coli* | SPROECO16740 | NC_000913.3 | MMTTLLPVFTKPSPLAL-LRAGRICRFLLIPDGRIR | 37 | NC_000913.3:3630665-3630778 | 30837344 |
| *Escherichia coli* | SPROECO16808 | NC_000913.3 | MNSILIITSLLIIFSIFSHALIKLGIGISNNPDKTDV | 37 | NC_000913.3:1434406-1434293 | 29645342 |
| *Escherichia coli* | SPROECO16809 | NC_000913.3 | MNSILIITSLLIIFSIFSHALIKLGIGISNNPDKTDV | 37 | NC_000913.3:1632890-1633003 | 29645342 |
| *Escherichia coli* | SPROECO17183 | MG1655 | MWYFAWILGTLLACSFGVITALALEHVESGKAGQEDI | 37 | MG1655:773418-773529 | 25475368 |
| *Escherichia coli* | SPROECO16655 | MG1655 | MKVRASVKKLCRNCKIVKRDGVIRVICSAEPKHKQRQG | 38 | MG1655:3442618-3442734 | 16397293 |
| *Escherichia coli* | SPROECO16860 | MG1655 | MPSTQYIRRPASSYASCIWCTTACASCHGRTTKPSLAT | 38 | MG1655:1806659-1806755 | 12003040 |
| *Escherichia coli* | SPROECO16820 | NC_000913.3 | MNYKAFTQIAIDLLSAKLCNCTQAIMTHIIASFLAFMFF | 39 | NC_000913.3:383960-384079 | 29645342 |
| *Escherichia coli* | SPROECO17089 | NC_000913.3 | MTGIKKITQTFSLRQLTFLKGATAKNVRECNLMKNSVAEH | 40 | NC_000913.3:1079024-1079146 | 29645342 |
| *Escherichia coli* | SPROECO17098 | NC_000913.3 | MTKHPTGIYVGCLVKVIRRRLRMELKESVINYSPFVLQHP | 40 | NC_000913.3:1579545-1579667 | 29645342 |
| *Escherichia coli* | SPROECO16479 | MG1655 | MIKNFIFDNLIILAVPFMIKTSLKTNLIFFFLCVFVPHMAS | 41 | MG1655:4504470-4504593 | 19734316 |
| *Escherichia coli* | SPROECO16480 | MG1655 | MIKNFIFDNLIILAVPFMIKTSLKTNLIFFFLCVFVPHMAS | 41 | MG1655:4504471-4504596 | 19121005 |
| *Escherichia coli* | SPROECO16725 | MG1655 | MMKRLIVLVLLASTLLTGCNTARGFGEDIKHLGNSISRAAS | 41 | MG1655:4376317-4376442 | 16397293 |
| *Escherichia coli* | SPROECO16756 | MG1655 | MNFLMRAIFSLLLLFTLSIPVISDCVAMAIESRFKYMMLLF | 41 | MG1655:3030839-3030964 | 19121005 |
| *Escherichia coli* | SPROECO16805 | MG1655 | MNRLIELTGWIVLVVSVILLGVASHIDNYQPPEQSASVQHK | 41 | MG1655:1704551-1704676 | 16397293 |
| *Escherichia coli* | SPROECO17204 | U00096.3 | - | 41 | U00096.3:296087-296212 | 30904393 |
| *Escherichia coli* | SPROECO16752 | MG1655 | MNEFKRCMRVFSHSPFKVRLMLLSMLCDMVNNKPQQDKPSDK | 42 | MG1655:852094-852220 | 21908668 |
| *Escherichia coli* | SPROECO16883 | NC_000913.3 | MQKLPLKEKCLTATANYHPGIRYIMTGYSAKYIYSSTYARFR | 42 | NC_000913.3:1344647-1344775 | 30837344 |
| *Escherichia coli* | SPROECO16927 | NC_000913.3 | MRMIGLLYDFKDYASKMAENMARLAALLHYFSGDGGDISVTG | 42 | NC_000913.3:290510-290638 | 29645342 |
| *Escherichia coli* | SPROECO16565 | MG1655 | MKKKPVAQLERQHSLLENPCAYGLLSQFQAAIVVNCFTLNKII | 43 | MG1655:3083944-3084073 | 19734316 |
| *Escherichia coli* | SPROECO17165 | MG1655 | MVQCVRHFVLPRLKKDAGLPFFFPLITHSQPLNRGAFFCPGVRR | 44 | MG1655:4472399-4472533 | 16397293 |
| *Escherichia coli* | SPROECO16644 | MG1655 | MKSNRQARHILGLDHKISNQRKIVTEGDKSSVVNNPTGRKRPAEK | 45 | MG1655:1555826-1555963 | 16397293 |
| *Escherichia coli* | SPROECO16637 | MG1655 | MKRTFQPSVLKRNRSHGFRARMATKNGRQVLARRRAKGRARLTVSK | 46 | MG1655:3884336-3884476 | 16397293 |
| *Escherichia coli* | SPROECO16651 | MG1655 | MKVLNSLRTAKERHPDCQIVKRKGRLYVICKSNPRFKAVQGRKKKR | 46 | MG1655:311600-311738 | 19734316 |
| *Escherichia coli* | SPROECO16977 | MG1655 | MSEENKENGFNHVKTFTKIIFIFSVLVFNDNEYKITDAAVNLFIQI | 46 | MG1655:2903579-2903719 | 19121005 |
| *Escherichia coli* | SPROECO16306 | MG1655 | MCGIFSKEVLSKHVDVEYRFSAEPYIGASCSNVSVLSMLCLRAKKTI | 47 | MG1655:1905474-1905615 | 19734316 |
| *Escherichia coli* | SPROECO16554 | MG1655 | MKKFRWVVLVVVVLACLLLWAQVFNMMCDQDVQFFSGICAINQFIPW | 47 | MG1655:1906649-1908135 | 19734316 |
| *Escherichia coli* | SPROECO16801 | NC_000913.3 | MNRCLLLNLSHRSGEDSFPALCISALHTCRCYTHLGASQDSRAGYSY | 47 | NC_000913.3:3086145-3086288 | 30837344 |
| *Escherichia coli* | SPROECO16855 | NC_000913.3 | MPRLTAKDFPQELLDYYDYYAHGKISKREFLNLAAKCGRRDDGISVV | 47 | NC_000913.3:3147597-3147740 | 29645342 |
| *Escherichia coli* | SPROECO16428 | NC_000913.3 | MGNDAFKLSSADRGDITINNESGHLIVNTAILSGDIVTLRGGEIRLVL | 48 | NC_000913.3:2470497-2470643 | 30837344 |
| *Escherichia coli* | SPROECO17163 | MG1655 | MVKKTIAAIFSVLVLSTVLTACNTTRGVGEDISDGG-ISGAATKAQQ | 48 | MG1655:4376553-4376699 | 16397293 |
| *Escherichia coli* | SPROECO16532 | MG1655 | MKHNPLVVCLLIICITILTFTLLTRQTLYELRFRDGDKEVAALMACTSR | 49 | MG1655:1491922-1492071 | 16397293 |
| *Escherichia coli* | SPROECO16669 | MG1655 | MLELLKSLVFAVIMVPVVMAIILGLIYGLGEVFNIFSGVGKKDQPGQNH | 49 | MG1655:793995-794142 | 23010927 |
| *Escherichia coli* | SPROECO17025 | NC_000913.3 | MSLVLCFLLMSLFFMYSFVLSRLWRKKIAIRLLLYIQDNVTLIVFLNKK | 49 | NC_000913.3:568695-568844 | 29645342 |
| *Escherichia coli* | SPROECO16612 | MG1655 | MKQHKAMIVALIVICITAVVAALVTRKDLCEVHIRTGQTEVAVFTAYESE | 50 | MG1655:16751-16903 | 16397293 |
| *Escherichia coli* | SPROECO16709 | MG1655 | MLTKYALAAVIVLCLTVLGFTLLVGDSLCEFTVKERNIEFKAVLAYEPKK | 50 | MG1655:607836-607988 | 16397293 |
| *Escherichia coli* | SPROECO16851 | MG1655 | MPQKYRLLSLIVICFTLLFFTWMIRDSLCELHIKQESYELAAFLACKLKE | 50 | MG1655:3720448-3720600 | 16397293 |
| *Escherichia coli* | SPROECO17199 | U00096.3 | - | 50 | U00096.3:2266109-2265957 | 30904393 |
| *Escherichia coli* | SPROECO16501 | NC_000913.3 | MIRLQHDKQKQMRYGTLQKRDTLTLCLLKLQLMEWRFDSAWKFGLGRLYLG | 51 | NC_000913.3:---:- | 28924029 |
| *Escherichia coli* | SPROECO16614 | MG1655 | MKQQKAMLIALIVICLTVIVTALVTRKDLCEVRIRTGQTEVAVFTAYEPEE | 51 | MG1655:1645119-1645274 | 16397293 |
| *Escherichia coli* | SPROECO16845 | NC_000913.3 | MPMIKSPHGEGGCVCAPPATDWTPPPLLPLLNRFDFRSTRPQTLLRRGGSNYGY | 54 | NC_000913.3:1359177-1359341 | 29645342 |
| *Escherichia coli* | SPROECO16250 | MG1655 | MAKGIREKIKLVSSAGTGHFYTTTKNKRTKPEKLELKKFDPVVRQHVIYKEAKIK | 55 | MG1655:3811250-3811417 | 16397293 |
| *Escherichia coli* | SPROECO16634 | MG1655 | MKRQKRDRLERAHQRGYQAGIAGRSKEMCPYQTLNQRSQWLGGWREAMADRVVMA | 55 | MG1655:1015715-1015882 | 16397293 |
| *Escherichia coli* | SPROECO16781 | MG1655 | MNLAIQILASYPPSGKEKGYEAQPSGGVSAHYLHYDSDIHTPDPT-LRTAVPGR | 55 | MG1655:1491962-1492129 | 16397293 |
| *Escherichia coli* | SPROECO16394 | NC_000913.3 | MFIAWYWIVLIALVVVGYFLHLKRYCRAFRQDRDALLEARNKYLNSTREETAEKVE | 56 | NC_000913.3:3183381-3183551 | 29645342 |
| *Escherichia coli* | SPROECO16983 | NC_000913.3 | MSERKNSKSRRNYLVKCSCPNCTQESEHSFSRVQKGALLICPHCNKVFQTNLKAVA | 56 | NC_000913.3:1640805-1640975 | 30837344 |
| *Escherichia coli* | SPROECO16301 | MG1655 | MAVQQNKPTRSKRGMRRSHDALTAVTSLSVDKTSGEKHLRHHITADGYYRGRKVIAK | 57 | MG1655:1147367-1147540 | 16397293 |
| *Escherichia coli* | SPROECO16429 | MG1655 | MGNLPVGSRGLYKGKKRSAGMNELRHPGSTQMTRTGQAHRGGEHRLNTGPPDWQTVE | 57 | MG1655:36336-36506 | 11169109 |
| *Escherichia coli* | SPROECO16979 | NC_000913.3 | MSEFDAQRVAERIDIVLDILVAGDYHSAIHNLEILKAELLRQVAESTPDIPKAPWEI | 57 | NC_000913.3:1344436-1344609 | 29645342 |
| *Escherichia coli* | SPROECO17144 | NC_000913.3 | MTTLIYLQIPVPEPIPGDPVPVPDPIPRPQPMPDPPPDEEPIKLSHRERRSARIRAC | 57 | NC_000913.3:1409308-1409481 | 29645342 |
| *Escherichia coli* | SPROECO16478 | NC_000913.3 | MIKIFIGHYINVFYSTADITLKKQPLLFLAKLMVYSAALTFFTANFHCNMTRKINEYA | 58 | NC_000913.3:1995655-1995831 | 29645342 |
| *Escherichia coli* | SPROECO16256 | MG1655 | MAKTIKITQTRSAIGRLPKHKATLLGLGLRRIGHTVEREDTPAIRGMI-VSFMVKVEE | 59 | MG1655:3444543-3444722 | 16397293 |
| *Escherichia coli* | b4513 | NC_000913.3 | MSAGVITGVLLVFLLLGYLVYALI-EAF | 29 | NC_000913.3:728732-728821 | 16738553 |
| *Escherichia coli* | b4409 | U00096.3 | MNRLIELTGWIVLVVSVILLGVASHIDNYQPPEQSASVQHK | 41 | U00096.3:1704551-1704676 | 16738553 |
| *Escherichia coli* | b4410 | U00096.3 | MMKRLIVLVLLASTLLTGCNTARGFGEDIKHLGNSISRAAS | 41 | U00096.3:4376317-4376442 | 9278503 |
| *Escherichia coli* | b4606 | U00096.3 | MIERELGNWKDFIEVMLRK | 19 | U00096.3:2590807-2590866 | 9278503 |
| *Escherichia coli* | b4599 | U00096.3 | MLGNMNVFMAVLGIILFSGFLAAYFSHKWDD | 31 | U00096.3:1622646-1622741 | 16397293 |
| *Escherichia coli* | b4522 | NC_000913.3 | MRLAMPSGNQEPRRDPELKRKAWLAVFLGSALFWVVVALLIWKVWG | 46 | NC_000913.3:1335148-1335288 | 16397293 |
| *Escherichia coli* | b4662 | U00096.3 | MRQFYQHYFTATAKLCWLRWLSVPQRLTMLEGLMQWDDRNSES | 43 | U00096.3:77388-77519 | 9278503 |
| *Escherichia coli* | b0762 | U00096.3 | MLELLKSLVFAVIMVPVVMAIILGLIYGLGEVFNIFSGVGKKDQPGQNH | 49 | U00096.3:794773-794922 | 9278503 |
| *Escherichia coli* | b4669 | U00096.3 | MNNSTKFCFSRFRTGN | 16 | U00096.3:3950507-3950557 | 16738553 |
| *Escherichia coli* | b4678 | U00096.3 | MGQFFAYATVITVKENDHVA | 20 | U00096.3:2087052-2087114 | 16397293 |
| *Escherichia coli* | b4677 | U00096.3 | MYIFITHFFTEYVILKYLLPI | 21 | U00096.3:1946115-1946180 | 16397293 |
| *Escherichia coli* | b4674 | U00096.3 | MKYINCVYNINYKLKPHSHYK | 21 | U00096.3:1465165-1465230 | 9278503 |
| *Escherichia coli* | b4680 | U00096.3 | MKYFFMGISFMVIVWAGTFALMI | 23 | U00096.3:2496921-2496992 | 16738553 |
| *Escherichia coli* | b4675 | U00096.3 | MKKTTIIMMGVAIIVVLGTELGWW | 24 | U00096.3:1878874-1878948 | 16738553 |
| *Escherichia coli* | b4685 | U00096.3 | MKIADQFHDELCRLAAINFEAHVLHG | 26 | U00096.3:3307853-3307933 | 9278503 |
| *Escherichia coli* | b4679 | U00096.3 | MKIILWAVLIIFLIGLLVVTGVFKMIF | 27 | U00096.3:2228982-2229065 | 9278503 |
| *Escherichia coli* | b4601 | U00096.3 | MVGRYRFEFILIILILCALITARFYLS | 27 | U00096.3:1671777-1671860 | 16397293 |
| *Escherichia coli* | b4602 | CP045977.1 | MSTDLKFSLVTTIIVLGLIVAVGLTAALH | 29 | CP045977.1:1651891-1651980 | 16397293 |
| *Escherichia coli* | b4686 | U00096.3 | MLESIINLVSSGAVDSHTPQTAVAAVLCAAMIGLFS | 36 | U00096.3:4053647-4053757 | 16738553 |
| *Escherichia coli* | BSNT_09633 | - | MSAGVITGVLLVFLLLGYLVYALI-EAF | 29 | - | 25329997 |
| *Escherichia coli* | WP_352432960 | - | MIYAIAGGARMGAFQLNESLLERITRKLRDGWKRVEV | 37 | - | 17890311 |
| *Eubacterium limosum* | ACH52_2426 | CP011914.1 | METITIATGILGLGLMGYLFYVLFKGENL | 29 | CP011914.1:2574766-2574855 | 26981167 |
| *Eubacterium limosum* | ACH52_2189 | CP011914.1 | MINEEFVYCNVDFSTSEEMLTTVSKDLFIAGFVTDMYGKK | 40 | CP011914.1:2321864-2321986 | 26981167 |
| *Klebsiella michiganensis* | KOX_14695 | CP003218.1 | MWYFAWILGTLLACAFGVITALALEHVEATKAGKEEH | 37 | CP003218.1:3189603-3189716 | [22493189](https://www.ncbi.nlm.nih.gov/pubmed/22493189) |
| *Klebsiella michiganensis* | KOX_08745 | CP003218.1 | MVKKAIAAFFTVLALSSVLTACNTTRGVGQDISEGGSAISGAATKAQQ | 48 | CP003218.1:1859916-1860062 | [22493189](https://www.ncbi.nlm.nih.gov/pubmed/22493189) |
| *Klebsiella michiganensis* | KOX_13830 | CP003218.1 | MLTKYALVAIIVLCLTALGLTLMVRDSLCELSIKERSMEFKAVLAYETKK | 50 | CP003218.1:2994604-2994756 | [22493189](https://www.ncbi.nlm.nih.gov/pubmed/22493189) |
| *Klebsiella michiganensis* | KOX_17645 | CP003218.1 | MLAVQLLQEMISEEPGARERAIEFLRLFGDQP | 32 | CP003218.1:3821254-3821352 | [22493189](https://www.ncbi.nlm.nih.gov/pubmed/22493189) |
| *Klebsiella michiganensis* | KOX_17815 | CP003218.1 | MTYDFERLMEGAKLLKCSEFGDAIIANM | 28 | CP003218.1:3860807-3860893 | [22493189](https://www.ncbi.nlm.nih.gov/pubmed/22493189) |
| *Klebsiella michiganensis* | KOX_20600 | CP003218.1 | MVKMFFNVLATFVEFEGDLIRLRTREDMAIPRGKENCGGSSRRC | 44 | CP003218.1:4450341-4450475 | [22493189](https://www.ncbi.nlm.nih.gov/pubmed/22493189) |
| *Klebsiella michiganensis* | KOX_21955 | CP003218.1 | MENVFNRIVELIGWIVLGVSALLLVIAHHIDNYQSPPAVDAVQTKPLSK | 49 | CP003218.1:4734316-4734465 | [22493189](https://www.ncbi.nlm.nih.gov/pubmed/22493189) |
| *Klebsiella michiganensis* | KOX_23700 | CP003218.1 | MKKLRWVLLIVIIAGCLLLWTQMLNVMCDQDVQFFSGICTINKFIPW | 47 | CP003218.1:5114298-5114441 | [22493189](https://www.ncbi.nlm.nih.gov/pubmed/22493189) |
| *Klebsiella michiganensis* | A225_1789 | CP003683.1 | MLELLKSLVFAVVMVPVVMAVILGLIYGLGEVFNLFSGAGHKDSRPQNH | 49 | CP003683.1:1883137-1883286 | 22965083 |
| *Klebsiella michiganensis* | A225_1941 | CP003683.1 | MSTVHVEVEGDIKFPIMPENFNLVFEQFFMSNINYTYQIWKKG | 43 | CP003683.1:2047891-2048022 | 22965083 |
| *Klebsiella michiganensis* | A225_2656 | CP003683.1 | MQTVTKAFGPSHKAFLVVPMVGAFIVDISNSILIKIFIEIGTYFT | 45 | CP003683.1:2807651-2807788 | 22965083 |
| *Klebsiella michiganensis* | A225_2657 | CP003683.1 | MKLRHLDIFYAVMTCGSLTRAAEVLHISQPAASKALKHAEH | 41 | CP003683.1:2808019-2808144 | 22965083 |
| *Klebsiella michiganensis* | A225_3266 | CP003683.1 | MENVFNRIVELIGWIVLGVSALLLVIAHHIDNYQSPPAVDAVQTKPLSK | 49 | CP003683.1:3444585-3444734 | 22965083 |
| *Klebsiella michiganensis* | A225_3631 | CP003683.1 | MKKLRWVLLIVIIAGCLLLWTQMLNVMCDQDVQFFSGICTINKFIPW | 47 | CP003683.1:3832138-3832281 | 22965083 |
| *Klebsiella michiganensis* | A225_4423 | CP003683.1 | MQKEKLSALMDGETLDNELLNELERSSEMQKNLGELPSHPRYAARRYE | 48 | CP003683.1:4663105-4663251 | 22965083 |
| *Klebsiella michiganensis* | A225_4648 | CP003683.1 | MLTKYALVAIVVLCITVLGFTLLVRSSLCELSIKERSMEFKAVLAYESKK | 50 | CP003683.1:4884946-4885098 | 22965083 |
| *Klebsiella michiganensis* | A225_5546 | CP003683.1 | MPLDVRKWSCPECGADHDRDINAARNIKAAGLAVLACGVAVNP | 43 | CP003683.1:5779281-5779412 | 22965083 |
| *Klebsiella michiganensis* | A225_5586 | CP003683.1 | MFVHFSAIQNEGYKSLDEGQKVSFTIESGAKGPAAGNVTSL | 41 | CP003683.1:5824341-5824466 | 22965083 |
| *Klebsiella michiganensis* | A225_5588 | CP003683.1 | MPQKYLLFSLIVICFTILLFTWMVRDSLCELQLRQGNIELVAFLACGIKT | 50 | CP003683.1:5826488-5826640 | 22965083 |
| *Klebsiella michiganensis* | A225_0417 | CP003683.1 | MVKKAIAAFFTVLALSSVLTACNTTRGVGQDISEGGSAISGAATKAQQ | 48 | CP003683.1:437561-437707 | 22965083 |
| *Klebsiella michiganensis* | A225_1763 | CP003683.1 | MWYFAWILGTLLACAFGVITALALEHVEATKAGKEEH | 37 | CP003683.1:1862479-186259 | 22965083 |
| *Listeria innocua* | lin_2569 | AL596172.1 | MTIDDLEMMTLGSCLDYFAEYEEAQNPNKKKKARRASQADFDAF | 44 | AL596172.1:236756-236890 | [11679669](https://www.ncbi.nlm.nih.gov/pubmed/11679669) |
| *Listeria innocua* | lin_2406 | AL596172.1 | MMTVAELIEKLKELPANADILLTIGWNHSEIEEVGCIENERNVYISGW | 48 | AL596172.1:70836-70982 | [11679669](https://www.ncbi.nlm.nih.gov/pubmed/11679669) |
| *Listeria innocua* | K1T44_0085 | CP084086.1 | MAKLASFYPIVATPKRDGYKEYLPSVQMIEISREII | 36 | CP084086.1:72075-72185 | [35007911](https://www.ncbi.nlm.nih.gov/pubmed/35007911) |
| *Listeria innocua* | K1T44_0692 | CP084086.1 | MYEYNYNSKRNTPYHINLLVFKDGSSQEDDQVKHPPLQEQKD | 48 | CP084086.1:703985-704113 | 35007911 |
| *Listeria innocua* | K1T44_1190 | CP084086.1 | MNKVKLFLHHPATIFTMKTIFYLTVLLGLLWFYGFKNPEGAKFIYNEF | 48 | CP084086.1:1195679-1195825 | 35007911 |
| *Listeria innocua* | K1T44_2496 | CP084086.1 | MINWYEKVKDYFLGGYYTEADVNKFVTLKKITRSQADVIIAMKEAKAE | 48 | CP084086.1:2496807-2496953 | 35007911 |
| *Listeria innocua* | K1T44_2760 | CP084086.1 | MEVFVVMIFVSFMSVIAGYWLRGSDKKHG | 29 | CP084086.1:2704039-2704128 | 35007911 |
| *Lacticaseibacillus rhamnosus* | LOCK908_2451 | CP005485.1 | MKKFKDEEMPMMTDLELSSFVGGMLRDEGFTFNKFKNWLTSIFG | 44 | CP005485.1:2452465-2452599 | 24558250 |
| *Lacticaseibacillus rhamnosus* | LOCK908_2476 | CP005485.1 | MPITHSPAQKSACKDLKRNGQVRAITLKATYVLAYAHNALPGAGICV | 47 | CP005485.1:2469355-2469498 | 24558250 |
| *Lacticaseibacillus rhamnosus* | LOCK908_2626 | CP005485.1 | MAKARPSRLRPLIWFTPITRSPAQKPAHKDLGRNGQSPASHHV | 43 | CP005485.1:2610572-2610703 | 24558250 |
| *Lacticaseibacillus rhamnosus* | LOCK908_2670 | CP005485.1 | MLTITRSPAQKSECKDLKRNGQERAITLKAAYTPVSNRAGSRSLS | 45 | CP005485.1:2647905-2648042 | 24558250 |
| *Lacticaseibacillus rhamnosus* | LOCK908_0106 | CP005485.1 | MRSLAQKSACKDLGRNGQNPTITAKAAYTPTSNRAGSRSL | 40 | CP005485.1:116890-117012 | 24558250 |
| *Lacticaseibacillus rhamnosus* | LOCK908_0239 | CP005485.1 | MAFTPITRSPAQKPAHKDLERNGQSPAITPKATYAPASNRAGSRF | 45 | CP005485.1:241656-241793 | 24558250 |
| *Lacticaseibacillus rhamnosus* | LOCK908_0276 | CP005485.1 | MRDFGWLALILTRSLAQGSACKNLGRNGQAQAITPKATYTPIPNRTGSRS | 50 | CP005485.1:277152-277304 | 24558250 |
| *Lacticaseibacillus rhamnosus* | LOCK908_0578 | CP005485.1 | MRLPVQKSACKNLGRNGQSATITPKATYTPASNRAGSRSS | 40 | CP005485.1:580413-580535 | 24558250 |
| *Lacticaseibacillus rhamnosus* | LOCK908_0770 | CP005485.1 | MFAKLKAVWENPTFRFIFWTVTYFVILLVLVYLYGYSGINNSKFIYNEF | 49 | CP005485.1:777192-777341 | 24558250 |
| *Lacticaseibacillus rhamnosus* | LOCK908_1530 | CP005485.1 | MLQKPFIITRSPAQKSACKDPLSAMAKTRPSRLRPLTLRLLNAPVHAHFL | 50 | CP005485.1:1488457-1488609 | 24558250 |
| *Lacticaseibacillus rhamnosus* | LOCK908_1570 | CP005485.1 | MILFSLAFTPITRSPAQKSACKDLRHNGQNRAITSEATISPLRP | 44 | CP005485.1:1530117-1530251 | 24558250 |
| *Lacticaseibacillus rhamnosus* | LOCK900_2275 | CP005485.1 | MRYVDLSMPIQTAAAFDYAKADERLVLTLGSVGIKSGALSISYWK | 45 | CP005484.1:2271130-2271267 | 24558250 |
| *Lacticaseibacillus rhamnosus* | LOCK900_0095 | CP005485.1 | MRSLAQKSACKDLGRNGQNPTITTQAAYTPTSNRAGSRSGGF | 42 | CP005484.1:98329-98457 | 24558250 |
| *Lacticaseibacillus rhamnosus* | LRHK_2890 | CP003094.1 | MQMRVIKGCKMEGKEELITTIQDLMDKYKTGIDYLKDK | 38 | CP003094.1:2807661-2807777 | 22247527 |
| *Lacticaseibacillus rhamnosus* | LRHK_2763 | CP003094.1 | MSVADALMLMLVFGDFVLSLIALIITIMLIIQDNQKDRR | 39 | CP003094.1:2681792-2681911 | 22247527 |
| *Lacticaseibacillus rhamnosus* | LRHK_2640 | CP003094.1 | MAYIQIMEMVYNGEISKEDLYSAEGKKTMKSLFKNQLKNSSNVEVNG | 47 | CP003094.1:2575242-2575385 | 22247527 |
| *Lacticaseibacillus rhamnosus* | LRHK_2441 | CP003094.1 | MVQKNGKHAYPATGEGQTGLLLAEAGAAIIAALGFAGVRKARHAK | 45 | CP003094.1:2390003-2390140 | 22247527 |
| *Lacticaseibacillus rhamnosus* | LRHK_2394 | CP003094.1 | MKKFKDEEMPMMTDLELSSFVGGMLRDEGFTFNKFKNWLTSIFG | 44 | CP003094.1:2354073-2354207 | 22247527 |
| *Lacticaseibacillus rhamnosus* | LRHK_1816 | CP003094.1 | MTGRLALTITRSPAQKPAHKDLGRNDQSPVITPKATYAPVSNRAGSRSP | 49 | CP003094.1:1764795-1764944 | 22247527 |
| *Lacticaseibacillus rhamnosus* | LRHK_1041 | CP003094.1 | MSNLKKANHLPLSIITIVAGSLSIIAGAMNLASGILLLVKSRHSPS | 46 | CP003094.1:992537-992677 | 22247527 |
| *Lacticaseibacillus rhamnosus* | LRHK_775 | CP003094.1 | MFAKLKAVWENPTFRFIFWTVTYFVILLVLVYLYGYSGINNSKFIYNEF | 49 | CP003094.1:766196-766345 | 22247527 |
| *Lacticaseibacillus rhamnosus* | LRHK_489 | CP003094.1 | MDKELLTYVLDQAVGVVIAVILLTRIERRLDTLITHIETFMAKLDGKQLR | 50 | CP003094.1:480527-480679 | 22247527 |
| *Lacticaseibacillus rhamnosus* | LRHK_96 | CP003094.1 | MKQTKEIKRWPFSLSLFSQQPSRYNNQIWRDRHDQTSY | 38 | CP003094.1:99882-99998 | 22247527 |
| *Listeria_monocytogenes* | LMON_1217 | HG421741.1 | MKRSALWKDIYREIWRSKSRFISIFMLIMLGVAFSPD | 37 | HG421741.1:1204346-1204459 | 24667708 |
| *Listeria_monocytogenes* | LMON_1243 | HG421741.1 | MKLLRFFGLISIDENGNEYIEKSDRYTLVCLALTVLIALVVGIGGLILNG | 50 | HG421741.1:1229781-1229933 | 24667708 |
| *Listeria_monocytogenes* | LMON_1247 | HG421741.1 | MTVAELIEKLKELPANADILLTIGWNHSEIEEVGCIENERNVYISGW | 47 | HG421741.1:1231793-1231936 | 24667708 |
| *Listeria_monocytogenes* | LMON_1398 | HG421741.1 | MRVNITLECTECGDRNYITTKNKRENPERIELKKYCPRLRRVTLHRETK | 49 | HG421741.1:1366332-1366481 | 24667708 |
| *Listeria_monocytogenes* | LMON_1581 | HG421741.1 | MEDEEAAQRELWTRMRNAVRDVLDQTTLADLLKHSTDSELTDGYMFYI | 48 | HG421741.1:1550809-1550955 | 24667708 |
| *Listeria_monocytogenes* | LMON_2012 | HG421741.1 | MNLEKELKEYLGFDEFRPGQKEVIETALAKQIVLLCYLLEQEKPFVIN | 48 | HG421741.1:2017275-2017421 | 24667708 |
| *Listeria_monocytogenes* | LMON_2630 | HG421741.1 | MKVRPSVKPMCEKCKVIRRKGKVMVICENPKHKQKQG | 37 | HG421741.1:2660987-2661100 | 24667708 |
| *Mycobacteroides abscessus* | P3M62_10545 | CP119724.1 | MGDEGVKQFNVYLPLSLIRQVKHRAIETDMSLSALVAEALRDYLDSDER | 49 | CP119724.1:2158730-2158879 | 37364635 |
| *Mycobacteroides abscessus* | P3M62_18940 | CP119724.1 | MAEPTTVTGQHNDTELVQESLVEEVSIDGMCGVY | 34 | CP119724.1:3826976-3827080 | 37364635 |
| *Mycobacteroides abscessus* | P3M63_21295 | CP119724.1 | MGSVIKKRRKRMSKKKHRKLLRRTRVQRRKLGK | 33 | CP119725.1:4162704-4162805 | 37364635 |
| *Mycobacteroides abscessus* | P3M63_06960 | CP119724.1 | MAITRGAMPYEHYEHAVEALAVPRETTDPYP | 31 | CP119725.1:1373455-1373550 | 37364635 |
| *Mycobacteroides abscessus* | P3M62_15930 | CP119724.1 | MSGTVNAAVDILLIVLSAALVVYLVVALLDPERF | 34 | CP119724.1:3225866-3225970 | 37364635 |
| *Mycobacteroides abscessus* | P3M62_19805 | CP119724.1 | MGSVIKKRRKRMSKKKHRKLLRRTRVQRRKLGK | 33 | CP119724.1:3992185-3992286 | 37364635 |
| *Pseudomonas aeruginosa* | AAK73286 | AY038186.1 | IDGSVFIDSTSVQPGDKVRVRVVDADEYDLWAELV | 35 | AY038186.1:1-108 | 11722728 |
| *Pseudomonas aeruginosa* | AAM27867 | AF498420.1 | EKDAMKELRKQEVESAGPTTIGDLIRAQMENQG | 33 | AF498420.1:1-104 | 12057956 |
| *Pseudomonas aeruginosa* | AAK68045 | AF331071.1 | ISIGTLTKDVRAVDLSMRLTL | 21 | AF331071.1:677-742 | 11598071 |
| *Pseudomonas aeruginosa* | AAN63495 | AF317511.1 | MKTATAP | 7 | AF317511.1:1-21 | 15855521 |
| *Pseudomonas aeruginosa* | CAE51023 | AJ585042.1 | MKTATAPLPPLRSVKVLDQLRERIRYLHYSLRTEQA | 36 | AJ585042.1:1-107 | 14749341 |
| *Pseudomonas aeruginosa* | ATC05_02080 | CP013245.1 | MFIDEVVLAGILTVGLMVAFFGGVGYFIWKDSHSRKG | 37 | CP013245.1:440066-440179 | 26823594 |
| *Pseudomonas aeruginosa* | ATC05_05350 | CP013245.1 | MESFFRSLKAERVYLTRYASHQEAKTDQFDYIGF | 34 | CP013245.1:1160545-1160649 | 26823594 |
| *Pseudomonas aeruginosa* | ATC05_05515 | CP013245.1 | MAKKSKRPNKAKSLVAQPLFRSRQEQAKKGKGSYRREASRNWEASFVMAA | 50 | CP013245.1:1193662-1193814 | 26823594 |
| *Pseudomonas aeruginosa* | ATC05_07405 | CP013245.1 | MARKTDWPTNEKTFRTKRDAEDWARRTEDEMVRGVYIQHCGSERM | 45 | CP013245.1:1592625-1592762 | 26823594 |
| *Pseudomonas aeruginosa* | ATC05_11840 | CP013245.1 | MKRLFLSFVALALLAGSIAACGQKGPLYLPDDEKAKKEHSKDRYGF | 46 | CP013245.1:2544001-2544141 | 26823594 |
| *Pseudomonas aeruginosa* | ATC05_20600 | CP013245.1 | MLAGELGISLRTLYRDIATLRQQGAHIEGGAGARRCCASGVNGKASPRSE | 50 | CP013245.1:4452169-4452321 | 26823594 |
| *Pseudomonas aeruginosa* | PA8380_05350 | AP014839.2 | MRRALIPIGIFLALGLLLILAGDALMLGRRLIAWQWGC | 38 | AP014839.2:570961-571077 | 25999558 |
| *Pseudomonas aeruginosa* | PA8380_06490 | AP014839.2 | MGRKTDWPTNEKTFRTKRDAEDWARRTEDEMVRGVYIQHCGSERM | 45 | AP014839.2:659707-659844 | 25999558 |
| *Pseudomonas aeruginosa* | PA8380_26500 | AP014839.2 | MLCSRTASRPAMFRRRAAARPQARRNREFLLATPADNGPTKT | 42 | AP014839.2:2722544-2722672 | 25999558 |
| *Pseudomonas aeruginosa* | PA8380_27350 | AP014839.2 | MWTYRERRNRAAFSNAQLAYDRAVDPLWDQPEPEPEHEDEEQEDDDGLGE | 50 | AP014839.2:2807567-2807719 | 25999558 |
| *Pseudomonas aeruginosa* | PA8380_34110 | AP014839.2 | MFNSTCRNRPAPPERIDPMWTKPSFTDLRLGFEVTLYFANR | 41 | AP014839.2:3593004-3593129 | 25999558 |
| *Pseudomonas aeruginosa* | PA8380_37890 | AP014839.2 | MTVLDWLSLALATGLFVYLLVALLRADRA | 29 | AP014839.2:3987686-3987775 | 25999558 |
| *Pseudomonas aeruginosa* | PA8380_44400 | AP014839.2 | MLKVIATDAGSQRDFRAFAQLAGHELLREEAAEGVYRYWLRKR | 43 | AP014839.2:4661508-4661639 | 25999558 |
| *Pseudomonas aeruginosa* | PA8380_59720 | AP014839.2 | MKRLFLSFVALALLAGSIAACGQKGPLYLPDDEKAKKEHSKDRYGF | 46 | AP014839.2:6288859-6288999 | 25999558 |
| *Streptomyces venezuelae* | SVEN_1907 | FR845719.1 | MLVSAGAEVPSASRHFLRNKKQEWEEYRSEVTAFELRKNLPVL | 43 | FR845719.1:2125884-2126015 | 21463507 |
| *Streptomyces venezuelae* | SVEN_4404 | FR845719.1 | MKVKPSVKKICDKCKVIRRHGRVMVICDNLRHKQRQG | 37 | FR845719.1:4748194-4748307 | 21463507 |
| *Streptomyces venezuelae* | SVEN_7454 | FR845719.1 | MVDEVGYQPLERAEANLVFQVISKRYEKGSSS | 32 | FR845719.1:8224386-8224484 | 21463507 |
| *Sinorhizobium meliloti* | WGR33_13725 | CP148086.1 | MKIRQKITEYVKMRRAVRELNALDDHALSDIGISRSQIQAAVYGR | 45 | CP148086.1:2798543-2798680 | 11274136 |
| *Sinorhizobium meliloti* | WGR33_03635 | CP148086.1 | MSKTVFAVLILIFASSCANTARGFREDGTQTGHAVDRATHRVLSAGAQ | 48 | CP148086.1:719740-719886 | 11274136 |
| *Sinorhizobium meliloti* | WGR33_06780 | CP148086.1 | MSRILVVMIALLSVAGLTACDTIGKGKGKAPPPAAVDTVEPAPVYK | 46 | CP148086.1:1348790-1348930 | 11274136 |
| *Sinorhizobium meliloti* | WGR33_07030 | CP148086.1 | MNLARSFNNWRKYRQTCNELGRMSDRELTDLGIGRADIPYVARQAIK | 47 | CP148086.1:1408786-1408929 | 11274136 |
| *Sinorhizobium meliloti* | WGR33_09485 | CP148086.1 | MFKQLKKITRALHIPSVEERETAYLNGSVDRIDLEYRQRQIDRGLFRKGF | 50 | CP148086.1:1912276-1912428 | 11274136 |
| *Sinorhizobium meliloti* | WGR33_01150 | CP148086.1 | MANTQNISIWWWAR | 14 | CP148086.1:235968-236012 | 11274136 |
| *Sinorhizobium meliloti* | WGR33_03100 | CP148086.1 | MCILIALALSILGFIPENEVPPDVPEVVPQTPLR | 34 | CP148086.1:628146-628250 | 11274136 |
| *Sinorhizobium meliloti* | CAA33182 | X15079.1 | GFGPS | 5 | X15079.1:1-18 | 2747618 |
| *Sinorhizobium meliloti* | CAA79903 | Z21854.1 | METYTAMRHFADSWGLLAMTLFFLGVVFFIFRPGAKNAAAQASVIPLKED | 50 | Z21854.1:7347-7499 | 2747618 |
| *Sinorhizobium meliloti* | CAA36889 | X52662.1 | EAYGHDYIESALGALYAAEFGIEPPVKTATA | 31 | X52662.1:1-96 | 2747618 |
| *Sinorhizobium meliloti* | AAA26254 | Z50189.1 | MLPDWACHVEDIM | 13 | M33555.1:726-764 | 2747618 |
| *Sinorhizobium meliloti* | CAA90567 | Z50189.1 | MQNNNILVVGGAGYIGSHTCLQLAAKGYQPVVYDNLSNGHEEF | 43 | Z50189.1:1-129 | 2747618 |
| *Sinorhizobium meliloti* | AAG42538 | AY013584.1 | MAWHKPKFIEVSCAMEITRYAPADGDEPILF | 31 | AY013584.1:1932-2027 | 2747618 |
| *Zymomonas mobilis* | AAA71938 | L09651.1 | MRVAIFSSKNYDHHSIEKENEHYGHDLV | 28 | L09651.1:1475-1560 | 8320209 |
| *Zymomonas mobilis* | AAA71936 | L09651.1 | DCLAILAEMIDQLPISGKDLA | 21 | L09651.1:1-67 | 8320209 |
| *Zymomonas mobilis* | AAA71935 | L09651.1 | MKAAVITKDHTIEVKDTKLRPLKYGEALLEMEYCGVCHTD | 40 | L09650.1:2225-2345 | 8320209 |
| *Zymomonas mobilis* | AAA27689 | M18802.1 | MAFRTLDDIGDVKGKRVLVREDLNVPMDGD | 30 | M18802.1:1623-1713 | 3680173 |
| *Zymomonas mobilis* | Zymop_0068 | EU327527.1 | MKIRNSLKSLKERHRDNRVIRRRGRTYIINKTVRRFKARQG | 41 | EU327527.1:1-19 | 3680173 |
| *Zymomonas mobilis* | Zymop_0121 | CP002865.1 | METTEYGLITLVEEGDIIHIDANKDTIDLDVPENVLAERRKN | 42 | CP002865.1:74572-74697 | 21742897 |
| *Zymomonas mobilis* | Zymop_1689 | CP002865.1 | MTSILITGAGTGFGQEIALRLASQGLSIIAGVEIPSQIYSL | 41 | CP002865.1:135986-136114 | 21742897 |
| *Zymomonas mobilis* | Zmob_0117 | CP002865.1 | MRILIHCLMASAVLALAACNTVQGFGQDLSSAGQSMSNSAERNK | 44 | CP002865.1:1973921-1974046 | 21742897 |
| *Zymomonas mobilis* | Zmob_0923 | CP002865.1 | MSVASNTVQTYSRVGIREDLSDIIYNISPTGVLVR | 35 | CP002850.1:125961-126095 | 21742897 |
| *Zymomonas mobilis* | ZZM4_0015 | CP002865.1 | MADLAAIFHWSPEQLFEMPVSELMDWRERAIKRFNQMYGGESGE | 44 | CP002850.1:1093798-1093905 | 21742897 |
| *Zymomonas mobilis* | ZMO1_ZMO1862 | CP001881.1 | MTMKPIDCLVFEDSDEGLEAARRAGMSAIDIRATKN | 36 | CP001881.1:8623-8757 | 29743953 |
| *Zymomonas mobilis* | ZMO1_ZMO2011 | CP023715.1 | MRILIHFLMASAVLALAACNTVQGFGQDLSSAGQSMSNSAERNK | 44 | CP023715.1:1244663-1244797 | 29743953 |
| *Salmonella enterica* | AASJ52_05975 | CP156168.1 | MKSYIYKSLTTLCSVLIVSSFIYVWVTTY | 29 | CP156168.1:1265676-1265765 | 35381053 |
| *Salmonella enterica* | WP_001082117 | - | MNRSPDKIIALIFLLISLLVLCLALWQIVF | 30 | - | 31649255 |
| *Salmonella enterica* | WP_348240947 | - | VVLGIVVVVLLLLLGQVFNIMCDQAVQFFSGIFAINKFIPW | 41 | - | 20041203 |
| *Salmonella enterica* | AASJ52_21210 | CP156168.1 | MKKFRWVVLGIVVVVCLLLWAQVFNIMCDQDVQFFSGICAINKFIPW | 47 | CP156168.1:4379067-4379210 | 35381053 |
| *Streptomyces* | WP_351714011 | - | VAGWPIAGCHNETLLEIITRRMRCIGRWLKDTLNQRGGPA | 40 | - | 17890311 |
| *Synechococcus* | WP_353292692 | - | MIEPLLCGIVLGLIPVTLLGLFVAAWNQYRRSESALGG | 38 | - | 14526088 |
| *Synechocystis* | C7I86_00760 | CP028094.1 | MDILTLGWVSVLVLFTWSISMVVWGRNGF | 29 | CP028094.1:160004..160093 | 25738257 |
| *Yersinia pestis* | SEP004654 | NC_005810 | LYLAYFCWLHKPPEPVNRQ | 19 | NC_005810:4233759-4233815 | 34728737 |
| *Yersinia pestis* | SEP022867 | NC_005810 | DRLLISNRANSFHRK | 15 | NC_005810:1795135:1795179 | 34728737 |
| *Yersinia pestis* | SEP030801 | NC_005810 | MGGIAWVSSLESKRFSVVRIP | 21 | NC_005810:186742-186742 | 34728737 |
| *Yersinia pestis* | SEP031477 | NC_005810 | VSKDKISIDAGQTICVAAGMPLAR | 24 | NC_005810:2603606-2603677 | 34728737 |
| *Yersinia pestis* | SEP042672 | NC_005810 | PEKCRFTGFDGLREILRDVKLERVKGIEPSYRAWEALVLPLNYTRVGVRLAL | 52 | NC_005810:4076357-4076512 | 34728737 |
| *Yersinia pestis* | SEP055098 | NC_005810 | QQTSRKTAAIAPDKF | 15 | NC_005816:8586-8630 | 34728737 |
| *Yersinia pestis* | SEP065022 | NC_005810 | SIVICRSWLYIVHSRMSLILVCRSSWYELVSSHALSDKKSLMDALCAQLAGIACG | 55 | NC_005810:3550631-3550795 | 34728737 |
| *Yersinia pestis* | SEP065582 | NC_005810 | SLVNLLSSLFPAWGDLYLLTVV | 22 | NC_005810:4591944-4592009 | 34728737 |
| *Yersinia pestis* | SEP121076 | NC_005810 | SVRARNSVAQAPREGVRLIDHLPALSAVGTWSVI | 34 | NC_005810:3883653-3883754 | 34728737 |
| *Yersinia pestis* | SEP141063 | NC_005810 | LTLLINSLFYPK | 12 | NC_005810:3827156-3827191 | 34728737 |
| *Yersinia pestis* | SEP147565 | NC_005810 | SIPHARYIYLKETISYVLVVSLLTWISDLLY-IDARQY | 39 | NC_005810:1667889-1668005 | 34728737 |
| *Yersinia pestis* | SEP157269 | NC_005810 | QDITAENKKSTAESLYLKQFELREGGK | 27 | NC_005810:2930540-2930620 | 34728737 |
| *Yersinia pestis* | SEP174055 | NC_005810: | KENIQQPLNFL | 11 | NC_005810:2636658-2636626 | 34728737 |
| *Yersinia pestis* | SEP175570 | NC_005810: | INLPEQQHQQCRLKNRQKNEFVNQ | 24 | NC_005810:1359486-1359557 | 34728737 |
| *Yersinia pestis* | SEP194249 | NC_005810: | PGYYADIVGHKGTLPHPPPLYAPPRVIVTQLTLQPVLWC | 39 | NC_005810:3408207-3408323 | 34728737 |
| *Yersinia pestis* | SEP197173 | NC_005810: | LSLNGNHAVIIKINTGVPGKYANVSVLLVHVSQFPLSDDVFFFIQK | 46 | NC_005810:2156967-2157104 | 34728737 |
| *Yersinia pestis* | SEP219854 | NC_005810: | RITLSQEDRSHIMTKNTATKVKSIKLVTFGKNTALAGAVPRTLSGQEAGRVLGFGCHHS | 59 | NC_005810:2051201-2051377 | 34728737 |
| *Yersinia pestis* | SEP226039 | NC_005810: | FCLSDSQFNKKDALISAFYHSPAY | 24 | NC_005810:411694-411765 | 34728737 |
| *Yersinia pestis* | SEP228497 | NC_005810: | QQGMPIYRY | 9 | NC_005813:52149-52123 | 34728737 |
| *Vibrio vulnificus* | *VV1_2164* | *AE016795.3* | *MWYFAWILGVLLACAFGIINALWLEHSEMMDKDSE* | *35* | *AE016795.3:2169435-2169542* | *14500463* |
| *Vibrio vulnificus* | *VV1_2041* | *AE016795.3* | *MTVWRWRKEGRLPPARAISTRIVGWERETIERWLEEQTTELPY* | *43* | *AE016795.3:2032945-2033076* | *14500463* |
| *Vibrio vulnificus* | *VV1_1004* | *AE016795.3* | *MATKRTFQPSVLKRKRTHGFRARMATKNGRKVINARRAKGRARLSK* | *46* | *AE016795.3:1008011-1008151* | *14500463* |
| *Vibrio vulnificus* | *VV2_0693* | *AE016796.2* | *MTGETMTKCIRRTFSAEFKLEAAQLVLDQNYTVVEAAKAMGVGKSTMDSG* | *50* | *AE016796.2:747108-747260* | *14500463* |
| *Vibrio vulnificus* | *VVMO6_02975* | *CP002469.1* | *MATKRTFQPSVLKRKRTHGFRARMATKNGRKVINARRAKGRARLSK* | *46* | *CP002469.1:3189563-3189703* | *21317338* |
| *Vibrio vulnificus* | *VVMO6_02945* | *CP002469.1* | *MDISRRHFLQTSLAFSALTLLPAVHFRVEHRFIMGGLSMR* | *40* | *CP002469.1:3154593-3154715* | *21317338* |
| *Vibrio vulnificus* | *VVM_04363* | *CP002469.1* | *MITQLYIAIELGYLSKEQGMVFVQEAKHISVMLAKLIKIRKGTVR* | *45* | *CP002469.1:2982872-2983009* | *21317338* |
| *Vibrio vulnificus* | *VVMO6_02734* | *CP002469.1* | *MKVRASVKKICRNCKVIKRNGVVRVICSEPKHKQRQG* | *37* | *CP002469.1:2942581-2942694* | *21317338* |
| *Vibrio vulnificus* | *VVMO6_02186* | *CP002469.1* | *MVMGILPNTSVRLIRRAPMGDPLQIEARGVSLAVREKIAEAIEVECQ* | *47* | *CP002469.1:2353407-2353550* | *21317338* |
| *Vibrio vulnificus* | *VVMO6_01995* | *CP002469.1* | *MLQEFNHNQKAKLALHNENVNAAELAWWRTWTSSWWANVYF* | *41* | *CP002469.1:2148063-2148188* | *21317338* |
| *Vibrio vulnificus* | *VVMO6_01670* | *CP002469.1* | *MIACGEPLKIEEFAKWLEQGSKTARVDELKREEITCREYQGFEIL* | *45* | *CP002469.1:1777089-1777226* | *21317338* |
| *Vibrio vulnificus* | *VVMO6_00729* | *CP002469.1* | *MKVLKSLKSAKARHPDCQIVRRRGRLYVICKSNPRFKAVQK* | *41* | *CP002469.1:825021-825146* | *21317338* |
| *Vibrio vulnificus* | *VVMO6_00639* | *CP002469.1* | *MKASVDFLLDEEGLSVVEYVVGAALLVLAIGTLFSGYDTKLNNKIDGALS* | *50* | *CP002469.1:724121-724273* | *21317338* |
| *Vibrio vulnificus* | *VVMO6_00521* | *CP002469.1* | *MEWGEQSPHRPFCSKKCQMIDFGEWADEENAIPGAPDMSDSDGWSEEQY* | *49* | *CP002469.1:580943-581092* | *21317338* |
| *Vibrio vulnificus* | *VVMO6_00482* | *CP002469.1* | *MPLKENRRHGGFLSQQFSFSTNSLFTALRQPIERISYLPTG* | *41* | *CP002469.1:535418-535543* | *21317338* |
| *Vibrio vulnificus* | *VVMO6_00101* | *CP002469.1* | *MKKILTVLLTLSVALLAGCGQSGALYIPDDAQQSEQSQ* | *38* | *CP002469.1:113137-113253* | *21317338* |
| *Vibrio vulnificus* | *VV93_v1c03670* | *CP009261.1* | *MKVRASVKKICRNCKVIKRNGVVRVICSEPKHKQRQG* | *37* | *CP009261.1:393984-394097* | *25278541* |
| *Vibrio vulnificus* | *VV1_2959* | *AE016795.3* | *MQHNALVLRSKTVELVYQELWGLLLGYNLVRREASQAAVAHGRNGQ* | *46* | *AE016795.3:3022187-3022327* | *14500463* |
| *Vibrio vulnificus* | *VV93_v1c04640* | *CP009261.1* | *MLNHFLVRLTIGTLVVLGFKLSAIYFLLLILLLNTHHKEFFGW* | *43* | *CP009261.1:485700-485831* | *25278541* |
| *Vibrio vulnificus* | *VV93_v1c08710* | *CP009261.1* | *MDFWLDLLFGNAVGLSSMIVIFGALGLMLFYGGFIVYKVMNDKSPH* | *46* | *CP009261.1:940115-940255* | *25278541* |
| *Vibrio vulnificus* | *VV93_v1c11400* | *CP009261.1* | *MLQEFNHNQKAKLALHNENVNAAELAWWRTWTSSWWANVYF* | *41* | *CP009261.1:1237827-1237952* | *25278541* |
| *Vibrio vulnificus* | *VV93_v1c21200* | *CP009261.1* | *MADKDFKEPYNLFYFLGFIAVLLIPTLPATLTWIRVLNGYAGF* | *43* | *CP009261.1:2331658-2331789* | *25278541* |
| *Vibrio vulnificus* | *VV93_v1c30080* | *CP009261.1* | *MATKRTFQPSVLKRKRTHGFRARMATKNGRKVINARRAKGRARLSK* | *46* | *CP009261.1:3313043-3313183* | *25278541* |
| *Vibrio vulnificus* | *FORC17_0450* | *CP012739.1* | *MLLLCLKESNQLIRIEEALAGRAPFNGLKEVKGQA* | *35* | *CP012739.1:528752-528859* | *27325916* |
| *Vibrio vulnificus* | *FORC17_0482* | *CP012739.1* | *MEWGEQSPHRPFCSKKCQMIDFGEWADEENAIPGAPDMSDSDGWSEEQY* | *49* | *CP012739.1:563291-563440* | *27325916* |
| *Vibrio vulnificus* | *FORC17_0598* | *CP012739.1* | *MKASVDFLLDEEGLSVVEYVVGAALLVLAIGTLFSGYDTKLNNKIDGALS* | *50* | *CP012739.1:706431-706583* | *27325916* |
| *Vibrio vulnificus* | *FORC17_0685* | *CP012739.1* | *MKVLKSLKSAKARHPDCQIVRRRGRLYVICKSNPRFKAVQK* | *41* | *CP012739.1:807626-807751* | *27325916* |
| *Vibrio vulnificus* | *FORC17_1001* | *CP012739.1* | *MGAWLRGLFARRGKAKAIIALANKIGRVVWCVISTGCEYDVKLAFKPL* | *48* | *CP012739.1:1145700-1145846* | *27325916* |
| *Vibrio vulnificus* | *FORC17_1446* | *CP012739.1* | *MSIWKNLYNKAFKRDSCRVAFWVCSEFGGESGLRKLGLCGTHPLTQRYV* | *49* | *CP012739.1:1586035-1586184* | *27325916* |
| *Vibrio vulnificus* | *FORC17_1666* | *CP012739.1* | *MIACGEPLKIEEFAKWLEQGPKTARVDELKREEITCREYQGFEIL* | *45* | *CP012739.1:1821824-1821961* | *27325916* |
| *Vibrio vulnificus* | *FORC17_2160* | *CP012739.1* | *MVMGILPNTSVRLIRRAPMGDPLQIEARGVSLAVREKIAEAIEVECQ* | *47* | *CP012739.1:2388889-2389032* | *27325916* |
| *Vibrio vulnificus* | *FORC17_1001* | *CP012739.1* | *MGAWLRGLFARRGKAKAIIALANKIGRVVWCVISTGCEYDVKLAFKPL* | *48* | *CP012739.1:1145700-1145846* | *27325916* |
| *Vibrio vulnificus* | *Y702_00685* | *JDSE01000001.1* | *MLNHFLVRLTIGTLVVLGFKLSAIYFLLLILLLNTHHKEFFGW* | *43* | *JDSE01000001.1:125690-125821* | *24652973* |
| *Vibrio vulnificus* | *Y702_06610* | *JDSE01000013.1* | *MATKRTFQPSVLKRKRTHGFRARMATKNGRKVINARRAKGRARLSK* | *46* | *JDSE01000013.1:52222-52362* | *24652973* |
| *Vibrio vulnificus* | *Y702_08305* | *JDSE01000018.1* | *MLQEFNHNQKAKLVLHNENVNAAELAWWRTWTSSWWANVYF* | *41* | *JDSE01000018.1:37902-38027* | *24652973* |
| *Vibrio vulnificus* | *Y702_15745* | *JDSE01000047.1* | *RNNVYFKSKRDFKVAIDHFFAVTLPEIAGSLTSRINDNFQVLKPASSS* | *48* | *JDSE01000047.1:1-147* | *24652973* |
| *Vibrio vulnificus* | *Y702_19200* | *JDSE01000066.1* | *MKCSDFMCDEEGLSVVEYVVGAALLVLAIGTLFSGYDTKLNNKIDGALS* | *49* | *JDSE01000066.1:28792-28941* | *24652973* |
| *Vibrio vulnificus* | *Y702_21475* | *JDSE01000088.1* | *MDYIRANTGRLFDPECVGALIDNLEQFLAIRDRYLDQEELPKGCVA* | *46* | *JDSE01000088.1:11128-11268* | *24652973* |
| *Vibrio vulnificus* | *Y702_23385* | *JDSE01000116.1* | *MAKFLKVSRTSVNKWVQTFLEERLEGLKEKPRTGRPAFLNAEQREQL* | *47* | *JDSE01000116.1:1-142* | *24652973* |
| *Vibrio vulnificus* | *LO82_20025* | *LIIO01000047.1* | *MKVRASVKKICRNCKVIKRNGVVRVICSEPKHKQRQG* | *37* | *LIIO01000047.1:19645-19758* | *26472833* |
| *Vibrio vulnificus* | *LO82_08085* | *LIIO01000017.1* | *MLQEFNHNQKAKLALHNENVNAAELAWWRTWTSSWWANVYF* | *41* | *LIIO01000017.1:142903-143028* | *26472833* |
| *Vibrio vulnificus* | *LO82_05345* | *LIIO01000010.1* | *MKVLKSLKSAKARHPDCQIVRRRGRLYVICKSNPRFKAVQK* | *41* | *LIIO01000010.1:236371-236496* | *26472833* |
| *Vibrio vulnificus* | *VVYB158_23670* | *LBNN01000076.1* | *MATKRTFQPSVLKRKRTHGFRARMATKNGRKVINARRAKGRARLSK* | *46* | *LBNN01000076.1:52368-52508* | *26205875* |
| *Vibrio vulnificus* | *VVYB158_23165* | *LBNN01000075.1* | *MSKEKFERVKPHVNVGTIGHVDHGKTTL* | *28* | *LBNN01000075.1:1-84* | *26205875* |
| *Vibrio vulnificus* | *VVYB158_20890* | *LBNN01000021.1* | *MKLNKATLLPMLATAVVTIGIITVINNVSQLATVKDTVNGNKGWF* | *45* | *LBNN01000021.1:89875-90012* | *26205875* |
| *Vibrio vulnificus* | *VVYB158_19650* | *LBNN01000019.1* | *MLIAIKLIKLAVICAIFFTIYDLIAFGEITWFTRFFGL* | *38* | *LBNN01000019.1:293219-293335* | *26205875* |
| *Vibrio vulnificus* | *VVYB158_04880* | *LBNN01000008.1* | *MLQEFNHNQKAKLALHNENVNAAELAWWRTWTSSWWANVYF* | *41* | *LBNN01000008.1:278837-278962* | *26205875* |
| *Vibrio natriegens* | *M272_22630* | *ATFJ01000001.1* | *MWYFAWILGVLLACAFGIINALWLEHSEMMDKDSE* | *35* | *ATFJ01000001.1:155720-155827* | *23929482* |
| *Vibrio natriegens* | *M272_02970* | *ATFJ01000003.1* | *MDNDQLWNFLLTPSGIMVSMIITFGVVGIYAYLTGQFKD* | *39* | *ATFJ01000003.1:185436-185555* | *23929482* |
| *Vibrio natriegens* | *M272_06535* | *ATFJ01000009.1* | *MKRTFQPTVLKRKRTHGFRARMATKNGRKVINARRAKGRARLSK* | *44* | *ATFJ01000009.1:52640-52774* | *23929482* |
| *Vibrio natriegens* | *M272_08175* | *ATFJ01000012.1* | *MFIVYRLAKLAIICMIFFTIYDLIAYGEVTWFHRLLSYFN* | *40* | *ATFJ01000012.1:92766-92888* | *23929482* |
| *Vibrio natriegens* | *M272_08870* | *ATFJ01000013.1* | *MGCCNSDKSCQNEKKTKRTPWLGIVVGVLVVLVILNWR* | *38* | *ATFJ01000013.1:44116-44232* | *23929482* |
| *Vibrio natriegens* | *M272_01215* | *ATFJ01000017.1* | *MAKKSNKQLEKEKKESEQNNAAIKQKTRRRIEDIMEQREFDKLFDL* | *46* | *ATFJ01000017.1:62247-62387* | *23929482* |
| *Vibrio natriegens* | *M272_11830* | *ATFJ01000024.1* | *MADKEFKEPYNILYFLGFIAVLMIPTLPATLTWLRVFNGYAG* | *42* | *ATFJ01000024.1:51368-51496* | *23929482* |
| *Vibrio natriegens* | *M272_14590* | *ATFJ01000033.1* | *MEEDEKAIMMLAVIVGFVLIWVFLEEVNYFGFR* | *33* | *ATFJ01000033.1:78794-78895* | *23929482* |
| *Vibrio natriegens* | *M272_03800* | *ATFJ01000035.1* | *MKVVKSLKSAKSRHPDCQIVKRRGRLYVICKTNPRFKAVQK* | *41* | *ATFJ01000035.1:56989-57114* | *23929482* |
| *Vibrio natriegens* | *M272_17425* | *ATFJ01000037.1* | *MLQELKQNQKAKVAVCLNKTSSADVAWWRTWTSSWWANVYF* | *41* | *ATFJ01000037.1:106147-106272* | *23929482* |
| *Vibrio natriegens* | *M272_18995* | *ATFJ01000038.1* | *MKVRASVKKICRNCKVIKRNGVVRVICSEPKHKQRQG* | *37* | *ATFJ01000038.1:160200-160313* | *23929482* |
| *Synechocystis* | *C7I86_00760* | *CP028094.1* | *MDILTLGWVSVLVLFTWSISMVVWGRNGF* | *29* | *CP028094.1:160004..160093* | *25738257* |
| *Synechocystis* | *MT986_08655* | *CP094998.1* | *MIEPLLLGIVLGLIPVTLAGLFVAAYLQYKRGNQFNLD* | *38* | *CP094998.1:1822388-1822504* | *36792971* |
| *Synechocystis* | *MT986_08680* | *CP094998.1* | *MTQRTLGGTNRKQKRTSGFRARMRTHNGRKVIQARRSKGRHRLAV* | *45* | *CP094998.1:1825582-1825719* | *36792971* |
| *Synechocystis* | *MT986_10170* | *CP094998.1* | *MTIEEVLDDFPDLTPEDIQACFAFAADRDRRLMVVPDEIAV* | *41* | *CP094998.1:2136137-2136262* | *36792971* |
| *Synechocystis* | *MT986_11090* | *CP094998.1* | *MLTLKIAVYIVVGLFISLFIFGFLSSDPTRNPGRKDFE* | *38* | *CP094998.1:2347780-2347896* | *36792971* |
| *Synechocystis* | *MT986_11335* | *CP094998.1* | *MESVAYILVLTMALAVLFFAIAFREPPRIEK* | *31* | *CP094998.1:2412226-2412321* | *36792971* |
| *Synechocystis* | *MT986_11695* | *CP094998.1* | *MHPIAVHLPVGMEKCSNLFSFEDIVGGGDLDYNDVILEFFPVI* | *43* | *CP094998.1:2487526-2487657* | *36792971* |
| *Synechocystis* | *MT986_12205* | *CP094998.1* | *MAHRGNKAHLPSKICPVCQRPFTWRKKWANCWDEVKYCSDRCRRSRIGSP* | *50* | *CP094998.1:2592754-2592906* | *36792971* |
| *Synechocystis* | *MT986_12290* | *CP094998.1* | *MTPSLANFLWSLVLGAAIVLIPATVGLIFISQKDKITRS* | *39* | *CP094998.1:2611123-2611242* | *36792971* |
| *Synechocystis* | *MT986_14630* | *CP094998.1* | *MTAESMLANGAFIMIGLTLLGLAWGFVIIKLQGSEE* | *36* | *CP094998.1:3115834-3115944* | *36792971* |
| *Synechocystis* | *MT986_16070* | *CP094998.1* | *MDGSYAASYLPWILIPMVGWLFPAVTMGLLFIHIESEGEG* | *40* | *CP094998.1:3454482-3454604* | *36792971* |
| *Synechocystis* | *MT986_00680* | *CP094998.1* | *MQVNNLGFIASILFVLVPTVFLLILFIQTGKQSES* | *35* | *CP094998.1:146724-146831* | *36792971* |
| *Synechocystis* | *MT986_00765* | *CP094998.1* | *MDILTLGWVSVLVLFTWSISMVVWGRNGF* | *29* | *CP094998.1:160004-160093* | *36792971* |
| *Synechocystis* | *MT986_02245* | *CP094998.1* | *MALSDTQILAALVVALLPAFLAFRLSTELYK* | *31* | *CP094998.1:467201-467296* | *36792971* |
| *Synechocystis* | *MT986_02595* | *CP094998.1* | *METIYLLAKLPEAYQIFDPLVDVLPVIPLFFLALAFVWQAAVGFK* | *45* | *CP094998.1:553065-553202* | *36792971* |
| *Synechocystis* | *MT986_02690* | *CP094998.1* | *MATQNPNQPVTYPIFTVRWLAVHTLAVPSVFFVGAIAAMQFIQR* | *44* | *CP094998.1:570940-571074* | *36792971* |
| *Synechocystis* | *MT986_02695* | *CP094998.1* | *MDRNSNPNRQPVELNRTSLYLGLLLVAVLGILFSSYFFN* | *39* | *CP094998.1:571084-571203* | *36792971* |
| *Synechocystis* | *MT986_03915* | *CP094998.1* | *MKVRASVKKMCDKCRVIRRRGRVMVICSANPKHKQRQG* | *38* | *CP094998.1:831102-831218* | *36792971* |
| *Synechocystis* | *MT986_05335* | *CP094998.1* | *MNNENSKFGFTAFAENWNGRLAMIGFSSALILELVSGQGVLHFFGIL* | *47* | *CP094998.1:1141804-1141947* | *36792971* |
| *Synechocystis* | *MT986_05485* | *CP094998.1* | *MESATVLSITFAVILIAITGLAVYTSFGPPSAELGDPFDDHED* | *43* | *CP094998.1:1167334-1167465* | *36792971* |
| *Synechocystis* | *MT986_05980* | *CP094998.1* | *MDWRVIVVVSPLLIAATWAAINIGAAAIRQLQDVLGREA* | *39* | *CP094998.1:1267007-1267126* | *36792971* |
| *Synechocystis* | *MT986_07775* | *CP094998.1* | *MAAGVGIFIGYIAVFTGVTLGLLYGLRFVKLI* | *32* | *CP094998.1:1642320-1642418* | *36792971* |
| *Synechocystis* | *MT986_07995* | *CP094998.1* | *MDGLKSFLSTAPVMIMALLTFTAGILIEFNRFYPDLLFHP* | *40* | *CP094998.1:1686144-1686266* | *36792971* |
| *Synechocystis* | *MT986_14715* | *CP094998.1* | *MELLAALNLEPIFQLTFLGLIVLAGPAVVFVLAFRGGDL* | *39* | *CP094998.1:3137687-3137806* | *36792971* |
| *Synechocystis* | *AOY38_00670* | *CP012832.1* | *MQVNNLGFIASILFVLVPTVFLLILFIQTGKQSES* | *35* | *CP012832.1:146724-146831* | *26473841* |
| *Synechocystis* | *AOY38_02215* | *CP012832.1* | *MALSDTQILAALVVALLPAFLAFRLSTELYK* | *31* | *CP012832.1:467303-467398* | *26473841* |
| *Synechocystis* | *AOY38_02565* | *CP012832.1* | *METIYLLAKLPEAYQIFDPLVDVLPVIPLFFLALAFVWQAAVGFK* | *45* | *CP012832.1:553167-553304* | *26473841* |
| *Synechocystis* | *AOY38_02660* | *CP012832.1* | *MATQNPNQPVTYPIFTVRWLAVHTLAVPSVFFVGAIAAMQFIQR* | *44* | *CP012832.1:571042-571176* | *26473841* |
| *Synechocystis* | *AOY38_02665* | *CP012832.1* | *MDRNSNPNRQPVELNRTSLYLGLLLVAVLGILFSSYFFN* | *39* | *CP012832.1:571186-571305* | *26473841* |
| *Synechocystis* | *AOY38_04230* | *CP012832.1* | *MDYLEKLLDKLRELAQKLIEGLLGPQGEPEPELIPIPVNDRRSRR* | *45* | *CP012832.1:903883-904020* | *26473841* |
| *Synechocystis* | *AOY38_05465* | *CP012832.1* | *MESATVLSITFAVILIAITGLAVYTSFGPPSAELGDPFDDHED* | *43* | *CP012832.1:1167589-1167720* | *26473841* |
| *Synechocystis* | *AOY38_05930* | *CP012832.1* | *MDWRVIVVVSPLLIAATWAAINIGAAAIRQLQDVLGREA* | *39* | *CP012832.1:1267262-1267381* | *26473841* |
| *Synechocystis* | *AOY38_06180* | *CP012832.1* | *MESMENTANLDWILGAIALGILVAGLVMLLNGVKDLGK* | *38* | *CP012832.1:1319717-1319833* | *26473841* |
| *Synechocystis* | *AOY38_07730* | *CP012832.1* | *MAAGVGIFIGYIAVFTGVTLGLLYGLRFVKLI* | *32* | *CP012832.1:1642575-1642673* | *26473841* |
| *Synechocystis* | *AOY38_08620* | *CP012832.1* | *MIEPLLLGIVLGLIPVTLAGLFVAAYLQYKRGNQFNLD* | *38* | *CP012832.1:1822644-1822760* | *26473841* |
| *Synechocystis* | *AOY38_08735* | *CP012832.1* | *MSDLNRGIMKFDGADKPFLVAVSAMLILGGIGALIIWALRVAYAVG* | *46* | *CP012832.1:1841790-1841930* | *26473841* |
| *Synechocystis* | *AOY38_11035* | *CP012832.1* | *MLTLKIAVYIVVGLFISLFIFGFLSSDPTRNPGRKDFE* | *38* | *CP012832.1:2347049-2347165* | *26473841* |
| *Synechocystis* | *AOY38_11275* | *CP012832.1* | *MESVAYILVLTMALAVLFFAIAFREPPRIEK* | *31* | *CP012832.1:2411494-2411589* | *26473841* |
| *Synechocystis* | *AOY38_12200* | *CP012832.1* | *MTPSLANFLWSLVLGAAIVLIPATVGLIFISQKDKITRS* | *39* | *CP012832.1:2610391-2610510* | *26473841* |
| *Synechocystis* | *AOY38_14495* | *CP012832.1* | *MTAESMLANGAFIMIGLTLLGLAWGFVIIKLQGSEE* | *36* | *CP012832.1:3115102-3115212* | *26473841* |
| *Synechocystis* | *AOY38_15940* | *CP012832.1* | *MDGSYAASYLPWILIPMVGWLFPAVTMGLLFIHIESEGEG* | *40* | *CP012832.1:3453749-3453871* | *26473841* |
| *Synechocystis* | *D082_00600* | *CP007542.1* | *MAKTAFAKGITLKQALVDLGLLTPAEFDAWVVPEQMITPIAN* | *42* | *CP007542.1:62845-62973* | *25081267* |
| *Synechocystis* | *D082_01060* | *CP007542.1* | *MTQRTLGGTNRKQKRTSGFRARMRTHNGRKVIQARRSKGRHRLAV* | *45* | *CP007542.1:102975-103112* | *25081267* |
| *Synechocystis* | *D082_01710* | *CP007542.1* | *MLGLGTALSWFHWVTASGGNGEKLVQSQYCPATVMGPAP* | *39* | *CP007542.1:167627-167746* | *25081267* |
| *Synechocystis* | *D082_01760* | *CP007542.1* | *MDWRVIVVVSPLLIAATWAAINIGAAAIRQLQDVLGREA* | *39* | *CP007542.1：172306-172425* | *25081267* |
| *Synechocystis* | *D082_04270* | *CP007542.1* | *MLTLKIAVYIVVGLFISLFIFGFLSSDPTRNPGRKDFE* | *38* | *CP007542.1:408602-408718* | *25081267* |
| *Synechocystis* | *D082_05630* | *CP007542.1* | *MLITVTQYRTHNIGYSGMVLLHGVGQAIWGIFQQSSQPQPE* | *41* | *CP007542.1:533119-533244* | *25081267* |
| *Synechocystis* | *D082_09450* | *CP007542.1* | *MTPSLANFLWSLVLGASIVVIPATIGLIFISQKDKITRS* | *39* | *CP007542.1:948725-948844* | *25081267* |
| *Synechocystis* | *D082_15990* | *CP007542.1* | *MNWYRRLSKDYEHLVEMSEAAIYAVMTRIMLRRLSSSSPSFTL* | *43* | *CP007542.1:1597205-1597336* | *25081267* |
| *Synechocystis* | *D082_18590* | *CP007542.1* | *MIEPLLLGIVLGLIPVTLAGLFVAAYLQYKRGNQFNLD* | *38* | *CP007542.1:1839574-1839690* | *25081267* |
| *Synechocystis* | *D082_23720* | *CP007542.1* | *MFAEGRIPLWVVGVVAGIGAIGVLGLFFYGAYAGLGSSM* | *39* | *CP007542.1:2342742-2342861* | *25081267* |
| *Synechocystis* | *D082_23740* | *CP007542.1* | *MATQNPNQPVTYPIFTVRWLAVHTLAVPSVFFVGAIAAMQFIQR* | *44* | *CP007542.1:2343035-2343169* | *25081267* |
| *Synechocystis* | *D082_25415* | *CP007542.1* | *MALSDTQVLAALVVALLPAFLAFRLSTELYK* | *31* | *CP007542.1:2500271-2500366* | *25081267* |
| *Synechocystis* | *D082_25740* | *CP007542.1* | *MGRSAYLCPTHDCLRKARQKNTLGRALRAPVPDHLFEQLEARLLATP* | *47* | *CP007542.1:2545154-2545297* | *25081267* |
| *Synechocystis* | *D082_26460* | *CP007542.1* | *MESATVLSITFAVILIAITGLAVYTSFGPPSAELGDPFDDHED* | *43* | *CP007542.1:2622744-2622875* | *25081267* |
| *Synechocystis* | *D082_33150* | *CP007542.1* | *MYRLDCKNKFLGIFEIMESVAYILVLTMALAVLFFAIAFREPPRIEK* | *47* | *CP007542.1:3297708-3297851* | *25081267* |
| *Synechocystis* | *D082_34500* | *CP007542.1* | *MLAINKDLRHTLPAIRPLRHCHPCVIIAVNAIFLELYPLAFQQR* | *44* | *CP007542.1:3424086-3424220* | *25081267* |
| *Synechocystis* | *D082_34840* | *CP007542.1* | *MLAKAFAGSKSHKILLNAWFTALPRQCQKPIVQFVLSSAAI* | *41* | *CP007542.1:3452776-3452901* | *25081267* |
| *Streptomyces tsukubensis* | *STSU_001150* | *CP029159.1* | *MALLDLQALEITEDEAFGDVETGSNLSLTSCGHNSHLSLLAC* | *42* | *CP029159.1:288085-288213* | *22740677* |
| *Streptomyces tsukubensis* | *STSU_004685* | *CP029159.1* | *MTTENVVGLIVAVALLGYLVLALLYPERF* | *29* | *CP029159.1:1236758-1236847* | *22740677* |
| *Streptomyces tsukubensis* | *STSU_013325* | *CP029159.1* | *MKVKPSVKKICDKCKVIRRHGRVMVICDNLRHKQRQG* | *37* | *CP029159.1:3267571-3267684* | *22740677* |
| *Streptomyces tsukubensis* | *STSU_014505* | *CP029159.1* | *MGSVIKKRRKRMAKKKHRKLLKRTRVQRRNKK* | *32* | *CP029159.1:3512891-3512989* | *22740677* |
| *Streptomyces tsukubensis* | *STSU_027275* | *CP029159.1* | *MRALVAAAIGLAAAFALVLTVTAIGAPPGETSPRPLLTTVPGPKK* | *45* | *CP029159.1:6393274-6393411* | *22740677* |
| *Streptomyces tsukubensis* | *STSU_004310* | *CP029159.1* | *MWIRLFAYLVAGHVFAGFLYLLFEVGAK* | *28* | *CP029159.1:1143739-1143825* | *22740677* |
| *Streptomyces tsukubensis* | *STSU_017700* | *CP029159.1* | *MSKRTFQPNNRRRAKTHGFRLRMRTRAGRAILASRRSKGRASLSA* | *45* | *CP029159.1:4215191-4215328* | *22740677* |
| *Streptococcus pneumoniae* | *CAA36857* | *X52632.1* | *MLVFQMRNVDKTSTVLKQTKNSDYADK* | *27* | *X52632.1:117-200* | *7934882* |
| *Streptococcus pneumoniae* | *J4Q31_06075* | *CP071918.1* | *MRVKINLKCSSCDSINYLTSKNSKTHPDKIEVLKYCPKERKVTLHLESK* | *49* | *CP071918.1:1178211-1178360* | *34982234* |
| *Streptococcus pneumoniae* | *J4Q31_07040* | *CP071918.1* | *MAYSTDFKQRALDYIKEGHSHVEAAKFFGVGVRTLFTWEKKDVNKDT* | *47* | *CP071918.1:1366576-1366719* | *34982234* |
| *Streptococcus pneumoniae* | *J4Q31_07915* | *CP071918.1* | *MELQELVERSWAIRQAYHELEVKHHDFKWTVEEDLLALSNDIGNFQ* | *46* | *CP071918.1:1532143-1532283* | *34982234* |
| *Streptococcus pneumoniae* | *J4Q31_08860* | *CP071918.1* | *MKANYPIYRPLTKRKFRVSDPYIYPHKFAVLDPNGYFLRFSE* | *42* | *CP071918.1:1717696-1717824* | *34982234* |
| *Streptococcus pneumoniae* | *J4Q31_08965* | *CP071918.1* | *MNYLFQEDSSSQIFNLGTKKGYTIKEIFNLGTKKGYTIKEIFKTAEELLN* | *50* | *CP071918.1:1737529-1737681* | *34982234* |
| *Streptococcus pneumoniae* | *J4Q31_09520* | *CP071918.1* | *MLAGVAIATPALNPPYLTGGYKSTGNSQKNIRKSFGYKGFTKFQRMSNGL* | *50* | *CP071918.1:1827756-1827908* | *34982234* |
| *Streptococcus pneumoniae* | *J4Q31_09585* | *CP071918.1* | *MILKYPENICMLCMPMVMHKNPSDKSIYHWDFYALLGF* | *38* | *CP071918.1:1840127-1840243* | *34982234* |
| *Streptococcus pneumoniae* | *J4Q31_09835* | *CP071918.1* | *MKRTYQPSKLRRARKHGFRNRMSTKNGRRVLAARRRKGRKVLAA* | *44* | *CP071918.1:1890666-1890800* | *34982234* |
| *Streptococcus pneumoniae* | *J4Q31_10995* | *CP071918.1* | *MRVNITLEHKESGERLYLTSKNKRNTPDRLQLKKYSPKLRKHVVFTEVK* | *49* | *CP071918.1:2079763-2079912* | *34982234* |
| *Streptococcus pneumoniae* | *J4Q31_11195* | *CP071918.1* | *MKQRKELYLFLGRTALYFLIFLGLLYFFSYLGQGQGSFIYNEF* | *43* | *CP071918.1:2128444-2128575* | *34982234* |
| *Streptococcus pneumoniae* | *J4Q31_11495* | *CP071918.1* | *MKNTVKLEQFVALKEKDLQKIKGGEMRLSKFFRDFILQRKK* | *41* | *CP071918.1:2192529-2192654* | *34982234* |
| *Streptococcus pneumoniae* | *J4Q31_01110* | *CP071918.1* | *MMKDLNNYREISNKELQEIKGGFGVGVGIALFMAGYTIGKDLRKKFGKSC* | *50* | *CP071918.1:189681-189833* | *34982234* |
| *Streptococcus pneumoniae* | *J4Q31_01480* | *CP071918.1* | *MVKRTCGYLGNPQARPMVNGRHKEIAARVKHMNGSTIKIAGHQVTN* | *46* | *CP071918.1:258419-258559* | *34982234* |
| *Streptococcus pneumoniae* | *J4Q31_01625* | *CP071918.1* | *MKVRPSVKPICEYCKVIRRNGRVMVICPANPKHKQRQG* | *38* | *CP071918.1:273241-273357* | *34982234* |
| *Streptococcus pneumoniae* | *J4Q31_01750* | *CP071918.1* | *MMELVLKTIIGPIVVGVVLRIVDKWLNKDK* | *30* | *CP071918.1:297888-297980* | *34982234* |
| *Streptococcus pneumoniae* | *J4Q31_02690* | *CP071918.1* | *MPDIVFEIEFFHSDSLIFFYTSDDKSNSQKSQEDFSKNK* | *39* | *CP071918.1:497037-497156* | *34982234* |
| *Streptococcus pneumoniae* | *J4Q31_03215* | *CP071918.1* | *MKEIAFDAFYQLYQNDQLSLVDVREVDEFAALHLECAHNLPLSQLADSYD* | *50* | *CP071918.1:599662-599814* | *34982234* |
| *Streptococcus pneumoniae* | *J4Q31_03220* | *CP071918.1* | *MRSARACQFLLEQGYNVINVQGGMLAFEEL* | *30* | *CP071918.1:599857-599949* | *34982234* |
| *Streptococcus pneumoniae* | *J4Q31_03570* | *CP071918.1* | *MSYKTSNAEGHVDFINTYDLEPMAQQVIPKAAFGYIASGAEDTFTSFQ* | *48* | *CP071918.1:678557-678703* | *34982234* |
| *Streptococcus pneumoniae* | *J4Q31_04095* | *CP071918.1* | *MANDNKSHYLIYRVLGISFEEGENIDLYQNKGRFLYKYAGSFLE* | *44* | *CP071918.1:779357-779491* | *34982234* |
| *Streptococcus pneumoniae* | *J4Q31_05055* | *CP071918.1* | *MTCDFKFETLQLHAGQVVAPATKSRAVPIYQTTFFVFDDT* | *40* | *CP071918.1:963911-964033* | *34982234* |
| *Streptococcus pneumoniae* | *J4Q31_05595* | *CP071918.1* | *MAFGDNGNRKKTMFEKITLFIVIIMLVASLLGIFATAIGALSNL* | *44* | *CP071918.1:1083150-1083284* | *34982234* |
| *Streptococcus pneumoniae* | *J4Q31_01795* | *CP071918.1* | *MDRTDEVSNHTYGKATLTWFEEIFEEYNTNLEYKQPICSQEKGLDN* | *46* | *CP071918.1:308104-308244* | *34982234* |
| *Streptococcus pneumoniae* | *J4Q31_03110* | *CP071918.1* | *MRKEFHNVLSSGQLLTDKRPARDYNRK* | *27* | *CP071918.1:576581-576664* | *34982234* |
| *Streptococcus pneumoniae* | *J4Q31_05355* | *CP071918.1* | *MPLKTITDRVGHSDSEVTTSIYTHVTKNMKDEAINVLDKVMKKIF* | *45* | *CP071918.1:1032898-1033035* | *34982234* |
| *Streptococcus pneumoniae* | *J4Q31_09535* | *CP071918.1* | *MLVFQMRNVDKTSTVLKQTKNSDYADK* | *27* | *CP071918.1:1829399-1829482* | *34982234* |
| *Streptococcus pneumoniae* | *J4Q30_07045* | *CP071917.1* | *MAYSTDFKQRALDYIKEGHSHVEAAKFFGVGVRTLFTWEKKDVNKDT* | *47* | *CP071917.1:1366563-1366706* | *34982234* |
| *Streptococcus pneumoniae* | *J4Q30_07920* | *CP071917.1* | *MELQELVERSWAIRQAYHELEVKHHDFKWTVEEDLLALSNDIGNFQ* | *46* | *CP071917.1:1532130-1532270* | *34982234* |
| *Streptococcus pneumoniae* | *J4Q30_08970* | *CP071917.1* | *MNYLFQEDSSSQIFNLGTKKGYTIKEIFNLGTKKGYTIKEIFKTAEELLN* | *50* | *CP071917.1:1737516-1737668* | *34982234* |
| *Streptococcus pneumoniae* | *J4Q30_09525* | *CP071917.1* | *MLAGVAIATPALNPPYLTGGYKSTGNSQKNIRKSFGYKGFTKFQRMSNGL* | *50* | *CP071917.1:1827756-1827908* | *34982234* |
| *Streptococcus pneumoniae* | *J4Q30_09590* | *CP071917.1* | *MILKYPENICMLCMPMVMHKNPSDKSIYHWDFYALLGF* | *38* | *CP071917.1:1840127-1840243* | *34982234* |
| *Streptococcus pneumoniae* | *J4Q30_11200* | *CP071917.1* | *MKQRKELYLFLGRTALYFLIFLGLLYFFSYLGQGQGSFIYNEF* | *43* | *CP071917.1:2129302-2129433* | *34982234* |
| *Streptococcus pneumoniae* | *J4Q30_11500* | *CP071917.1* | *MKNTVKLEQFVALKEKDLQKIKGGEMRLSKFFRDFILQRKK* | *41* | *CP071917.1:2193387-2193512* | *34982234* |
| *Streptococcus pneumoniae* | *J4Q30_01110* | *CP071917.1* | *MMKDLNNYREISNKELQEIKGGFGVGVGIALFMAGYTIGKDLRKKFGKSC* | *50* | *CP071917.1:189681-189833* | *34982234* |
| *Streptococcus pneumoniae* | *J4Q30_01750* | *CP071917.1* | *MMELVLKTIIGPIVVGVVLRIVDKWLNKDK* | *30* | *CP071917.1:297888-297980* | *34982234* |
| *Streptococcus pneumoniae* | *J4Q30_03570* | *CP071917.1* | *MSYKTSNAEGHVDFINTYDLEPMAQQVIPKAAFGYIASGAEDTFTSFQ* | *48* | *CP071917.1:678557-678703* | *34982234* |
| *Streptococcus pneumoniae* | *J4Q30_04595* | *CP071917.1* | *MKLLLDMKNENRLIIIATHNPDIWNQADEVINLNQL* | *36* | *CP071917.1:874732-874842* | *34982234* |
| *Streptococcus pneumoniae* | *J4Q30_05055* | *CP071917.1* | *MTCDFKFETLQLHAGQVVAPATKSRAVPIYQTTFFVFDDT* | *40* | *CP071917.1:963898-964020* | *34982234* |
| *Streptococcus pneumoniae* | *J4Q30_03110* | *CP071917.1* | *MRKEFHNVLSSGQLLTDKRPARDYNRK* | *27* | *CP071917.1:576581-576664* | *34982234* |
| *Streptococcus pneumoniae* | *J4Q30_05355* | *CP071917.1* | *MPLKTITDRVGHSDSEVTTSIYTHVTKNMKDEAINVLDKVMKKIF* | *45* | *CP071917.1:1032885-1033022* | *34982234* |
| *Streptococcus pneumoniae* | *J4Q30_09540* | *CP071917.1* | *MLVFQMRNVDKTSTVLKQTKNSDYADK* | *27* | *CP071917.1:1829399-1829482* | *34982234* |
| *Streptococcus pneumoniae* | *J4Q32_06775* | *CP071916.1* | *MAYSTDFKQRALDYIKEGHSHVEAAKFFGVGVRTLFTWEKKDVNKDT* | *47* | *CP071916.1:1329852-1329995* | *34982234* |
| *Streptococcus pneumoniae* | *J4Q32_07650* | *CP071916.1* | *MELQELVERSWAIRQAYHELEVKHHDFKWTVEEDLLALSNDIGNFQ* | *46* | *CP071916.1:1495419-1495559* | *34982234* |
| *Streptococcus pneumoniae* | *J4Q32_09320* | *CP071916.1* | *MILKYPENICMLCMPMVMHKNPSDKSIYHWDFYALLGF* | *38* | *CP071916.1:1803416-1803532* | *34982234* |
| *Streptococcus pneumoniae* | *J4Q32_10935* | *CP071916.1* | *MKQRKELYLFLGRTALYFLIFLGLLYFFSYLGQGQGSFIYNEF* | *43* | *CP071916.1:2091756-2091887* | *34982234* |
| *Streptococcus pneumoniae* | *J4Q32_11235* | *CP071916.1* | *MKNTVKLEQFVALKEKDLQKIKGGEMRLSKFFRDFILQRKK* | *41* | *CP071916.1:2155841-2155966* | *34982234* |
| *Streptococcus pneumoniae* | *J4Q32_00845* | *CP071916.1* | *MMKDLNNYREISNKELQEIKGGFGVGVGIALFMAGYTIGKDLRKKFGKSC* | *50* | *CP071916.1:152970-153122* | *34982234* |
| *Streptococcus pneumoniae* | *J4Q32_01215* | *CP071916.1* | *MVKRTCGYLGNPQARPMVNGRHKEIAARVKHMNGSTIKIAGHQVTN* | *46* | *CP071916.1:221708-221848* | *34982234* |
| *Streptococcus pneumoniae* | *J4Q32_01485* | *CP071916.1* | *MMELVLKTIIGPIVVGVVLRIVDKWLNKDK* | *30* | *CP071916.1:261177-261269* | *34982234* |
| *Streptococcus pneumoniae* | *J4Q32_02425* | *CP071916.1* | *MPDIVFEIEFFHSDSLIFFYTSDDKSNSQKSQEDFSKNK* | *39* | *CP071916.1:460326-460445* | *34982234* |
| *Streptococcus pneumoniae* | *J4Q32_02955* | *CP071916.1* | *MRSARACQFLLEQGYNVINVQGGMLAFEEL* | *30* | *CP071916.1:563146-563238* | *34982234* |
| *Streptococcus pneumoniae* | *J4Q32_03305* | *CP071916.1* | *MSYKTSNAEGHVDFINTYDLEPMAQQVIPKAAFGYIASGAEDTFTSFQ* | *48* | *CP071916.1:641846-641992* | *34982234* |
| *Streptococcus pneumoniae* | *J4Q32_03830* | *CP071916.1* | *MANDNKSHYLIYRVLGISFEEGENIDLYQNKGRFLYKYAGSFLE* | *44* | *CP071916.1:742646-742780* | *34982234* |
| *Streptococcus pneumoniae* | *J4Q32_04330* | *CP071916.1* | *MKLLLDMKNENRLIIIATHNPDIWNQADEVINLNQL* | *36* | *CP071916.1:838021-838131* | *34982234* |
| *Streptococcus pneumoniae* | *J4Q32_05090* | *CP071916.1* | *MPLKTITDRVGHSDSEVTTSIYTHVTKNMKDEAINVLDKVMKKIF* | *45* | *CP071916.1:996174-996311* | *34982234* |
| *Streptococcus pneumoniae* | *CAB39520* | *AJ240790.1* | *MKNTVKLEQFVALKEKDLQNIKGGEMRISRIILDFLFLRKK* | *41* | *AJ240790.1:50-175* | *10322016* |
| *Streptococcus pneumoniae* | *SPCG_0041* | *CP001033.1* | *MRNIGQAGKILADSGYQGLMKIYPQAQTST* | *30* | *CP001033.1:40877-40969* | *19361343* |
| *Streptococcus pneumoniae* | *SPCG_0170* | *CP001033.1* | *MLCMPMVMHKNPSDKSIYHWDFYALLGF* | *28* | *CP001033.1:171618-171704* | *19361343* |
| *Streptococcus pneumoniae* | *SPCG_0563* | *CP001033.1* | *MEFMRKEFHNVLSSGQLLADKRPARDYNRK* | *30* | *CP001033.1:559492-559584* | *19361343* |
| *Streptococcus pneumoniae* | *SPCG_1085* | *CP001033.1* | *MTCDFKFETLQLHAGQVVAPATKSRAVPIYQTTFFVFDDT* | *40* | *CP001033.1:1057162-1057284* | *19361343* |
| *Streptococcus pneumoniae* | *SPCG_1902* | *CP001033.1* | *MNYEASKQLTDTRFKRLVGVQRTTFEEMLAVLKTAYQKSRTSW* | *43* | *CP001033.1:1886655-1886786* | *19361343* |
| *Streptococcus pneumoniae* | *SPCG_1958* | *CP001033.1* | *MKRTYQPSKLRRARKHGFRNRMSTKNGRRVLAARRRKGRKVLAA* | *44* | *CP001033.1:1940452-1940586* | *19361343* |
| *Streptococcus pneumoniae* | *SPCG_2203* | *CP001033.1* | *MKNTVKLEQFVALKEKDLQKIKGGEMRLSKFFRDFILQRKK* | *41* | *CP001033.1:2206116-2206241* | *19361343* |
| *Streptococcus pneumoniae* | *ABU68098* | *EF488100.1* | *MIDATEVQINRPKKELANYSGKKKCHAMKAQAIVTSQGRLFLWISL* | *46* | *EF488100.1:8669-8809* | *17704229* |
| *Streptococcus pneumoniae* | *ABU68056* | *EF488097.1* | *MNTKMMSQFSVMDNEMLACVEGGDIDWGRKISCAAGVAYGAIDGCATTV* | *49* | *EF488097.1:3323-3472* | *17704229* |
| *Streptococcus pneumoniae* | *ABU68008* | *EF488095.1* | *MDKKQNLTSFQELTTTELNQITGGGLWEDLLYNINRYAHYIT* | *42* | *EF488095.1:2559-2687* | *17704229* |
| *Streptococcus pneumoniae* | *ABU96032* | *EF592165.1* | *MLVFQMRNVDKTSTVLKQTKNSDYADK* | *27* | *EF592165.1:2578-2661* | *17709465* |
| *Streptococcus pneumoniae* | *ABU96028* | *EF592165.1* | *MNFGQNLYNW* | *10* | *EF592165.1:1-32* | *17709465* |
| *Streptococcus pneumoniae* | *CAQ06446* | *AM889142.2* | *MLCMPMVMHKNPSDKSIYHWDFYALLGF* | *28* | *AM889142.2:1516-1602* | *18285489* |
| *Streptococcus pneumoniae* | *SPG_0128* | *CP001015.1* | *MMKDLNNYREISNKELQEIKGGFGVGVGIALFMAGYTIGKDLRKKFGKSC* | *50* | *CP001015.1:132460-132612* | *11442348* |
| *Streptococcus pneumoniae* | *SPG_0027* | *CP001015.1* | *MPAFPNVVYGAKNQKFGAAGSLYNILTDERLNHLWRLK* | *38* | *CP001015.1:23710-23826* | *11442348* |
| *Streptococcus pneumoniae* | *SPG_0359* | *CP001015.1* | *MFYGYEKTFKSLNQLEQAIIDYIDYYNNKRIKVKLKGLSPVQYRTKSFG* | *49* | *CP001015.1:350701-350850* | *11442348* |
| *Streptococcus pneumoniae* | *SPG_0022* | *CP001015.1* | *MSFIAQDFDKLNIITVLESRTQAIIRNPMNTVLSSDTESSFNKIVRN* | *47* | *CP001015.1:20757-20900* | *11442348* |
| *Streptococcus pneumoniae* | *SPG_1932* | *CP001015.1* | *MKVENISYRVDHRILFDNISFDTSSSGVTLITGKNGTGKSTLL* | *43* | *CP001015.1:1835054-1835185* | *11442348* |
| *Streptococcus pneumoniae* | *SPG_0906* | *CP001015.1* | *MSKHYRKELQAANIKVKDQAFQNIFTQYIGGGDSSSSSSTSNE* | *43* | *CP001015.1:874205-874336* | *11442348* |
| *Streptococcus pneumoniae* | *SPG_0639* | *CP001015.1* | *MKSKKSGELRIAVFGDKKPFGYVDNDGSYQGYATILN* | *37* | *CP001015.1:630080-630193* | *11442348* |
| *Streptococcus pneumoniae* | *SPG_0678* | *CP001015.1* | *MAENSXQSMEKVHADAERDNWMSAQETLEYGFIDEIMANNSLN* | *43* | *CP001015.1:662013-662144* | *11442348* |
| *Streptococcus pneumoniae* | *SPG_0866* | *CP001015.1* | *MDFEYFYNREAERFNFLKVPEILVDREEFRGLSAEAIILYSILLK* | *45* | *CP001015.1:840514-840651* | *11442348* |
| *Streptococcus pneumoniae* | *SPG_0489* | *CP001015.1* | *MDTKMMSQFAVMDNEMLACVEGGDIDWGRKISCAAGVAYGAIDGCATTV* | *49* | *CP001015.1:479741-479890* | *11442348* |
| *Streptococcus pneumoniae* | *SPG_1154* | *CP001015.1* | *MIYEFCAENVTLLEKAMQAGACRIELCDNXAVGGTTPSYGVXXGSG* | *46* | *CP001015.1:1129112-1129252* | *11442348* |
| *Streptococcus pneumoniae* | *SPG_0908* | *CP001015.1* | *MKLTYDDKVQIYELRKQGYSLEKLSNKFGINNSNIRYMIKLIDR* | *44* | *CP001015.1:874794-874928* | *11442348* |
| *Streptococcus pneumoniae* | *SPG_0478* | *CP001015.1* | *MDKKQNLTSFQELTTTELNQITGGGLWEDLLYNINRYAHYIT* | *42* | *CP001015.1:472367-472495* | *11442348* |
| *Streptococcus pneumoniae* | *SPG_2077* | *CP001015.1* | *MIDATEVQINRPKKELANDSGKKKCHAMKAQAIVTSQGRLFLWISL* | *46* | *CP001015.1:1963199-1963339* | *11442348* |
| *Streptococcus pneumoniae* | *SPG_1260* | *CP001015.1* | *MEATNNLIKLIKRNAFGFRNFENFKKRIFIALNIKKERTKCVLSRS* | *46* | *CP001015.1:1223457-1223597* | *11442348* |
| *Streptococcus pneumoniae* | *SPG_0640* | *CP001015.1* | *MKLFKPLLTVLALAFALIFITACSSGGNAGSSSGKTTAKARTIDEIKKKR* | *50* | *CP001015.1:630171-630323* | *11442348* |
| *Streptococcus pneumoniae* | *ACJ35248* | *FJ208941.1* | *MLVFQMRNVDKTSTVLKQTKNSDYTDK* | *27* | *FJ208941.1:146-229* | *19104015* |
| *Streptococcus pneumoniae* | *ACJ35247* | *FJ208941.1* | *MNKNIKY* | *7* | *FJ208941.1:1-21* | *19104015* |
| *Streptococcus pneumoniae* | *SPV_0132* | *CP027540.1* | *MMKNLNNYREISNKELQEIKGGFGVGVGIALFMAGYTIGKDLRKKFGKSC* | *50* | *CP027540.1:137824-137976* | *30107613* |
| *Streptococcus pneumoniae* | *SPV_2132* | *CP027540.1* | *MMELVLKTIIGPIVVGVVLRIVDKWLNKDK* | *30* | *CP027540.1:232359-232451* | *30107613* |
| *Streptococcus pneumoniae* | *SPV_2282* | *CP027540.1* | *MKKISKFLPILFLVMDIIIIVGG* | *23* | *CP027540.1:1015296-1015367* | *30107613* |
| *Streptococcus pneumoniae* | *SPV_1317* | *CP027540.1* | *MKSIKEEIQTIKTLLKDSRTAKYHKRLQIVLFRLMGKSYKEIIELL* | *46* | *CP027540.1:1334428-1334568* | *30107613* |
| *Streptococcus pneumoniae* | *SPV_2448* | *CP027540.1* | *MSLIIELALTIIADVIAGIILYFVCRWLDSKE* | *32* | *CP027540.1:1973363-1973461* | *30107613* |
| *Streptococcus pneumoniae* | *SPV_2450* | *CP027540.1* | *MSLIPELALTIIADVIAGIILYFVCKWLDGKK* | *32* | *CP027540.1:1973706-1973804* | *30107613* |
| *Streptococcus pneumoniae* | *SPV_2006* | *CP027540.1* | *MKQRKELYLFLGRTALYFLIFLGLLYFFSYLGQGQGSFIYNEF* | *43* | *CP027540.1:1981996-1982127* | *30107613* |
| *Streptococcus pneumoniae* | *SPV_2065* | *CP027540.1* | *MKNTVKLEQFVALKEKDLQKIKGGEMRLSKFFRDFILQRKK* | *41* | *CP027540.1:2043294-2043419* | *30107613* |
| *Streptomyces griseus* | *BAA33539* | *AB006206.3* | *MALLDLQAMDTPAEDSFGELRTGSQVSLLVCEYSSLSVVLCTP* | *43* | *AB006206.3:7096-7227* | *8458843* |
| *Pseudomonas fluorescens* | *RCC30_20865* | *CP133209.1* | *MSVLDGVSLLLAVGLFIYLLVALLRADRN* | *29* | *CP133209.1:4644338-4644427* | *38033591* |
| *Pseudomonas fluorescens* | *RCC30_26340* | *CP133209.1* | *MKVRASVKKLCRNCKIIRREGVVRVICSAEPRHKQRQG* | *38* | *CP133209.1:5808920-5809036* | *38033591* |
| *Pseudomonas fluorescens* | *RCC30_29060* | *CP133209.1* | *MSVRHWQAVLLTLVVLCGLGGCSGNYKFNDNDYRPLGDPQAVNRGK* | *46* | *CP133209.1:6380227-6380367* | *38033591* |
| *Pseudomonas fluorescens* | *RCC30_06080* | *CP133209.1* | *MFFDNVVFAGVLTVGLMVLFFAGFGFFIWKDANKRKK* | *37* | *CP133209.1:1378776-1378889* | *38033591* |
| *Pseudomonas fluorescens* | *RCC30_07055* | *CP133209.1* | *MKEKIQNWLHDLGVALGLIEPPLQPVPIRTDDEQRRRQQRRR* | *42* | *CP133209.1:1576954-1577082* | *38033591* |
| *Pseudomonas fluorescens* | *RCC30_07335* | *CP133209.1* | *MKLVELYNKIISKEPYNNMNVFFESYESFEEIPIVSRYSRLDF* | *43* | *CP133209.1:1646874-1647005* | *38033591* |
| *Pseudomonas fluorescens* | *RCC30_26925* | *CP133209.1* | *MSWTKPAYTDLRIGFEVTMYFASR* | *24* | *CP133209.1:5924952-5925026* | *38033591* |
| *Pseudomonas fluorescens* | *TK06_12565* | *CP015225.1* | *MNVLDGVSLLLAGALFIYLLVALLRADRN* | *29* | *CP015225.1:2944320-2944409* | *29769716* |
| *Pseudomonas fluorescens* | *TK06_14335* | *CP015225.1* | *MNLAAAPYALFISRTIDAPRQKIFRPRSEPALLVQGWGPQGMPD* | *44* | *CP015225.1:3347602-3347736* | *29769716* |
| *Pseudomonas fluorescens* | *TK06_21585* | *CP015225.1* | *MPVFPWRAVLLTLFVSFVLGGCSGNYKFNDSDYRPLGDPQAVNRGK* | *46* | *CP015225.1:5022027-5022167* | *29769716* |
| *Pseudomonas fluorescens* | *AO353_09405* | *CP012830.1* | *MSVRHWQAVLLTLVVLCGLGGCSGNYKFNDNTYRPLGDPQAVNRGK* | *46* | *CP012830.1:2065748-2065888* | *29769716* |
| *Pseudomonas fluorescens* | *AO353_17165* | *CP012830.1* | *MFFDNVVIAGVLTVGLMVLFFAGFGFFIWKDAHKRKKP* | *38* | *CP012830.1:3796504-3796620* | *29769716* |
| *Pseudomonas fluorescens* | *AO353_28935* | *CP012830.1* | *MTGFYLFGGLIAAGLLVYLVAALLFPEDFL* | *30* | *CP012830.1:6290085-6290177* | *29769716* |
| *Pseudomonas fluorescens* | *AO353_21180* | *CP012830.1* | *MESSITTFLALRNAQPTRYVWNAKGEDILNKIQRAREAMALRANG* | *45* | *CP012830.1:4633080-4633217* | *29769716* |
| *Halobacterium salinarum* | *VNG_0076H* | *BK010829.1* | *MRIDEFSRGMLNPCVSIVHLEVYLSELWMGVVLMPADQQ* | *39* | *BK010829.1:69300-69419* | *18313895* |
| *Halobacterium salinarum* | *VNG_0160a* | *BK010829.1* | *MGRDAPTDPNAQAAYECHICGETTTSDTHPGACPDCGTTMRNRATPLE* | *48* | *BK010829.1:135354-135500* | *18313895* |
| *Halobacterium salinarum* | *VNG_0994b* | *BK010829.1* | *MACLQHCRLQERFERRPTYRLEKCEPRCCNSRLARAPVPQLQKPC* | *45* | *BK010829.1:760234-760371* | *18313895* |
| *Halobacterium salinarum* | *VNG_1170a* | *BK010829.1* | *MVLNRLRALVDTQPGLVRECRDCGTTLGEDSDDATVCPTCGSSEIATYDL* | *50* | *BK010829.1:882933-883085* | *18313895* |
| *Halobacterium salinarum* | *VNG_1239H* | *BK010829.1* | *MTAVGACVGVSAVVVVSGMGGVRRRSGFAAYPIGGDKTI* | *39* | *BK010829.1:932347-932466* | *18313895* |
| *Halobacterium salinarum* | *VNG_1246a* | *BK010829.1* | *MVEETTRDEATWYRCEECGLVFDIDADAQQHEANCTAEDPPYLR* | *44* | *BK010829.1:937777-937911* | *18313895* |
| *Halobacterium salinarum* | *VNG_1404a* | *BK010829.1* | *MSATTRLRCHQCGRTASRDDWSSATHPSLGEMTQCPGCGSTDIQGNH* | *47* | *BK010829.1:1043476-1043619* | *18313895* |
| *Halobacterium salinarum* | *VNG_1486a* | *BK010829.1* | *MYVTPALASPARAAPFARRGWPNIKIPTGKWRH* | *33* | *BK010829.1:1105831-1105932* | *18313895* |
| *Halobacterium salinarum* | *VNG_1642H* | *BK010829.1* | *MPTEAPGCPRCSAALFKRHCKYVCPQHGVVYDCADTFWLS* | *40* | *BK010829.1:1220972-1221094* | *18313895* |
| *Halobacterium salinarum* | *VNG_1832H* | *BK010829.1* | *MLGNEKRRLLFPAAIRNDTRGYRSSHDDSRLQCVRFTFEKAGHGEH* | *46* | *BK010829.1:1352444-1352584* | *18313895* |
| *Halobacterium salinarum* | *VNG_2047G* | *BK010829.1* | *MARGDYYSDDGTTDKEMCPRCGDTFLAAHDDRQVCGRCGYTEWE* | *44* | *BK010829.1:1508435-1508569* | *18313895* |
| *Halobacterium salinarum* | *VNG_2076G* | *BK010829.1* | *MSETIEDRLLNKQVCMRCNARNPTDAESCRKCGYKNLRTKASERRSA* | *47* | *BK010829.1:1526849-1526992* | *18313895* |
| *Halobacterium salinarum* | *VNG_2469G* | *BK010829.1* | *MGKKSKASKKRLAKLERQNSRVPAWVMMKTNRDVQRNPKRRNWRRNDTDE* | *50* | *BK010829.1:1846565-1846717* | *18313895* |
| *Halobacterium salinarum* | *HBSAL_00180* | *CP038631.1* | *MPKLDCPDCGRSIAMHELETRTVAQTAGFETSYRCPFCRTDFQEVTQLM* | *49* | *CP038631.1:33679-33828* | *31296677* |
| *Halobacterium salinarum* | *HBSAL_01645* | *CP038631.1* | *MGRDAPTDPNAQAAYECHICGETTTSDTHPGACPDCGTTMRNRATPLE* | *48* | *CP038631.1:316565-316711* | *31296677* |
| *Halobacterium salinarum* | *HBSAL_01905* | *CP038631.1* | *MSYKCSRCKRDVELDEYGGVRCPYCGHRVLLKERSRDVKEVSVQ* | *44* | *CP038631.1:368246-368380* | *31296677* |
| *Halobacterium salinarum* | *HBSAL_06690* | *CP038631.1* | *MVLNRLRALVDTQPGLVRECRDCGTTLGEDSDDATVCPTCGSSEIATYDL* | *50* | *CP038631.1:1245932-1246084* | *31296677* |
| *Halobacterium salinarum* | *HBSAL_07975* | *CP038631.1* | *MYVTPALASPARAAPFARRGWPNIKIPTGKWRH* | *33* | *CP038631.1:1472703-1472804* | *31296677* |
| *Halobacterium salinarum* | *HBSAL_10245* | *CP038631.1* | *MARGDYYSDDGTTDKEMCPRCGDTFLAAHDDRQVCGRCGYTEWE* | *44* | *CP038631.1:1884827-1884961* | *31296677* |
| *Halobacterium salinarum* | *HBSAL_10345* | *CP038631.1* | *MSETIEDRLLNKQVCMRCNARNPTDAESCRKCGYKNLRTKASERRSA* | *47* | *CP038631.1:1903241-1903384* | *31296677* |
| *Halobacterium salinarum* | *OE_1372R* | *AM774415.1* | *MSYKCSRCKRDVELDEYGGVRCPYCGHRVLLKERSRDVKEVSVQ* | *44* | *AM774415.1:187199-187333* | *18313895* |
| *Halobacterium salinarum* | *OE_2446A1F* | *AM774415.1* | *MACLQHCRLQERFERRPTYRLEKCEPRCCNSRLARAPVPQLQKPC* | *45* | *AM774415.1:752754-752891* | *18313895* |
| *Halobacterium salinarum* | *OE_3130F* | *AM774415.1* | *MYVTPALASPARAAPFARRGWPNIKIPTGKWRH* | *33* | *AM774415.1:1098950-1099051* | *18313895* |
| *Haloferax volcanii* | *HVO_1670A* | *CP001956.1* | *MERASYVCRECQTTVSAASYRSTCPECSGELRPTSMTSRRRAGD* | *44* | *CP001956.1:1531014-1531148* | *20333302* |
| *Haloferax volcanii* | *HVO_2523* | *CP001956.1* | *MDDDAGRNACAIESPKGRPCPFCGTSMDHRHCKYVCPEHGVVYDCSDTFW* | *50* | *CP001956.1:2389183-2389335* | *20333302* |
| *Haloferax volcanii* | *HVO_0115* | *CP001956.1* | *MGKKSKSKKKRLAKLERQNSRVPAWVMLKTDMEVTRNPKRRNWRRSNTDE* | *50* | *CP001956.1:110903-111055* | *20333302* |
| *Haloferax volcanii* | *HVO_0325* | *CP001956.1* | *MSQCNHCDAFVSNNFVRVFGDEDGNVYACPSCSANAGISQVSSERRASSL* | *50* | *CP001956.1:295413-295565* | *20333302* |
| *Haloferax volcanii* | *HVO_0439* | *CP001956.1* | *MASDAATKRTLEKQICMRCNARNPQKAKQCRKCGYKHLRPKSKERRAA* | *48* | *CP001956.1:391882-392028* | *20333302* |
| *Haloferax volcanii* | *HVO_0439* | *CP001956.1* | *MASDAATKRTLEKQICMRCNARNPQKAKQCRKCGYKHLRPKSKERRAA* | *48* | *CP001956.1:391882-392028* | *20333302* |
| *Haloferax volcanii* | *HVO_0241* | *CP001956.1* | *MPAVQCRECGRDVAVHEIETTTKTTPDGFDTRYRCPYCKSEMRDVKTRIV* | *50* | *CP001956.1:217905-218057* | *20333302* |
| *Haloferax volcanii* | *HVO_0653* | *CP001956.1* | *MSYKCSRCKRDVELDEFGGVRCPYCGHRVLLKERSPNVKEVAVE* | *44* | *CP001956.1:584183-584317* | *20333302* |
| *Human betaherpesvirus* | *UBQ34274* | *MZ327301.1* | *MSLDAAGHQPEARRLLDSALVRRVLACMIIVIMIAISIWILTYVLFL* | *47* | *MZ327301.1:179017-179160* | *34201364* |
| *Human betaherpesvirus* | *UQK63149* | *ON023031.1* | *MMIFITCSWTTEKDTEKSEVQSYASSVETLDSLNEAIIPKTEMNV* | *45* | *ON023031.1:138996-139133* | *36951096* |
| *Human betaherpesvirus* | *HHV5gp_153* | *MZ327301.1* | *MDMRKLNTSQGRNLTTVDDRRRFSIGWETWDNGGD* | *35* | *MZ327301.1:5393-5500* | *34201364* |
| *Human betaherpesvirus* | *QUP52091* | *MW980585.1* | *MSLDAASHQPAARRLLDSALVRRVLACMIIVIMIAISIWILTYVLFF* | *47* | *MW980585.1:8121-8264* | *33767437* |
| *Mycobacterium tuberculosis* | *CAA63256* | *X92504.1* | *MTKGKRTFQPNNRRRARVHGFRLRMRTRAGRSIVSSRRRKGRRTLSA* | *47* | *X92504.1:839-982* | *8733228* |
| *Mycobacterium tuberculosis* | *AAB17596* | *U15140.1* | *MKVNPSVKPICDKCRLIRRHGRVMVICSDPRHKQRQG* | *37* | *U15140.1:599-712* | *8621083* |
| *Mycobacterium tuberculosis* | *Rv3924c* | *NC_000962.3* | *MTKGKRTFQPNNRRRARVHGFRLRMRTRAGRSIVSSRRRKGRRTLSA* | *47* | *NC_000962.3:4410786-4410929* | *12368430* |
| *Mycobacterium tuberculosis* | *MRGA423_01010* | *CP003234.1* | *MTATASGIAATAPNCGEASINDVPIAESERRYLGARSASEYGQEIPLW* | *48* | *CP003234.1:183527-183673* | *22843573* |
| *Mycobacterium tuberculosis* | *MRGA423_02770* | *CP003234.1* | *MAKTIAYDEEARRGLERGLNALADAVKVTLGPKGRNVVLEKK* | *42* | *CP003234.1:528972-529100* | *22843573* |
| *Mycobacterium tuberculosis* | *MRGA423_03960* | *CP003234.1* | *MRPKITLACEVCKHRNYITKKNRRNDPDRLELKKFCPNCGKHQAHRETR* | *49* | *CP003234.1:732225-732374* | *22843573* |
| *Mycobacterium tuberculosis* | *MRGA423_04150* | *CP003234.1* | *MLSSDGAAIELREGEDEDLERAAANLGINLSRNESASVEDLA* | *42* | *CP003234.1:763719-763847* | *22843573* |
| *Mycobacterium tuberculosis* | *MRGA423_04630* | *CP003234.1* | *MLGLAMEAVASTGAAAPLGSHAAQIYAKFAADHADLDFSAVIETLRGS* | *48* | *CP003234.1:835640-835786* | *22843573* |
| *Mycobacterium tuberculosis* | *MRGA423_04900* | *CP003234.1* | *MAALRDPNARANPAGAELATWSLVHGFSTLWARRCGQR* | *38* | *CP003234.1:877311-877427* | *22843573* |
| *Mycobacterium tuberculosis* | *MRGA423_06760* | *CP003234.1* | *MHAHPDDESSKGAATLARYADEGPSRAGGDVDRW* | *34* | *CP003234.1:1207413-1207517* | *22843573* |
| *Mycobacterium tuberculosis* | *MRGA423_06810* | *CP003234.1* | *MNGAAPTNGAPLSYPSICEGVHWGHLVGGHQPAY* | *34* | *CP003234.1:1216392-1216496* | *22843573* |
| *Mycobacterium tuberculosis* | *MRGA423_08695* | *CP003234.1* | *MTTLAGDDFGGGPTMPMMPGTWTHDQGVFDEHR* | *33* | *CP003234.1:1564382-1564483* | *22843573* |
| *Mycobacterium tuberculosis* | *MRGA423_09635* | *CP003234.1* | *MESRWVLHLDMDAFFASVEQLTRPTLRGRPVLVGGLGGRGVVAGREL* | *47* | *CP003234.1:1739061-1739204* | *22843573* |
| *Mycobacterium tuberculosis* | *MRGA423_11205* | *CP003234.1* | *MRPEDMQLTRDGVAACPEMALILEEDDAD* | *29* | *CP003234.1:2024359-2024448* | *22843573* |
| *Mycobacterium tuberculosis* | *MRGA423_14345* | *CP003234.1* | *MTLDRHGHLLSDDLAGVAGLLVQAIKSAAASLRYSDPDSVAVENISAAS* | *49* | *CP003234.1:2581547-2581696* | *22843573* |
| *Mycobacterium tuberculosis* | *MRGA423_18085* | *CP003234.1* | *MKLVGLGEGPDDLAPFEPAAFVDALLG* | *27* | *CP003234.1:3227022-3227105* | *22843573* |
| *Mycobacterium tuberculosis* | *MRGA423_18775* | *CP003234.1* | *MVTLSVVPEGLAAASAAVEALTARLAAAH* | *29* | *CP003234.1:3372573-3372662* | *22843573* |
| *Mycobacterium tuberculosis* | *MRGA423_19875* | *CP003234.1* | *MATARWVDWFNHRRLYQYCGDVPPVELEAAYYAQRQRPAAG* | *41* | *CP003234.1:3546811-3546936* | *22843573* |
| *Mycobacterium tuberculosis* | *MRGA423_21810* | *CP003234.1* | *MKVNPSVKPICDKCRLIRRHGRVMVICSDPRHKQRQG* | *37* | *CP003234.1:3874924-3875037* | *22843573* |
| *Mycobacterium tuberculosis* | *MRGA423_23965* | *CP003234.1* | *MTVRDVLLVSAGTIPRTSSGKIGRRACRAAYLDGSLRSGVGSPTVFATSD* | *50* | *CP003234.1:4256101-4256253* | *22843573* |
| *Mycobacterium tuberculosis* | *MRGA423_24055* | *CP003234.1* | *MWSEMDAIEVDEQVVTRAADLAHAWLRRGALRIGRATR* | *38* | *CP003234.1:4273145-4273261* | *22843573* |
| *Mycobacterium tuberculosis* | *MRGA423_24280* | *CP003234.1* | *MTTVNPPTEAISGRVEHLRGSTLGFRNLTN* | *30* | *CP003234.1:4315152-4315244* | *22843573* |
| *Mycobacterium tuberculosis* | *MRGA423_24765* | *CP003234.1* | *MTKGKRTFQPNNRRRARVHGFRLRMRTRAGRSIVSSRRRKGRRTLSA* | *47* | *CP003234.1:4405841-4405984* | *22843573* |
| *Mycobacterium tuberculosis* | *MRGA327_00940* | *CP003233.1* | *MLTKPTRPDLSSFIYPPYTERAIKVARRLF* | *30* | *CP003233.1:174772-174864* | *22843573* |
| *Mycobacterium tuberculosis* | *MRGA327_00990* | *CP003233.1* | *MTATASGIAATAPNCGEASINDVPIAESERRYLGARSASEYGQEIPLW* | *48* | *CP003233.1:183524-183670* | *22843573* |
| *Mycobacterium tuberculosis* | *MRGA327_03380* | *CP003233.1* | *MKAADVFAAFGENIELLKRLVRAAIDRVADERTCTHCQHHAGVPLPFELP* | *50* | *CP003233.1:627510-627662* | *22843573* |
| *Mycobacterium tuberculosis* | *MRGA327_03980* | *CP003233.1* | *MRPKITLACEVCKHRNYITKKNRRNDPDRLELKKFCPNCGKHQAHRETR* | *49* | *CP003233.1:732188-732337* | *22843573* |
| *Mycobacterium tuberculosis* | *MRGA327_04175* | *CP003233.1* | *MHALGHDAGLAARLALPAAPFNMSGNPAICLPAGDTS* | *37* | *CP003233.1:767901-768014* | *22843573* |
| *Mycobacterium tuberculosis* | *MRGA327_04630* | *CP003233.1* | *MLGLAMEAVASTGAAAPLGSHAAQIYAKFAADHADLDFSAVIETLRGS* | *48* | *CP003233.1:835557-835703* | *22843573* |
| *Mycobacterium tuberculosis* | *MRGA327_04885* | *CP003233.1* | *MAALRDPNARANPAGAELATWSLVHGFSTLWLDDAVNADVKQTSCG* | *46* | *CP003233.1:877213-877353* | *22843573* |
| *Mycobacterium tuberculosis* | *MRGA327_05970* | *CP003233.1* | *MTHDKYGLGRVEEVSGVGESAMSLIDFGSSGRVKLMHNHAPVTKL* | *45* | *CP003233.1:1061091-1061228* | *22843573* |
| *Mycobacterium tuberculosis* | *MRGA327_08295* | *CP003233.1* | *MSDDIDAAFAAADVQVLNQDVSAAFERLIAIGRVGHLEKSAPGCAPG* | *47* | *CP003233.1:1488761-1488904* | *22843573* |
| *Mycobacterium tuberculosis* | *MRGA327_09060* | *CP003233.1* | *MFDQLGAIGIDLTDVFAVLEEEGVRKFEASWNELLQETRAHLDTAAQ* | *47* | *CP003233.1:1627938-1628081* | *22843573* |
| *Mycobacterium tuberculosis* | *MRGA327_14480* | *CP003233.1* | *MILDFSWLPPEINSARIYAGAGSGPLFMAAAAWEGLAADLRALGVLV* | *47* | *CP003233.1:2627493-2627636* | *22843573* |
| *Mycobacterium tuberculosis* | *MRGA327_14795* | *CP003233.1* | *MRATRVPPIAGGVSGVDDAGVERVKVTST* | *29* | *CP003233.1:2687484-2687573* | *22843573* |
| *Mycobacterium tuberculosis* | *MRGA327_18470* | *CP003233.1* | *MATTSPVVIKVSPMAHFAVGFLTLGLLVPVLTWAG* | *35* | *CP003233.1:3357285-3357392* | *22843573* |
| *Mycobacterium tuberculosis* | *MRGA327_19785* | *CP003233.1* | *MLNWNSDAIALYDRIGGQPQHEWTIYRLSGPRLAALAAPR* | *40* | *CP003233.1:3587834-3587956* | *22843573* |
| *Mycobacterium tuberculosis* | *MRGA327_20430* | *CP003233.1* | *MLWIIGSVFLLLMVPAGVVVGIHMWEHFR* | *29* | *CP003233.1:3698532-3698621* | *22843573* |
| *Mycobacterium tuberculosis* | *MRGA327_21350* | *CP003233.1* | *MKVNPSVKPICDKCRLIRRHGRVMVICSDPRHKQRQG* | *37* | *CP003233.1:3849581-3849694* | *22843573* |
| *Mycobacterium tuberculosis* | *MRGA327_21940* | *CP003233.1* | *MHDLDAAEQTRLPTDDELHLIRAVIDPKSLRDREIRS* | *37* | *CP003233.1:3959770-3959883* | *22843573* |
| *Mycobacterium tuberculosis* | *MRGA327_23480* | *CP003233.1* | *MWSEMDAIEVDEQVVTRAADLAHAWLRRGALRIGRATR* | *38* | *CP003233.1:4246685-4246801* | *22843573* |
| *Mycobacterium tuberculosis* | *MRGA327_23675* | *CP003233.1* | *MTTVNPPTEAISGRVEHLRGSTLGFRNLTN* | *30* | *CP003233.1:4288684-4288776* | *22843573* |
| *Mycobacterium tuberculosis* | *ERDMAN_0040* | *AP012340.1* | *MPEERSGFRTSYLADHRQSDLDRALHVFAGLAEDLTPQGAAL* | *42* | *AP012340.1:36462-36590* | *22535945* |
| *Mycobacterium tuberculosis* | *ERDMAN_0311* | *AP012340.1* | *MLIGNGGNGGNGGKAGGTPGAGGTSGLLIGENGLNGLP* | *38* | *AP012340.1:335827-335943* | *22535945* |
| *Mycobacterium tuberculosis* | *ERDMAN_0702* | *AP012340.1* | *MRPKITLACEVCKHRNYITKKNRRNDPDRLELKKFCPNCGKHQAHRETR* | *49* | *AP012340.1:732695-732844* | *22535945* |
| *Mycobacterium tuberculosis* | *ERDMAN_0820* | *AP012340.1* | *MLGLAMEAVASTGAAAPLGSHAAQIYAKFAADHADLDFSAVIETLRGS* | *48* | *AP012340.1:836665-836811* | *22535945* |
| *Mycobacterium tuberculosis* | *ERDMAN_0826* | *AP012340.1* | *MLIGNGGNGGNGGNSGNAGKSGGAPGPSGAGGAGGLLLGENGLNGLP* | *47* | *AP012340.1:840621-840764* | *22535945* |
| *Mycobacterium tuberculosis* | *ERDMAN_1954* | *AP012340.1* | *MAPRQRTNPPVDAQGRAVRRATAVFTTVTDVADHGRLIW* | *39* | *AP012340.1:1990593-1990712* | *22535945* |
| *Mycobacterium tuberculosis* | *ERDMAN_1982* | *AP012340.1* | *MNQAFRNIVNMLHGVRDGLVRDANNYEQQEQASQQILSS* | *39* | *AP012340.1:2021728-2021847* | *22535945* |
| *Mycobacterium tuberculosis* | *ERDMAN_2345* | *AP012340.1* | *MVFLAMAVVAVTAYFRMGWWSIIGYAAAAIIGVIGFALAFRDLS* | *44* | *AP012340.1:2383044-2383178* | *22535945* |
| *Mycobacterium tuberculosis* | *ERDMAN_2523* | *AP012340.1* | *MALGWAMRRKPFSRFNEYVGSASNTCWFARALELRTLLIR* | *40* | *AP012340.1:2563954-2564076* | *22535945* |
| *Mycobacterium tuberculosis* | *ERDMAN_4179* | *AP012340.1* | *MRGYDAVHCASAEQLDDDEVVAAAADQRLLTAWLELGMATYDTNQRATPR* | *50* | *AP012340.1:4258990-4259142* | *22535945* |
| *Mycobacterium tuberculosis* | *UDA_1028A* | *HE608151.1* | *MTTVDNIVGLVIAVALMAFLFAALLFPEKF* | *30* | *HE608151.1:1154428-1154520* | *22404577* |
| *Mycobacterium tuberculosis* | *UDA_1089A* | *HE608151.1* | *MNGAAPTNGAPLSYPSICEGVHWGHLVGGHQPAY* | *34* | *HE608151.1:1218083-1218187* | *22404577* |
| *Mycobacterium tuberculosis* | *UDA_1549* | *HE608151.1* | *MVAAPVLPRPSIVDVRSPM* | *19* | *HE608151.1:1756573-1756632* | *22404577* |
| *Mycobacterium tuberculosis* | *UDA_3461c* | *HE608151.1* | *MKVNPSVKPICDKCRLIRRHGRVMVICSDPRHKQRQG* | *37* | *HE608151.1:3881511-3881624* | *22404577* |
| *Mycobacterium tuberculosis* | *UDA_3924c* | *HE608151.1* | *MTKGKRTFQPNNRRRARVHGFRLRMRTRAGRSIVSSRRRKGRRTLSA* | *47* | *HE608151.1:4417342-4417485* | *22404577* |
| *Mycolicibacterium smegmatis* | *D806_005760* | *CP027541.1* | *MDPKHNDDQGDRPGDTGYDPDADPEMMQQQAHPQPNQAEGADDPAETGEG* | *50* | *CP027541.1:668051-668203* | *23618714* |
| *Mycolicibacterium smegmatis* | *D806_010040* | *CP027541.1* | *MRVSKMTVYRLVHNGELPAVRVGRSFRVHAKAVHDLLETSYFDAG* | *45* | *CP027541.1:1115055-1115192* | *23618714* |
| *Mycolicibacterium smegmatis* | *D806_019790* | *CP027541.1* | *MNARYTSDTLRTARSFLFVPGNRPERFSKAVAAQPDVVVLDLE* | *43* | *CP027541.1:2120174-2120305* | *23618714* |
| *Mycolicibacterium smegmatis* | *D806_033760* | *CP027541.1* | *MEGMIPRGELGQPREVASAALFLACDDSSFVNGQLVNVDGGATAI* | *45* | *CP027541.1:3568977-3569114* | *23618714* |
| *Mycolicibacterium smegmatis* | *D806_037190* | *CP027541.1* | *MEKKSAGALQKWERPTFAEIRVSAEVTAYVAVLDGDD* | *37* | *CP027541.1:3925592-3925705* | *23618714* |
| *Mycolicibacterium smegmatis* | *D806_039950* | *CP027541.1* | *MHVRTRAGHDSTNMKDVVPTVMLFIPSVDGVSHAENEFTHDKDRVCCTNR* | *50* | *CP027541.1:4216081-4216233* | *23618714* |
| *Mycolicibacterium smegmatis* | *D806_061000* | *CP027541.1* | *MRLSGLSTEVPVGARDGLEKDSVVSVDHFVTIPVASLGRRIGLFLRD* | *47* | *CP027541.1:6401882-6402025* | *23618714* |
| *Mycolicibacterium smegmatis* | *D806_068190* | *CP027541.1* | *MAKGKRTFQPNNRRRARVHGFRLRMRTRAGRAIVANRRSKGRRALTA* | *47* | *CP027541.1:7116902-7117045* | *23618714* |
| *Mycolicibacterium smegmatis* | *LJ00_07595* | *CP009494.1* | *MKVNPSVKPICDKCRVIRRHGRVMVICSDPRHKQRQG* | *37* | *CP009494.1:1611925-1612038* | *25657281* |
| *Mycolicibacterium smegmatis* | *LJ00_08980* | *CP009494.1* | *MDGGSPHTLRRLLISLALILAVVIAIVAAVYAMAFIMLAPMMT* | *43* | *CP009494.1:1880531-1880662* | *25657281* |
| *Mycolicibacterium smegmatis* | *LJ00_09950* | *CP009494.1* | *MNARYTTDTLRTARSFLFVPGNRPERFSKAVAAQPDVVVLDLE* | *43* | *CP009494.1:2076547-2076678* | *25657281* |
| *Mycolicibacterium smegmatis* | *LJ00_18510* | *CP009494.1* | *MEKKSAGALQKWERPTFAEIRVSAEVTAYVAVLDGDD* | *37* | *CP009494.1:3792200-3792313* | *25657281* |
| *Mycolicibacterium smegmatis* | *LJ00_26480* | *CP009494.1* | *MPIARDVDCAGGNGNGPEYVSDPVYVVGPDEYELDRDGDGVACEK* | *45* | *CP009494.1:5439127-5439264* | *25657281* |
| *Mycolicibacterium smegmatis* | *LJ00_34280* | *CP009494.1* | *MAKGKRTFQPNNRRRARVHGFRLRMRTRAGRAIVANRRSKGRRALTA* | *47* | *CP009494.1:6986003-6986146* | *25657281* |
| *Mycoplasmoides pneumoniae* | *AAC43693* | *U34795.1* | *MKVRASVKPICKDCKIIKRHQIVRVICKTQKHKQRQG* | *37* | *U34795.1:17072-17185* | *8604303* |
| *Mycoplasmoides pneumoniae* | *MPNE_0714* | *CP002077.1* | *MPIVSFANFFLREAAFLLIIQPMAVAPKANTPAIADKDQVNIWLVLALV* | *49* | *CP002077.1:727607-727756* | *20543037* |
| *Mycoplasmoides pneumoniae* | *MPNE_0151* | *CP002077.1* | *MQIFNFWNWSAKRLIPVGNRVASVSYFLKNQKKKERNI* | *38* | *CP002077.1:168906-169022* | *20543037* |
| *Mycoplasmoides pneumoniae* | *MPNE_0793* | *CP002077.1* | *MREQFCNLNAVDVLVIINQGFLELTFKEKQTIFLNLCQKLQELDAPKPK* | *49* | *CP002077.1:800049-800198* | *20543037* |
| *Mycoplasmoides pneumoniae* | *MPNE_0217* | *CP002077.1* | *MKVRASVKPICKDCKIIKRHQIVRVICKTQKHKQRQG* | *37* | *CP002077.1:228841-228954* | *20543037* |
| *Mycoplasmoides pneumoniae* | *MPNE_0794* | *CP002077.1* | *MKRTYQPSKLKRAKTHGFLARMATASGRKVLKLRRKKQRAQLTVSSER* | *48* | *CP002077.1:800395-800541* | *20543037* |
| *Staphylococcus aureus* | *AAB63268* | *AF001783.1* | *MKKLLNKVIELLVDFFNSIGYRAAYINCDFLLDEAEVPKELTQLHE* | *46* | *AF001783.1:568-708* | *9197262* |
| *Staphylococcus aureus* | *AAB63265* | *AF001782.1* | *MNTLVNMFFDFIIKLAKAIGIVGGVNACSSLFDEPKVPAELTNLYDK* | *47* | *AF001782.1:567-710* | *9197262* |
| *Staphylococcus aureus* | *BAA34538* | *AB013298.1* | *MTASMRLK* | *8* | *AB013298.1:231-257* | *9197262* |
| *Staphylococcus aureus* | *AAG03056* | *AF288215.1* | *MNTLYKSFFDFITGVLKNIGNVASYSTCYFIMDEVEIPKELTQLHE* | *46* | *AF288215.1:1283-1423* | *11053400* |
| *Staphylococcus aureus* | *AAG03054* | *AF288215.1* | *MAQDIISTISDLVKWIIDTVNKFTKK* | *26* | *AF288215.1:347-427* | *11053400* |
| *Caulobacter vibrioides* | *CCNA_03992* | *NC_011916.1* | *MIYGCIGRVRTATIELRLRSGLAWR* | *25* | *NC_011916.1:3621641-3621718* | *20472802* |
| *Caulobacter vibrioides* | *CCNA_00468* | *NC_011916.1* | *MQPGDITGAYHNIDKVHALCGYMSEVTLAEGLASLVA* | *37* | *NC_011916.1:481820-481933* | *20472802* |
| *Caulobacter vibrioides* | *CCNA_00547* | *NC_011916.1* | *MNSETAPAPDRGRLKMADIARMAGVSISTVSRDLPLIFSSTRS* | *43* | *NC_011916.1:565748-565879* | *20472802* |
| *Caulobacter vibrioides* | *CCNA_00802* | *NC_011916.1* | *MWYFTWILGLGLAVAFGVLNGMWYEFSLSDEGDEGLDEA* | *39* | *NC_011916.1:867576-867695* | *20472802* |
| *Caulobacter vibrioides* | *CCNA_00808* | *NC_011916.1* | *MKRTFQPSKLVRARRHGYRARMATKNGQKVVARRRAKGRKRLTA* | *44* | *NC_011916.1:872176-872310* | *20472802* |
| *Caulobacter vibrioides* | *CCNA_00964* | *NC_011916.1* | *MWGAATDTGPLAAEALRRRLGGLGPKRPLP* | *30* | *NC_011916.1:1039648-1039740* | *20472802* |
| *Caulobacter vibrioides* | *CCNA_01910* | *NC_011916.1* | *MVFSAWPVATAARGKAFAENRPDARHQSGQHVDALRLLA* | *39* | *NC_011916.1:2055307-2055426* | *20472802* |
| *Caulobacter vibrioides* | *CCNA_02004* | *NC_011916.1* | *MIRVARESFALVSVIGFVWMMCTVANLVA* | *29* | *NC_011916.1:2152374=2152463* | *20472802* |
| *Caulobacter vibrioides* | *CCNA_02506* | *NC_011916.1* | *MQRLNASALRRLDSPGFDPDGDGLHSQIAGTGGRSWVFRYWVRGRE* | *46* | *NC_011916.1:2656074-2656214* | *20472802* |
| *Caulobacter vibrioides* | *CCNA_02777* | *NC_011916.1* | *MYLRASHQIDGTLKVYGRVENALDKDYQTILSYGTPGRGAFFGL* | *44* | *NC_011916.1:2936207-2936341* | *20472802* |
| *Caulobacter vibrioides* | *CCNA_02879* | *NC_011916.1* | *MPFGWPLHRRSAVPLPRWGRRGVRRGQISRS* | *31* | *NC_011916.1:3026790-3026885* | *20472802* |
| *Caulobacter vibrioides* | *CCNA_03078* | *NC_011916.1* | *MRLTVTIDDALMAEALKATGLKSKRAAVGITVTVELR* | *37* | *NC_011916.1:3228145-3228258* | *20472802* |
| *Caulobacter vibrioides* | *CCNA_03587* | *NC_011916.1* | *MMRKLVILAVLAASLSVAACNTVEGAGKDVSSAGKAVTDTARDVKKN* | *47* | *NC_011916.1:3742020-3742163* | *20472802* |
| *Caulobacter vibrioides* | *CCNA_03762* | *NC_011916.1* | *MTMKTNAVAGIASLFLAVLPLVVIAGSLASSL* | *32* | *NC_011916.1:3931582-3931680* | *20472802* |
| *Caulobacter vibrioides* | *CCNA_01074* | *CP001340.1* | *MRKFVVLAVAASALLVSACNTIEGVGRDVSAAGKAVSGAARDAKH* | *45* | *CP001340.1:1179617-1179754* | *20472802* |
| *Caulobacter vibrioides* | *CCNA_03430* | *CP001340.1* | *MKVRSSLKSLKGRHRDCKMVRRKGVIYIINKTDPRFKAKQG* | *41* | *CP001340.1:3593983-3594108* | *20472802* |
| *Caulobacter vibrioides* | *CC_0769* | *AE005673.1* | *MKRTFQPSKLVRARRHGYRARMATKNGQKVVARRRAKGRKRLTA* | *44* | *AE005673.1:846145-846279* | *11259647* |
| *Caulobacter vibrioides* | *CC_3321* | *AE005673.1* | *MKVRSSLKSLKGRHRDCKMVRRKGVIYIINKTDPRFKAKQG* | *41* | *AE005673.1:3567994-3568119* | *16829582* |
| *Clostridium aceticum* | *CACET_c15490* | *CP009687.1* | *METVKCKHCGRTLCELEGKIKIKCRKCGHWNYIQTKEAAQAAQSE* | *45* | *CP009687.1:1636444-1636581* | *26184942* |
| *Clostridium aceticum* | *CACET_c18940* | *CP009687.1* | *MTHGAVITREYGIPSVVGMDNATKLIKDGQSVLINGTEGYV* | *41* | *CP009687.1:1962812-1962937* | *26184942* |
| *Clostridium aceticum* | *CACET_c26500* | *CP009687.1* | *MDEIWGMEAETDERTVDVHIKRLREKFSDCSEFEIVTVRRLGYKAEIKA* | *49* | *CP009687.1:2791782-2791931* | *26184942* |
| *Clostridium aceticum* | *CACET_c26750* | *CP009687.1* | *MLINEISKITELTKKAIEYYVEKELISPSILEKGDKGTRLLR* | *42* | *CP009687.1:2816695-2816823* | *26184942* |
| *Clostridium aceticum* | *CACET_c35980* | *CP009687.1* | *MKVRPSVKPICEKCKIIKRKGKVMVICENPKHKQKQG* | *37* | *CP009687.1:3811215-3811328* | *26184942* |
| *Clostridium ljungdahlii* | *CDI37303* | *HG525909.1* | *ENNNENLALAA* | *11* | *HG525909.1:99-134* | *14681369* |
| *Clostridium ljungdahlii* | *CLJU_c40810* | *CP001666.1* | *MKVRPSVKPMCEKCKIIKRKGRVMVICENPKHKQKQG* | *37* | *CP001666.1:4434135-4434248* | *20616070* |
| *Clostridium ljungdahlii* | *CLJU_c41190* | *CP001666.1* | *MRVKVILACTDCKQRNYNTMKNKKNDPDRLEMKKYCPFCHKHTVHKETR* | *49* | *CP001666.1:4462025-4462174* | *20616070* |
| *Clostridium ljungdahlii* | *CLJU_c42970* | *CP001666.1* | *MWMTYQPKKKQRKREHGFRKRMRTLSGRNVIRRRRQKGRKRLTA* | *44* | *CP001666.1:4629434-4629568* | *20616070* |
| *Flavobacterium johnsoniae* | *CDI38533* | *HG527153.1* | *GENNYALAA* | *9* | *HG527153.1:96-125* | *14681369* |
| *Flavobacterium johnsoniae* | *CDK10671* | *HG789014.1* | *GENNYALAA* | *9* | *HG789014.1:96-125* | *14681369* |
| *Flavobacterium johnsoniae* | *Fjoh_0375* | *CP000685.1* | *MKVRASVKKRSAECIIVRRKGRLYVINKKNPRFKQRQG* | *38* | *CP000685.1:396894-397010* | *19717629* |
