# Supplementary material for: RiboMicrobe: An Integrated Translatome Atlas for Microorganism
Source: Adv Sci (Weinh). 2025 Oct 13;12(48):e09877. doi: 10.1002/advs.202509877 (PMC12752654; doi:10.1002/advs.202509877)
Supplement: Supplementary file 2 — Supplemental Table S1–S6 [file ADVS-12-e09877-s002.zip › re_Table S3.docx]

**Table S3. Summary of Reported Micropeptides and Their Overlap with Model Prediction Results.**

| Species | Chrom | Start | End | Strand | Frame | Length | Probability | Prediction | Protein_Sequence |
| --- | --- | --- | --- | --- | --- | --- | --- | --- | --- |
| Bacillus subtilis | Chromosome | 752078 | 752252 | + | 3 | 174 | 0.9999 | 1 | MPIKKKVMMCLAVTLVFGSMSFPTLTNSGGFKESTDRNTTYIDHSPYKLSDQKKALS |
| Bacillus subtilis | Chromosome | 3836057 | 3836189 | + | 3 | 132 | 0.7795 | 1 | MKKAVIVENKGCATCSIGAACLVDGPIPDFEIAGATGLFGLWG |
| Bacillus subtilis | Chromosome | 430355 | 430469 | - | 2 | 114 | 0.9599 | 1 | MSGYSNGGGYGGISSFALIVVLFILLIIVGTAFVGGF |
| Bacillus subtilis | Chromosome | 2154886 | 2155018 | - | 3 | 132 | 0.9786 | 1 | MDSREQIDWTCNECNFSWIGDNSDFSCPSCDEIDIKPKNKIL |
| Escherichia coli | Chromosome | 189 | 255 | + | 0 | 66 | 0.9963 | 1 | MKRISTTITTTITITTGNGAG |
| Escherichia coli | Chromosome | 1580925 | 1581099 | + | 0 | 174 | 1 | 1 | MTTLIYLQIPVPEPIPGDPVPVPDPIPRPQPMPDPPPDEEPIKLSHRERRSARIRAC |
| Escherichia coli | Chromosome | 2249832 | 2249883 | + | 0 | 51 | 0.7358 | 1 | MTRVQFKHHHHHHHPD |
| Escherichia coli | Chromosome | 2389950 | 2390034 | + | 0 | 84 | 0.7129 | 1 | MKIILWAVLIIFLIGLLVVTGVFKMIF |
| Escherichia coli | Chromosome | 4226529 | 4226628 | + | 0 | 99 | 0.9998 | 1 | MTALLRVISLVVISVVVIIIPPCGAALGRGKA |
| Escherichia coli | Chromosome | 1084150 | 1084318 | + | 1 | 168 | 1 | 1 | MKRQKRDRLERAHQRGYQAGIAGRSKEMCPYQTLNQRSQWLGGWREAMADRVVMA |
| Escherichia coli | Chromosome | 1201888 | 1202062 | + | 1 | 174 | 1 | 1 | MAVQQNKPTRSKRGMRRSHDALTAVTSLSVDKTSGEKHLRHHITADGYYRGRKVIAK |
| Escherichia coli | Chromosome | 4159855 | 4159996 | + | 1 | 141 | 1 | 1 | MKRTFQPSVLKRNRSHGFRARMATKNGRQVLARRRAKGRARLTVSK |
| Escherichia coli | Chromosome | 780428 | 780542 | + | 2 | 114 | 0.9999 | 1 | MWYFAWILGTLLACSFGVITALALEHVESGKAGQEDI |
| Escherichia coli | Chromosome | 855290 | 855356 | + | 2 | 66 | 1 | 1 | METFCYMKWPVRHHKSRRVSH |
| Escherichia coli | Chromosome | 2376644 | 2376752 | + | 2 | 108 | 1 | 1 | MRIAKIGVIALFLFMALGGIGGVMLAGYTFILRAG |
| Escherichia coli | Chromosome | 4241564 | 4241666 | + | 2 | 102 | 1 | 1 | MRSEQISGSSLNPSCRFSSAYSPVTRQRKDMSR |
| Escherichia coli | Chromosome | 4739099 | 4739225 | + | 2 | 126 | 1 | 1 | MMKRLIVLVLLASTLLTGCNTARGFGEDIKHLGNSISRAAS |
| Escherichia coli | Chromosome | 3257972 | 3258116 | - | 0 | 144 | 0.9998 | 1 | MNRCLLLNLSHRSGEDSFPALCISALHTCRCYTHLGASQDSRAGYSY |
| Escherichia coli | Chromosome | 758939 | 759047 | - | 0 | 108 | 0.9944 | 1 | MRKSYEVGISPKINLCNSVEVLTNSFGTVISGRQV |
| Escherichia coli | Chromosome | 338075 | 338177 | - | 0 | 102 | 1 | 1 | MKENKVQQISHKLINIVVFVAIVEYAYLFLHFY |
| Escherichia coli | Chromosome | 2882038 | 2882194 | - | 1 | 156 | 1 | 1 | MIRLQHDKQKQMRYGTLQKRDTLTLCLLKLQLMEWRFDSAWKFGLGRLYLG |
| Escherichia coli | Chromosome | 1668793 | 1668931 | - | 1 | 138 | 1 | 1 | MKSNRQARHILGLDHKISNQRKIVTEGDKSSVVNNPTGRKRPAEK |
| Escherichia coli | Chromosome | 857827 | 857956 | - | 1 | 129 | 1 | 1 | MNEFKRCMRVFSHSPFKVRLMLLSMLCDMVNNKPQQDKPSDK |
| Escherichia coli | Chromosome | 4350363 | 4350474 | - | 2 | 111 | 0.9999 | 1 | MLESIINLVSSGAVDSHTPQTAVAAVLCAAMIGLFS |
| Escherichia coli | Chromosome | 4082763 | 4082931 | - | 2 | 168 | 1 | 1 | MAKGIREKIKLVSSAGTGHFYTTTKNKRTKPEKLELKKFDPVVRQHVIYKEAKIK |
| Escherichia coli | Chromosome | 3717243 | 3717360 | - | 2 | 117 | 1 | 1 | MKVRASVKKLCRNCKIVKRDGVIRVICSAEPKHKQRQG |
| Escherichia coli | Chromosome | 1617312 | 1617462 | - | 2 | 150 | 1 | 1 | MKHNPLVVCLLIICITILTFTLLTRQTLYELRFRDGDKEVAALMACTSR |
| Salmonella enterica | Chromosome | 1855557 | 1855620 | + | 1 | 63 | 0.9998 | 1 | MGQFFAYATAFAVKENDHVA |
| Salmonella enterica | Chromosome | 3915252 | 3915339 | + | 1 | 87 | 0.9999 | 1 | MSHIVRFTGLLLLNAFIVRGRPVGGIQH |
| Salmonella enterica | Chromosome | 4780288 | 4780393 | + | 2 | 105 | 0.9999 | 1 | MPYTKSFSLYQGGKKTNPDELRKSTQPTRIQVEG |
| Salmonella enterica | Chromosome | 47213 | 47312 | + | 3 | 99 | 0.9999 | 1 | MNPSMLNATLLTTAPSRAVVVVRVVVVVGNAP |
| Salmonella enterica | Chromosome | 455702 | 455819 | + | 3 | 117 | 0.9999 | 1 | MKVRASVKKLCRNCKIVKRDGVIRVICSAEPKHKQRQG |
| Salmonella enterica | Chromosome | 4853597 | 4853738 | - | 1 | 141 | 0.9999 | 1 | MKRTFQPSVLKRNRSHGFRARMATKNGRQVLARRRAKGRARLTVSK |
| Salmonella enterica | Chromosome | 3248561 | 3248675 | - | 1 | 114 | 0.9999 | 1 | MWYFAWILGTLLACAFGIITALALEHVEAGKTGQEES |
| Salmonella enterica | Chromosome | 2697350 | 2697395 | - | 1 | 45 | 0.9998 | 1 | MNAAIFRFFFYFST |
| Salmonella enterica | Chromosome | 1185122 | 1185170 | - | 1 | 48 | 0.9998 | 1 | MKLTRFFFAFFFIFP |
| Salmonella enterica | Chromosome | 240457 | 240598 | - | 2 | 141 | 0.9999 | 1 | MTYSPVAPEEWLKAGITDELIRLSAGPEDPDDIIHDLERASRKAAF |
| Salmonella enterica | Chromosome | 4794873 | 4794972 | - | 3 | 99 | 0.9999 | 1 | MTALLRVISLVVISVVVIIIPPCGAALGRGKA |
| Salmonella enterica | Chromosome | 4323489 | 4323636 | - | 3 | 147 | 0.9999 | 1 | MVKKTIAAIFSVLVLSTVLTACNTTRGVGEDISDGGSAISGAATRAQQ |
| Salmonella enterica | Chromosome | 4224570 | 4224669 | - | 3 | 99 | 0.9999 | 1 | MRIIRGANYLLTGGDMDPEPTPLPRWRIFLFR |
