# Supplementary material for: RiboMicrobe: An Integrated Translatome Atlas for Microorganism
Source: Adv Sci (Weinh). 2025 Oct 13;12(48):e09877. doi: 10.1002/advs.202509877 (PMC12752654; doi:10.1002/advs.202509877)
Supplement: Supplementary file 2 — Supplemental Table S1–S6 [file ADVS-12-e09877-s002.zip › re_Table S4.docx]

**Table S4. Detailed result of differential translation efficiency for DiffTE functionality.**

| **Gene ID** | **WT-A**  **(SRX5347624)** | **WT-B**  **(SRX5347625)** | | ***efp* mutant -A**  **(SRX5347627)** | | ***efp* mutant -B**  **(SRX5347628)** | | **Log2 (Fold change)** | | **p-value** | | **FDR** | |
| --- | --- | --- | --- | --- | --- | --- | --- | --- | --- | --- | --- | --- | --- |
| BSU_00240 | 3.89 | 3.876 | | 5.719 | | 6.835 | | 2.395 | | 0.015 | | 0.639 | |
| BSU_00780 | 2.369 | 2.168 | | 4.575 | | 4.209 | | 2.124 | | 0.002 | | 0.557 | |
| BSU_00910 | 21.976 | 22.846 | | 33.242 | | 44.329 | | 16.374 | | 0.036 | | 0.658 | |
| BSU_02390 | 27.227 | 33.453 | | 17.157 | | 8.33 | | -17.596 | | 0.028 | | 0.658 | |
| BSU_02590 | 3.432 | 4.704 | | 1.144 | | 2.197 | | -2.397 | | 0.039 | | 0.658 | |
| BSU_02830 | 18.232 | 11.423 | | 5.719 | | 4.486 | | -9.725 | | 0.041 | | 0.658 | |
| BSU_04210 | 10.534 | 10.471 | | 4.902 | | 1.972 | | -7.066 | | 0.01 | | 0.639 | |
| BSU_04690 | 22.183 | 17.705 | | 11.178 | | 10.822 | | -8.944 | | 0.016 | | 0.639 | |
| BSU_04870 | 3.241 | 2.856 | | 11.438 | | 15.379 | | 10.36 | | 0.008 | | 0.639 | |
| BSU_05820 | 5.073 | 4.341 | | 1.906 | | 1.994 | | -2.757 | | 0.003 | | 0.632 | |
| BSU_06550 | 0.81 | 1.269 | | 2.542 | | 2.848 | | 1.655 | | 0.007 | | 0.639 | |
| BSU_06920 | 4.052 | 4.704 | | 1.452 | | 2.534 | | -2.384 | | 0.02 | | 0.639 | |
| BSU_07600 | 2.431 | 1.713 | | 7.625 | | 7.69 | | 5.585 | | 0 | | 0.359 | |
| BSU_08280 | 6.321 | 6.473 | | 3.177 | | 2.734 | | -3.441 | | 0.001 | | 0.359 | |
| BSU_08350 | 16.336 | 17.297 | | 14.078 | | 11.677 | | -3.94 | | 0.034 | | 0.658 | |
| BSU_08960 | 12.155 | 11.862 | | 10.485 | | 9.144 | | -2.194 | | 0.031 | | 0.658 | |
| BSU_10420 | 3.444 | 4.16 | | 6.629 | | 5.238 | | 2.131 | | 0.046 | | 0.658 | |
| BSU_10470 | 4.862 | 4.08 | | 1.271 | | 1.282 | | -3.195 | | 0.003 | | 0.557 | |
| BSU_10830 | 2.992 | 2.942 | | 1.346 | | 1.375 | | -1.607 | | 0.001 | | 0.359 | |
| BSU_11450 | 6.483 | 8.063 | | 2.288 | | 3.588 | | -4.335 | | 0.014 | | 0.639 | |
| BSU_11850 | 5.281 | 4.699 | | 8.142 | | 6.816 | | 2.489 | | 0.026 | | 0.658 | |
| BSU_11880 | 1.716 | 1.379 | | 4.902 | | 3.076 | | 2.442 | | 0.05 | | 0.662 | |
| BSU_11940 | 2.165 | 2.176 | | 3.481 | | 4.373 | | 1.756 | | 0.019 | | 0.639 | |
| BSU_12220 | 1.945 | 2.181 | | 3.091 | | 4.272 | | 1.619 | | 0.049 | | 0.662 | |
| BSU_12230 | 8.979 | 8.711 | | 6.721 | | 7.342 | | -1.814 | | 0.009 | | 0.639 | |
| BSU_12460 | 8.812 | 7.249 | | 2.709 | | 4.486 | | -4.433 | | 0.02 | | 0.639 | |
| BSU_12500 | 5.886 | 6.547 | | 1.76 | | 1.508 | | -4.583 | | 0.001 | | 0.359 | |
| BSU_12510 | 3.039 | 3.013 | | 4.962 | | 4.354 | | 1.632 | | 0.009 | | 0.639 | |
| BSU_12530 | 1.621 | 2.636 | | 11.438 | | 6.408 | | 6.795 | | 0.048 | | 0.658 | |
| BSU_12660 | 6.077 | 4.442 | | 1.82 | | 2.685 | | -3.007 | | 0.029 | | 0.658 | |
| BSU_13590 | 3.718 | 2.856 | | 0.953 | | 1.538 | | -2.041 | | 0.019 | | 0.639 | |
| BSU_13760 | 4.004 | 5.971 | | 8.729 | | 8.622 | | 3.688 | | 0.02 | | 0.639 | |
| BSU_13789 | 4.862 | 5.711 | | 2.86 | | 3.204 | | -2.255 | | 0.01 | | 0.639 | |
| BSU_14071 | 3.71 | 4.277 | | 1.615 | | 1.299 | | -2.537 | | 0.003 | | 0.621 | |
| BSU_15340 | 3.366 | 4.188 | | 5.719 | | 5.696 | | 1.93 | | 0.012 | | 0.639 | |
| BSU_16290 | 13.932 | 12.618 | | 9.347 | | 9.509 | | -3.847 | | 0.006 | | 0.639 | |
| BSU_16299 | 2.199 | 1.922 | | 4.385 | | 3.371 | | 1.817 | | 0.026 | | 0.658 | |
| BSU_16980 | 10.21 | 8.567 | | 4.669 | | 5.895 | | -4.107 | | 0.016 | | 0.639 | |
| BSU_18150 | 5.672 | 8.347 | | 1.471 | | 2.905 | | -4.822 | | 0.03 | | 0.658 | |
| BSU_18530 | 4.322 | 3.173 | | 6.536 | | 7.69 | | 3.365 | | 0.015 | | 0.639 | |
| BSU_19800 | 3.241 | 1.904 | | 5.719 | | 7.69 | | 4.132 | | 0.024 | | 0.658 | |
| BSU_21150 | 0.81 | 1.428 | | 2.86 | | 2.929 | | 1.775 | | 0.008 | | 0.639 | |
| BSU_21639 | 8.335 | 8.567 | | 3.677 | | 5.639 | | -3.793 | | 0.019 | | 0.639 | |
| BSU_22570 | 5.834 | 4.284 | | 0.953 | | 2.136 | | -3.514 | | 0.022 | | 0.653 | |
| BSU_22640 | 6.807 | 6.854 | | 3.431 | | 2.848 | | -3.691 | | 0.001 | | 0.359 | |
| BSU_22980 | 5.065 | 4.401 | | 10.008 | | 9.07 | | 4.806 | | 0.002 | | 0.557 | |
| BSU_23610 | 8.05 | 8.631 | | 5.644 | | 6.995 | | -2.021 | | 0.045 | | 0.658 | |
| BSU_24205 | 0.81 | 1.999 | | 3.217 | | 3.076 | | 1.742 | | 0.04 | | 0.658 | |
| BSU_24450 | 1.646 | 1.561 | | 3.226 | | 3.254 | | 1.636 | | 0.001 | | 0.359 | |
| BSU_26540 | 7.293 | 7.615 | | 2.86 | | 5.126 | | -3.461 | | 0.035 | | 0.658 | |
| BSU_26650 | 1.215 | 0.601 | | 4.448 | | 2.563 | | 2.597 | | 0.05 | | 0.662 | |
| BSU_26830 | 2.431 | 2.142 | | 0.817 | | 0.466 | | -1.645 | | 0.005 | | 0.639 | |
| BSU_28090 | 4.554 | 4.835 | | 2.038 | | 1.79 | | -2.78 | | 0.001 | | 0.359 | |
| BSU_28640 | 31.539 | 33.67 | | 27.479 | | 27.6 | | -5.065 | | 0.01 | | 0.639 | |
| BSU_29280 | 1.621 | 0.571 | | 2.86 | | 4.101 | | 2.384 | | 0.038 | | 0.658 | |
| BSU_31270 | 0.748 | 1.038 | | 3.813 | | 2.698 | | 2.362 | | 0.016 | | 0.639 | |
| BSU_31959 | 2.917 | 3.712 | | 1.32 | | 1.435 | | -1.937 | | 0.011 | | 0.639 | |
| BSU_32090 | 0.695 | 1.142 | | 11.438 | | 7.69 | | 8.645 | | 0.011 | | 0.639 | |
| BSU_32340 | 1.389 | 0.714 | | 3.813 | | 2.99 | | 2.35 | | 0.014 | | 0.639 | |
| BSU_32410 | 5.402 | 5.711 | | 0.817 | | 2.563 | | -3.867 | | 0.013 | | 0.639 | |
| BSU_33250 | 3.646 | 4.896 | | 0.953 | | 1.709 | | -2.94 | | 0.017 | | 0.639 | |
| BSU_33960 | 1.297 | 1.68 | | 2.86 | | 4.394 | | 2.139 | | 0.047 | | 0.658 | |
| BSU_34170 | 2.057 | 1.793 | | 3.354 | | 4.08 | | 1.793 | | 0.013 | | 0.639 | |
| BSU_34740 | 16.747 | 17.134 | | 7.211 | | 10.619 | | -8.026 | | 0.011 | | 0.639 | |
| BSU_35560 | 0.405 | 1.269 | | 2.542 | | 2.563 | | 1.715 | | 0.019 | | 0.639 | |
| BSU_35830 | 0.778 | 1.848 | | 4.289 | | 6.835 | | 4.249 | | 0.033 | | 0.658 | |
| BSU_35870 | 8.103 | 8.567 | | 3.431 | | 0.466 | | -6.387 | | 0.014 | | 0.639 | |
| BSU_36150 | 3.473 | 2.221 | | 1.183 | | 1.068 | | -1.721 | | 0.046 | | 0.658 | |
| BSU_36200 | 4.862 | 3.712 | | 1.69 | | 1.491 | | -2.697 | | 0.012 | | 0.639 | |
| BSU_36500 | 6.483 | 8.567 | | 14.298 | | 15.379 | | 7.314 | | 0.005 | | 0.639 | |
| BSU_36920 | 20.815 | 16.76 | | 13.112 | | 13.294 | | -5.585 | | 0.043 | | 0.658 | |
| BSU_37280 | 1.215 | 1.666 | | 3.119 | | 4.101 | | 2.17 | | 0.017 | | 0.639 | |
| BSU_37740 | 2.431 | 1.904 | | 11.438 | | 7.69 | | 7.396 | | 0.017 | | 0.639 | |
| BSU_37780 | 4.482 | 4.413 | | 2.86 | | 2.501 | | -1.768 | | 0.003 | | 0.557 | |
| BSU_37890 | 3.241 | 1.904 | | 5.719 | | 5.126 | | 2.85 | | 0.018 | | 0.639 | |
| BSU_37950 | 24.067 | 18.721 | | 8.621 | | 12.461 | | -10.853 | | 0.027 | | 0.658 | |
| BSU_37980 | 8.306 | 7.547 | 5.53 | | 6.431 | | -1.946 | | 0.029 | | 0.658 | |  |
| BSU_38018 | 21.879 | 17.574 | 12.478 | | 6.664 | | -10.155 | | 0.041 | | 0.658 | |  |
| BSU_39260 | 9.96 | 10.196 | 7.519 | | 7.996 | | -2.32 | | 0.003 | | 0.557 | |  |
| BSU_39360 | 10.939 | 8.159 | 2.86 | | 0.932 | | -7.654 | | 0.012 | | 0.639 | |  |
| BSU_39470 | 7.64 | 13.327 | 2.288 | | 2.848 | | -7.916 | | 0.042 | | 0.658 | |  |
| BSU_39520 | 1.215 | 2.176 | 4.003 | | 3.418 | | 2.015 | | 0.024 | | 0.658 | |  |
| BSU_39690 | 2.076 | 2.764 | 4.464 | | 3.693 | | 1.658 | | 0.032 | | 0.658 | |  |
| BSU_40040 | 14.586 | 19.582 | 4.766 | | 4.272 | | -12.565 | | 0.009 | | 0.639 | |  |
| BSU_40210 | 1.848 | 2.029 | 3.119 | | 4.486 | | 1.864 | | 0.047 | | 0.658 | |  |
| BSU_40810 | 0.935 | 2.197 | 4.575 | | 4.596 | | 3.02 | | 0.011 | | 0.639 | |  |
| BSU_40850 | 8.752 | 8.25 | 4.766 | | 4.272 | | -3.982 | | 0.001 | | 0.359 | |  |
| BSU_40870 | 2.431 | 3.046 | 1.144 | | 0.256 | | -2.038 | | 0.021 | | 0.639 | |  |
